# Osteology of the skull of *Tropidosuchus romeri* (Archosauriformes: Proterochampsidae)

**DOI:** 10.1098/rsos.250248

**Published:** 2025-06-11

**Authors:** Claudio A. Mamani, M. Jimena Trotteyn, Fernando E. Novas, Julia B. Desojo, Martín D. Ezcurra

**Affiliations:** ^1^ Instituto de Geología Emiliano P. Aparicio INGEO-CIGEOBIO, Universidad Nacional de San Juan, Facultad de Ciencias Exactas Físicas y Naturales, San Juan, San Juan Province, Argentina; ^2^ Consejo Nacional de Investigaciones Científicas y Técnicas, Ciudad Autónoma de Buenos Aires, Argentina; ^3^ Departamento de Ciencias Naturales y Antropología, Fundación de Historia Natural ‘Félix de Azara’, Universidad Maimónides, Ciudad Autónoma de Buenos Aires, Argentina; ^4^ División Paleovertebrados, Universidad Nacional de la Plata, La Plata, Buenos Aires Province, Argentina; ^5^ Sección Paleontología de Vertebrados, Museo Argentino de Ciencias Naturales 'Bernardino Rivadavia', Ciudad Autónoma de Buenos Aires, Argentina; ^6^ School of Geography, Earth and Environmental Sciences, University of Birmingham, Birmingham, UK

**Keywords:** Proterochampsia, Rhadinosuchinae, Triassic, Carnian, Chañares Formation, Osteology

## Abstract

*Tropidosuchus romeri* is a member of the clade Proterochampsidae (non-Archosauria Archosauriformes) collected from Upper Triassic (lower Carnian) outcrops of the Chañares Formation of the Ischigualasto-Villa Unión Basin (La Rioja Province, Argentina). This species is a non-rhadinosuchine proterochampsid known from several specimens, including almost complete skeletons. Here, we describe and compare in detail the skull of *T. romeri* based on direct observation of specimens and the three-dimensional segmentation of one of them based on computed microtomography scans. Contrasting with previous studies, we were able to determine sutures between bones and individualize each element digitally. *Tropidosuchus romeri* shares with rhadinosuchines the presence of a crest on the lateral surface of the jugal and a main body of the postorbital with a projection onto the orbit. Features such as a short preorbital region (less than half of skull length), well-developed anterolateral processes of the frontals, premaxilla without anteroventrally oriented alveolar margin, a maxilla restricted to the lateral wall of the rostrum, and a skull roof ornamentation composed of crests without a radial arrangement are probably autapomorphies. Future studies will focus on the postcranial anatomy and on an integration of the new information to test quantitatively the phylogenetic relationships of the species.

## 1. Introduction

The Triassic witnessed the recovery of life after the largest mass extinction ever recorded in Earth’s history [1–4]. The aftermath of the extinction is characterized by the diversification of several diapsid clades, including rhynchocephalians, tanysaurians, rhynchosaurs, erythrosuchids, doswelliids, proterochampsids, pseudosuchians and dinosaurs, among others [5–13]. Most of these Triassic diapsid clades reached a cosmopolitan distribution, but the record of the proterochampsid archosauriforms is restricted to South America. Proterochampsids are small to medium-sized quadrupedal predatory forms characterized by skulls with elongated and moderately retracted external and internal nares and an ornamented roof, absence of the postfrontal bone, and robust second and narrow fourth pedal digits, among other features [12,14,15]. Twelve proterochampsid species have been found so far in the Ischigualasto-Villa Unión Basin of northwestern Argentina and the Paraná Basin of southern Brazil [9,16]. Seven species are currently recorded in the Santa Maria Supersequence of the Paraná Basin, namely *Pinheirochampsa rodriguesi* [16], *Kuruxuchampsa dornellesi* [16], *Stenoscelida aurantiacus* [17], *Rhadinosuchus gracilis* [18], *Cerritosaurus binsfeldi* 19], *Retymaijychampsa beckerorum* [20] and *Proterochampsa nodosa* [21]. A slightly lower species-level taxonomic richness is currently known in Argentina, with *Proterochampsa barrionuevoi* [22] and *Pseudochampsa ischigualastensis* [23] from the Ischigualasto Formation, and *Chanaresuchus bonapartei* [24], *Gualosuchus reigi* [24] and *Tropidosuchus romeri* [25] from the older Chañares Formation. Regarding the latter geological unit, the proterochampsids, particularly *Ch. bonapartei*, are the most numerically abundant diapsids of its *Massetognathus–Chanaresuchus* Assemblage Zone (AZ) (*sensu* [26]).

Proterochampsids are particularly important to understand the early evolutionary radiation of Archosauriformes because they have been interpreted as the sister taxon or very closely related to the crown-group Archosauria (e.g. [15,23,27–31]). In recent years, the available information about the anatomy, taxonomy and phylogeny of the proterochampsids has been improved as a result of multiple contributions (e.g. [9,12,15–17,23,32–44]), but there are still substantial gaps in our knowledge of the group.

In particular, one of the proterochampsid species that is known from more complete specimens, but requires a detailed reassessment is *T. romeri*. In 1969, a field crew of the Fundación-Instituto Miguel Lillo in San Miguel de Tucumán, funded by the CONICET, and led by Dr José Bonaparte found several, almost complete to fragmentary, skeletons of *T. romeri* in rocks of the Chañares Formation (current-day Talampaya National Park, La Rioja Province, northwestern Argentina). This genus and species were erected by Arcucci [25], and this contribution included a preliminary description of the anatomy, which was accompanied by schematic line drawings. As a consequence, the original description of *T. romeri* contained little detail beyond its general morphology. Although the completeness of the hypodigm of *T. romeri*, subsequent studies have not reassessed the anatomy of this species, except for its long-bone histology [43] and cranial endocast [39]. *Tropidosuchus romeri* is one of the earliest-diverging proterochampsids, without the highly specialized features present in the genus *Proterochampsa* and the rhadinosuchines. Thus, a detailed anatomical knowledge of *T. romeri* is crucial to shed light on the phylogenetic relationships within Proterochampsia and the affinities of this clade close to the base of Archosauria.

This contribution aims to provide a comprehensive and detailed description of the skull of *T. romeri* based on a first-hand study of the original specimens and mainly on three-dimensional digital renderings obtained from micro-computed tomography (CT) scans of the skull of PVL 4604.

## 2. Material and methods

*Tropidosuchus romeri* currently consists of its type specimen and seven unambiguously referred specimens, representing a total of eight individuals. All the specimens are housed at the Instituto Miguel Ángel Lillo of the Universidad Nacional de Tucumán (San Miguel de Tucumán, Tucumán Province) to the exclusion of one of the referred specimens housed at the Universidad Nacional de La Rioja (La Rioja, La Rioja Province) and one in the Museum of Comparative Zoology at the Harvard University (Boston, USA). The detailed anatomical description of this contribution is based on the three-dimensional rendering of the skull of PVL 4604 and first-hand examination of PVL 4601 (holotype), PVL 4602, PVL 4604, PVL 4606 and PVL 4625b, which have complete to partially preserved skulls.

The first micro-CT scan of PVL 4604 was performed at the facilities of the Facultad de Matemáticas, Astronomía, Física y Computación of the Universidad Nacional de Córdoba, using an X-ray/Bright Speed S Integral System scanner. It resulted in 436 slices in DICOM format with an interslice spacing of 0.194 mm and a pixel resolution of 235 × 235. This scan was used to segment the skull into its individual bones by digitally removing the surrounding sedimentary matrix using the open-source three-dimensional Slicer software version 4.8.1 [45]. The three-dimensional model was exported as OBJ files and then imported into Design Spark Mechanical 5.0 to generate a three-dimensional PDF file. This three-dimensional PDF file (see the electronic supplementary material) was used to generate some of the figures included in this contribution. These figures were edited in Gimp 2.10.22 (digital image editor in bitmap format) and Inkscape 1.0.2 (free vector graphics editor). The bony cavities (endocranial, pneumatic and foramina) were identified and manually filled for subsequent reconstruction.

The second micro-CT scan of PVL 4604 was performed in the facilities of Advanced Machine Systems (AMS) using a Zeiss Metrotom 800 scanner. This scan resulted in 843 slices in DICOM format, with an interslice spacing of 0.05459 mm and a pixel resolution of 584 × 1719. The marginal tooth-bearing bones were isolated from the surrounding matrix after segmentation and visualization were conducted using the same programs mentioned above (see the electronic supplementary material).

### 2.1. Institutional abbreviations

**CRILAR-Pv,** Centro Regional de Investigaciones y Transferencia Tecnológica de La Rioja, Paleontología de Vertebrados, Anillaco, Argentina; **MACN-Pv**, Colección Nacional de Paleovertebrados, Museo Argentino de Ciencias Naturales ‘Bernardino Rivadavia’, Ciudad Autónoma de Buenos Aires, Argentina; **MAP**, Museu Anchieta de Ciências Naturais, Porto Alegre, Brazil; **MCP**, Museu de Ciencias e Tecnología, Pontificia Universidade Catolica, Porto Alegre, Brazil; **MCZ**, Museum of Comparative Zoology, Harvard University, Boston, USA; **PULR-V**, Paleontología, Universidad Nacional de La Rioja, La Rioja, Argentina; **PVL**, Paleontología de Vertebrados, Instituto ‘Miguel Lillo’, San Miguel de Tucumán, Argentina; **PVSJ**, Sección de Paleontología de Vertebrados, Museo de Ciencias Naturales de la Universidad Nacional de San Juan, San Juan, Argentina; **SNSB-BSPG**, Staatliche Naturwissenschaftliche Sammlungen Bayerns-Bayerische Staatssammlungfür Paläontologieund Geologie, Munich, Germany; **UFRGS-PV-T**, Triassic Vertebrate Collection of the Museu de Paleontologia ‘Iraj a Damiani Pinto,’ Universidade Federal de Rio Grande do Sul, Porto Alegre, Brazil; **USNM**, National Museum of Natural History (formerly United States National Museum), Smithsonian Institution, Washington DC, USA.

## 3. Systematic palaeontology

Diapsida Osborn, 1903 [46] (*sensu* [47])

Archosauromorpha von Huene, 1946 [48] (*sensu* [49])

Archosauriformes Gauthier, Kluge and Rowe, 1988 [50]

Proterochampsia Bonaparte, 1971 [51] (*sensu* [52])

Proterochampsidae Sill, 1967 [53] (*sensu* [54])

*Tropidosuchus romeri* Arcucci, 1990 [25]

### 3.1. Holotype

PVL 4601: skull and articulated lower jaw, complete vertebral column with several ribs and osteoderms, right scapula, coracoid, and humerus, pelvic girdle, both femora, tibiae, fibulae, tarsi, and pes, without both fourth digits, and some indeterminate fragments.

### 3.2. Referred specimens

PVL 4602: partial skull and lower jaw, articulated presacral vertebral column, partial right hemipelvis, both femora, and left tibia and fibula; PVL 4603: partial braincase articulated to an almost complete vertebral column and with some associated osteoderms; PVL 4604: skull with lower jaw, complete presacral and sacral vertebral series with several ribs and osteoderms, seven anterior and seven posterior caudal vertebrae, interclavicle, left scapula, coracoid, and clavicle, right humerus, pelvic girdle, right femur and partial tibia and fibula (lacking the distal portions), proximal half of left femur, left tibia, fibula, and astragalus, and partial right pes (see the electronic supplementary material); PVL 4606: skull with articulated lower jaw, almost complete presacral and sacral vertebral series, five anterior and six posterior caudal vertebrae, several ribs and osteoderms, left radius and ulna, left ilium and ischium, partial right femur, left femur, tibia, fibula, tarsus and partial pes; PVL 4625b: posterior region of the skull and lower jaw and cervical vertebrae, ribs and osteoderms; PULR-V unnumbered (associated to PULR-V 08 and 09): middle-posterior dorsal and anterior caudal vertebrae, and right femur, tibia and fibula; MCZ 9482: partial skeleton of two individuals of *T. romeri* associated to a specimen of *Gracilisuchus stipanicicorum* (modified from Ezcurra [15]).

In addition, a mostly unprepared partial skull with at least one rib (PVL 4605); a left scapula and coracoid, and a right humerus, radius and ulna (PVL 4624); disproportionate left femur, radius and ulna, both tibia and a right fibula, scapula and coracoid, plus some undetermined elements (PVL 4603) were referred to *T. romeri* by Arcucci [25] and could also be part of the hypodigm of the species. However, further assessment of these specimens is necessary to determine their referral confidently.

### 3.3. Diagnosis

Trotteyn *et al*. [12] diagnosed *Tropidosuchus* and *T. romeri* as an archosauriform with triangular skull in dorsal view; proportionally large orbits; curved premaxilla at the distal tip; nearly vertical quadrate; well-developed occipital crest; skull ornamentation as longitudinal crests with different disposition; pterygoid with posterior edges of the wings straight and thickened; internal edge of pterygoid with denticles in alveoli, forming a ‘V’-shaped structure opened posteriorly; vertebral cervicodorsal zonation more marked; shoulder girdle with rod-like clavicle and interclavicle; ulna without olecranon; femur as long as the tibia; tibia without transverse or longitudinal distal articulation; and large osteoderms, in one row and one per vertebra, with a well-developed dorsal laminar crest extended axially.

We consider that the diagnosis of *T. romeri* needs updating, but we will provide an emended diagnosis once the complete anatomy of this species is revised.

### 3.4. Geographical and stratigraphic occurrence

All specimens of *T. romeri* come from outcrops at the Los Chañares type locality (= Romer’s northwest pocket), located north of the Chañares River, Talampaya National Park, La Rioja Province, Argentina. The fossils come from the lower member (*sensu* [55]) of the Chañares Formation (*Massetognathus–Chanaresuchus* AZ *sensu* [26]), Ischigualasto-Villa Unión Basin (*ca* 236 Ma, early Carnian, early Late Triassic, [26,56]).

## 4. Description

### 4.1. Skull

**Figure 1.**
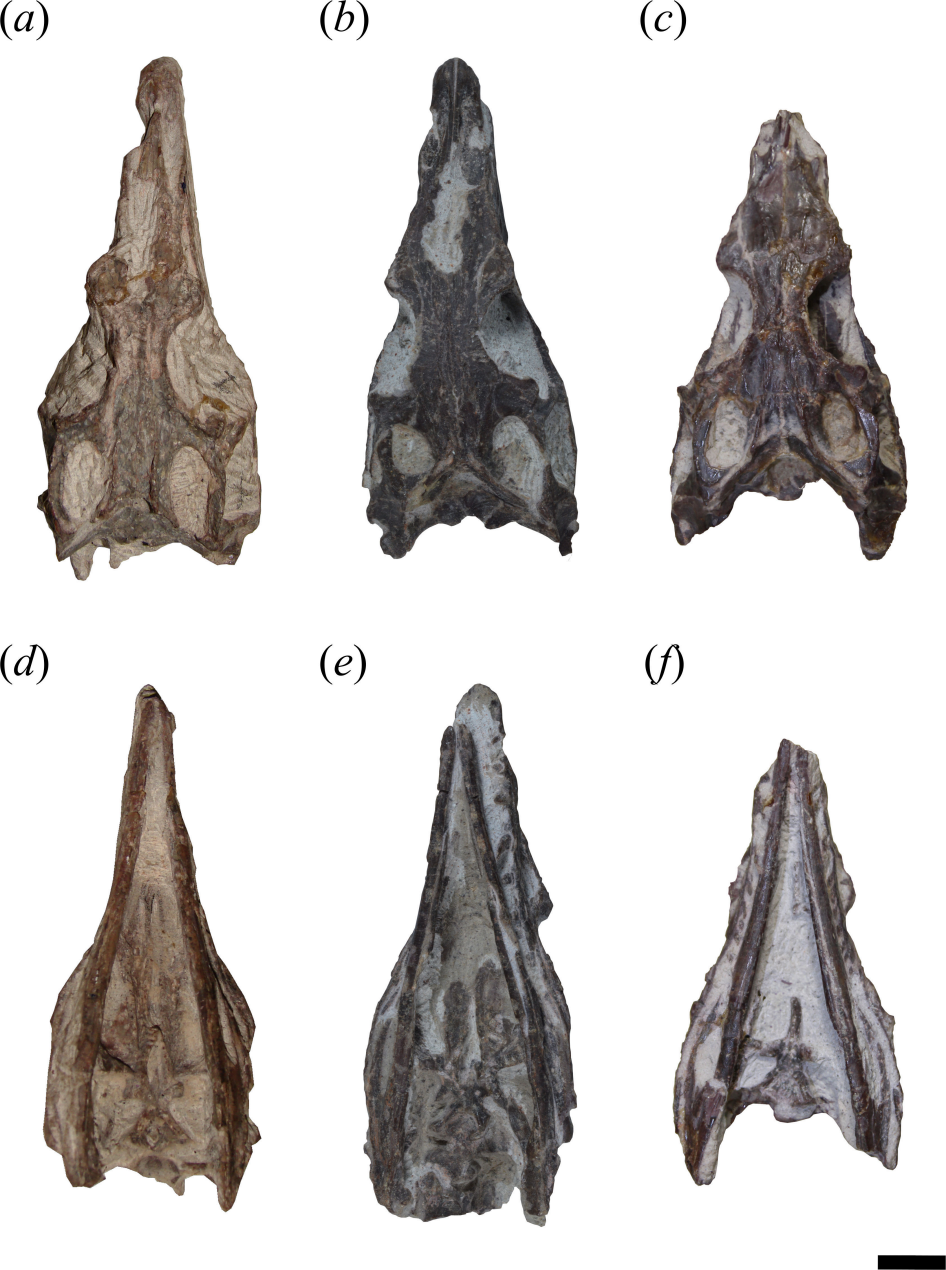
Skull of *Tropidosuchus romeri*. (*a–c*) Dorsal view. (*d–f*) Ventral view. (*a*,*d*: holotype) PVL 4601; (*b,e*) PVL 4604; and (*c,f*) PVL 4606. Scale bars equal 10 mm.

**Figure 2.**
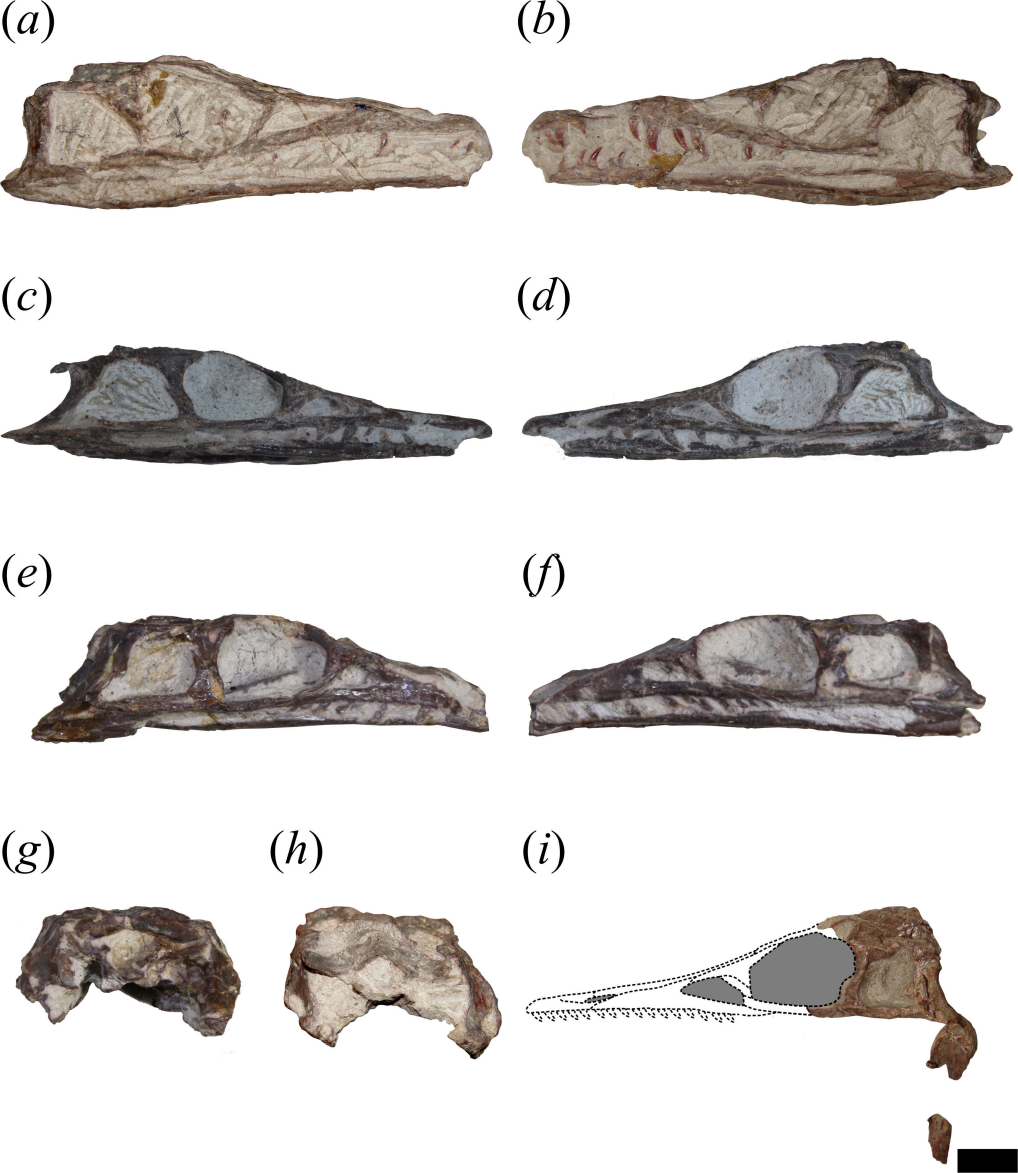
Skull of *Tropidosuchus romeri*. (*a,c,e*) Right lateral view. (*b,d,f,i*) Left lateral view. (*g,h*) Occipital view. (*a,b,h*: holotype) PVL 4601; (*c,d*) PVL 4604; (*e,f,g*) PVL 4606; and (*i*) PVL 4625b. Scale bars equal 10 mm.

**Figure 3.**
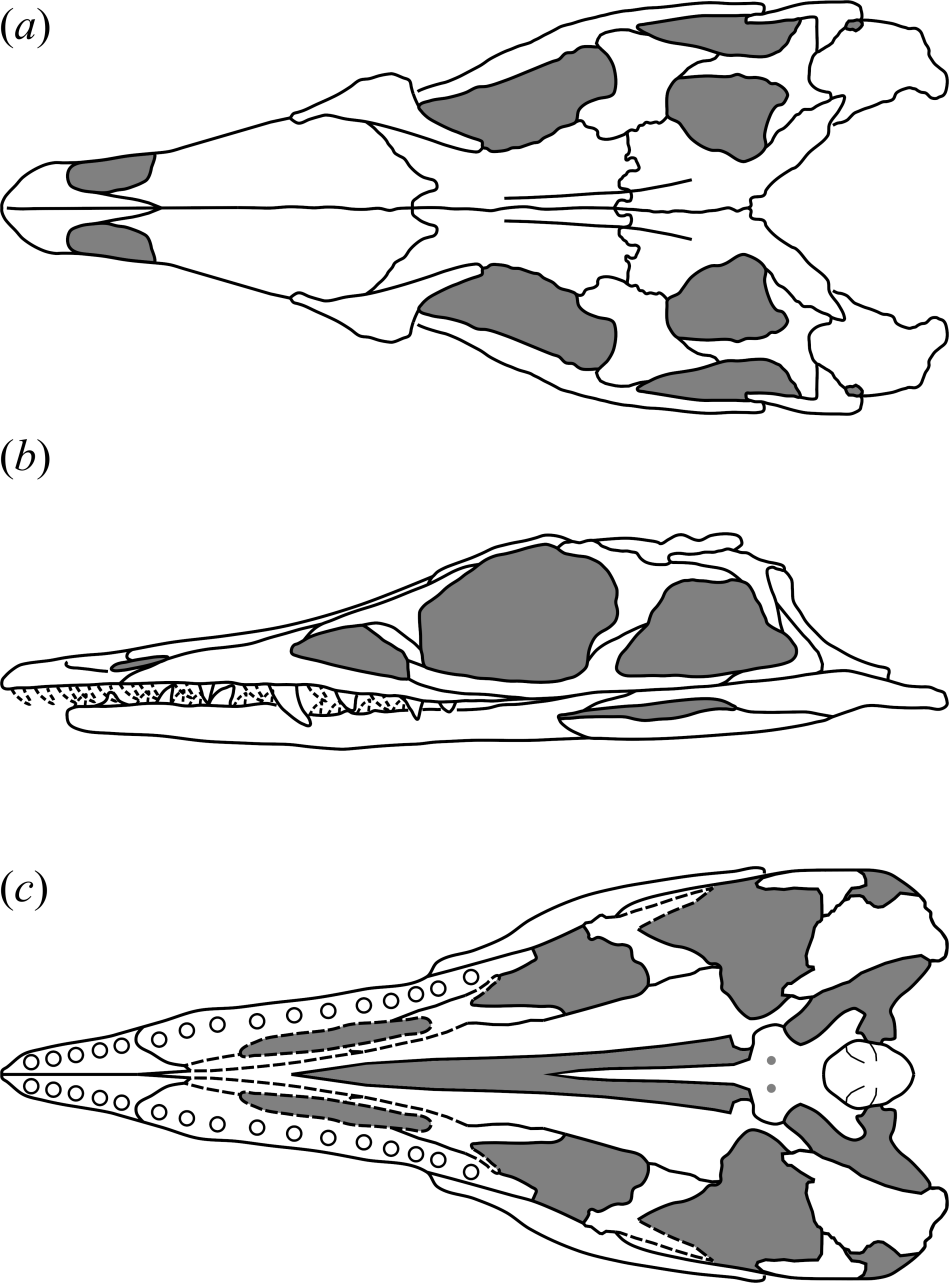
Schematic line drawing of the skull of *Tropidosuchus romeri* based on specimens PVL 4601, PVL 4604 and PVL 4606. (*a*) Dorsal view; (*b*) left lateral view; and (*c*) ventral view.

In general, the skull of *T. romeri* is more similar to that of *Ce. binsfeldi* and rhadinosuchines than to the extremely dorsoventrally low skulls of the genus *Proterochampsa* [12,25,57]. Although some skulls show signs of a certain degree of dorsoventral flattening (e.g. PVL 4604, 4606), in general, the skulls of *T. romeri* are relatively well preserved. In dorsal view, the skull is triangular, being elongated anteroposteriorly (figures 1, 2 and 3; see table 1). The rostrum (= preorbital region) reaches its maximum width at the level of the prefrontal, and its length represents slightly less than 50% of the total skull length. Immediately posterior to the orbits, the skull widens at the level of the lower temporal bar and the width remains constant until the posterior margin. The transverse width achieved by the postorbital region of *T. romeri* is, in comparison with the length of the skull, proportionally lower than in other rhadinosuchine species, such as *Ch. bonapartei* (PULR-V 07: [36]), *Gu. reigi* (PVL 4576) and *Ps. ischigualastensis* (PVSJ 567: [23]).

**Table 1.** Measurements of the skull of *Tropidosuchus romeri* specimen PVL 4604, expressed in millimetres. (Values marked with an asterisk (*) represent incomplete measurements owing to post-mortem damage; the reported value corresponds to the maximum measurable length. The calliper’s maximum deviation is 0.02 mm, but measurements have been rounded to the nearest 0.1 mm.)

	length	height	width
skull	73.1	18.7	33.2
snout	33.3	12.9	19.9
external naris	14.3	—	3.3*
antorbital fenestra	12.2	3.9	—
orbit	17.4	13	—
infratemporal fenestra	16.3	8.9	—
supratemporal fenestra	7.9	—	5.9
mandibular fenestra	14.8	2.9	—
internal choanae	10.9	—	—

**Figure 4.**
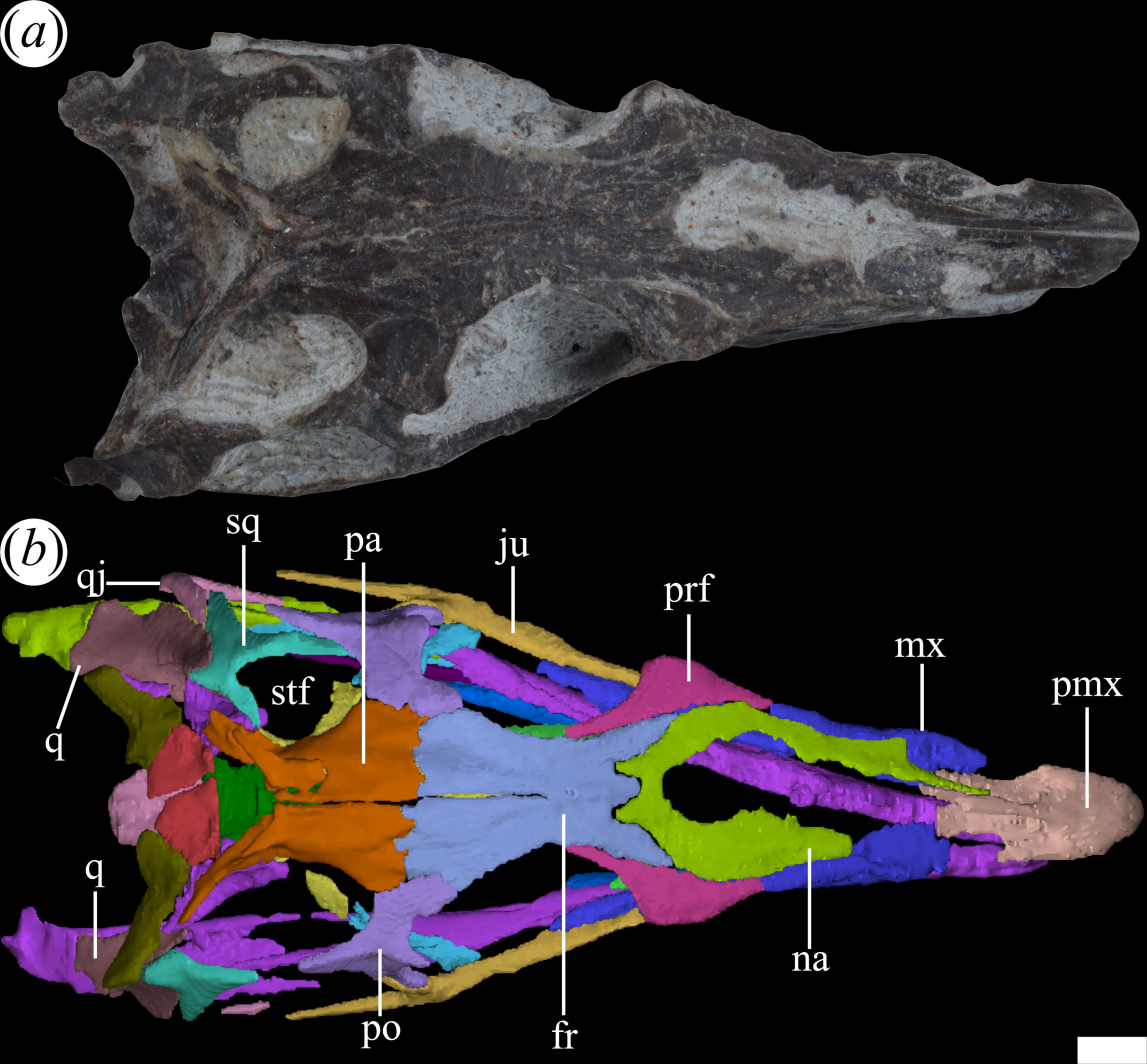
Skull of *Tropidosuchus romeri* in dorsal view. (*a,b*) Referred specimen PVL 4604. fr, frontal; ju, jugal; mx, maxilla; na, nasal; pa, parietal; pmx, premaxilla; po, postorbital; prf, prefrontal; q, quadrate; qj, quadratojugal; sq, squamosal; stf, supratemporal fenestra. Scale bars equal 10 mm.

The external nares are mostly preserved in PVL 4601 and 4604, while in PVL 4606 only the posterior half is preserved (figure 1*a*–*c*). They are paired openings, dorsally oriented and positioned posteriorly to the most anterior tip of the rostrum, as in other proterochampsids [12]. These openings are sub-oval, being anteroposteriorly longer than transversely broad (figure 3*a*). The external nares of *T. romeri* have a maximum length equivalent to 43% of the total length of the rostrum in PVL 4601 and 4604. Their edges are smooth and rounded, especially at the anterior and posterior ends. Both openings are separated by a median bar formed by the articulation of the prenarial process of the premaxillae and the anteromedial process of the nasals (figure 4*b*, pmx, na). These same bones also contribute to the lateral border of the external nares, with a greater contribution from the premaxilla and no contribution from the maxilla.

**Figure 5.**
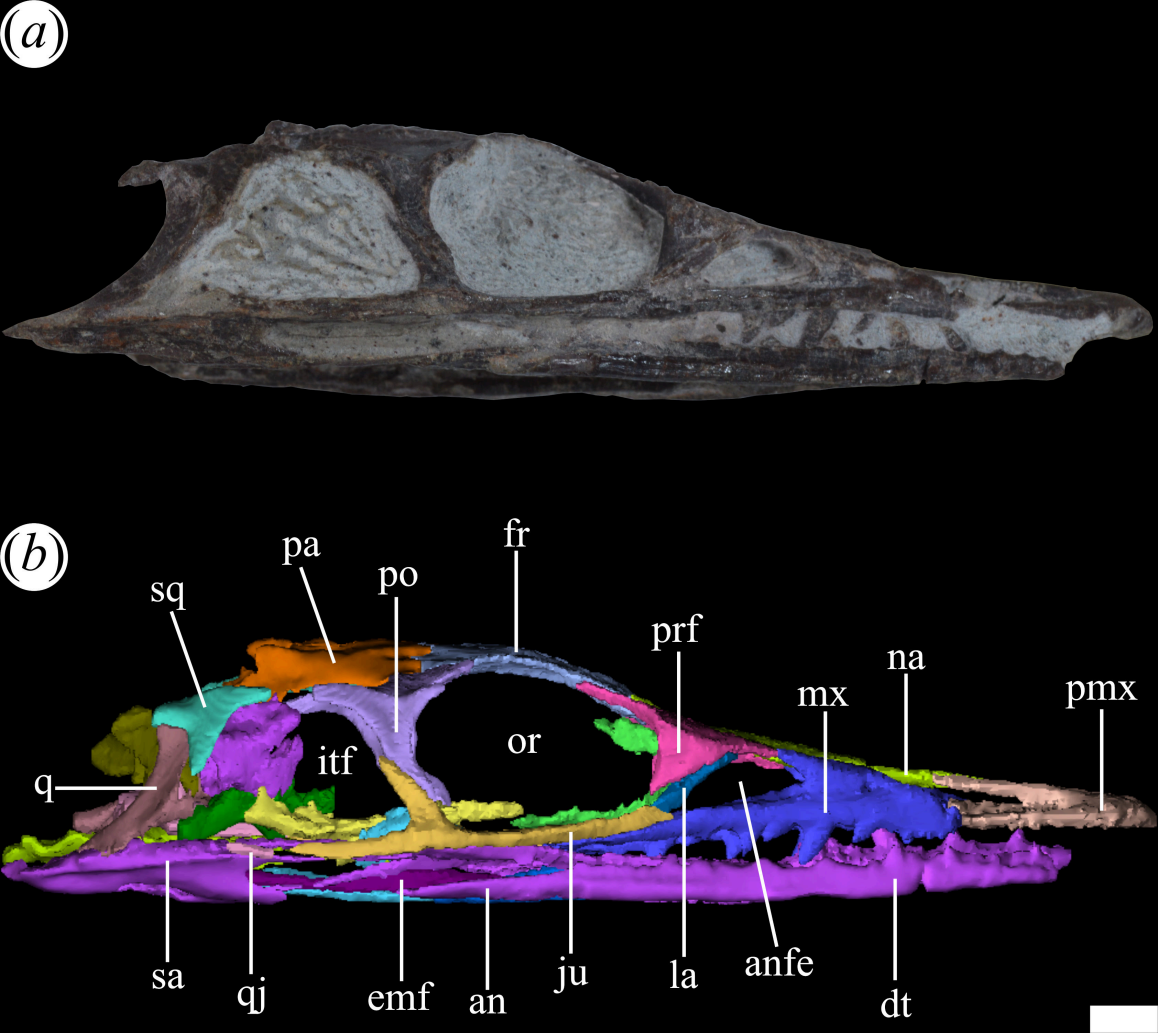
Skull of *Tropidosuchus romeri* in right lateral view. (*a,b*) Referred specimen PVL 4604. an, angular; anfe, antorbital fenestra; dt, dentary; emf, external mandibular fenestra; fr, frontal; itf, infratemporal fenestra; ju, jugal; la, lacrimal; mx, maxilla; na, nasal; or, orbita; pa, parietal; pmx, premaxilla; po, postorbital; prf, prefrontal; q, quadrate; qj, quadratojugal; sa, surangular; sq, squamosal. Scale bars equal 10 mm.

**Figure 6.**
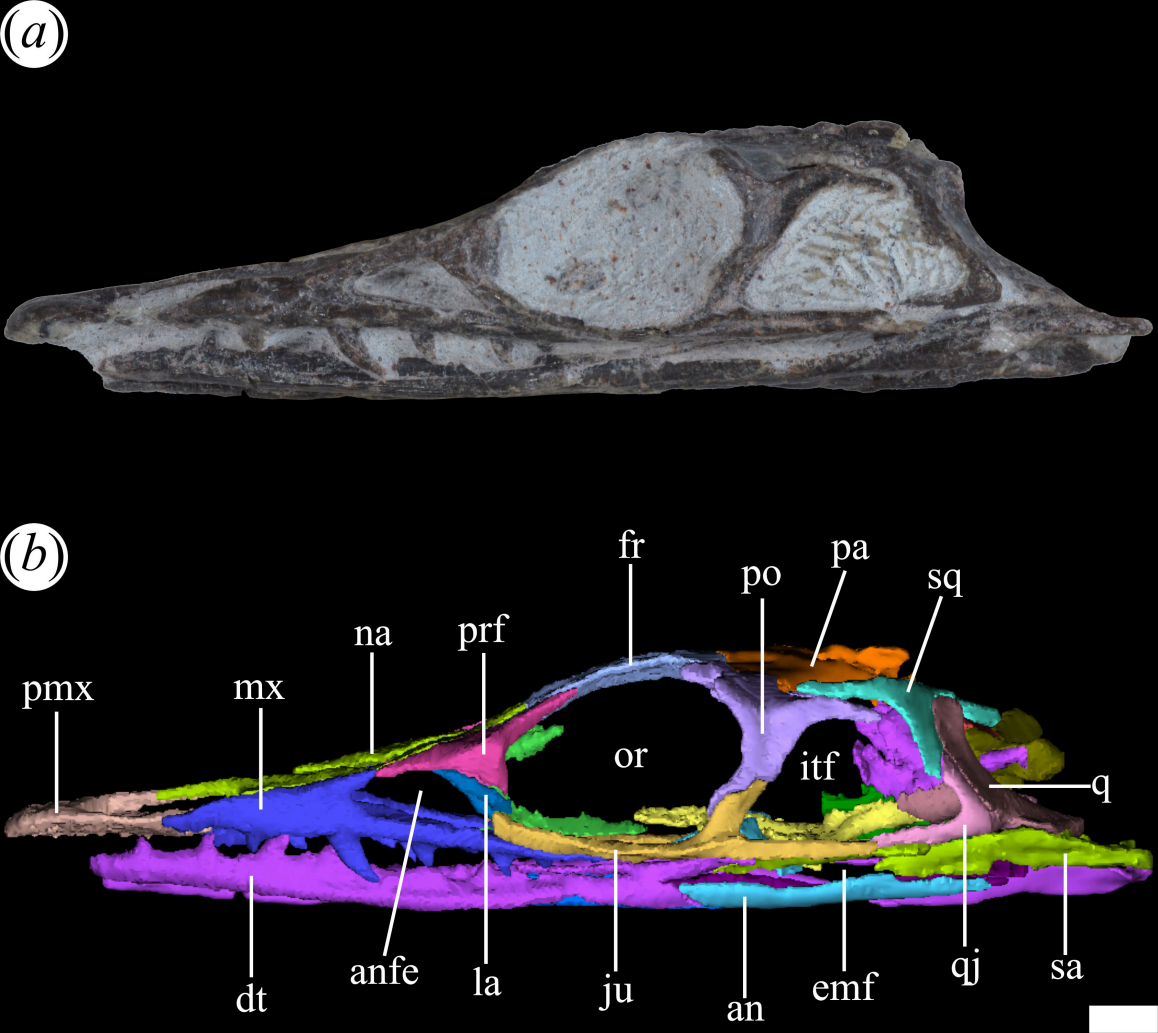
Skull of *Tropidosuchus romeri* in left lateral view. (*a,b*) Referred specimen PVL 4604. an, angular; anfe, antorbital fenestra; dt, dentary; emf, external mandibular fenestra; fr, frontal; itf, infratemporal fenestra; ju, jugal; la, lacrimal; mx, maxilla; na, nasal; or, orbita; pa, parietal; pmx, premaxilla; po, postorbital; prf, prefrontal; q, quadrate; qj, quadratojugal; sa, surangular; sq, squamosal. Scale bars equal 10 mm.

The antorbital fenestra is relatively small and represents 17% of the total length of the skull, which is a ratio considerably lower than that in *Euparkeria capensis* (26.7%: [58]), but resembles that of some non-eucrocopodan archosauriforms such as *Proterosuchus fergusi* and *Erythrosuchus africanus* [59]. However, the antorbital fenestra of *T. romeri* (PVL 4601, 4604) represents 36.6% of the length of the rostrum, which is a ratio higher than in the holotype of *Ch. bonapartei* (26%: PULR-V 07), the referred specimen of *Gu. reigi* (PVL 4576) and an indeterminate rhadinosuchine from the Chañares Formation (CRILAR-Pv 491). These openings are mainly lateralized in all the skulls of *T. romeri* (PVL 4601, 4604, 4606; figure 2*a*–*f*), and they are not exposed in dorsal view, similar to other Proterochampsidae (e.g. *Ce. binsfeldi*: MAP-0657, *Ps. ischigualastensis*: PVSJ 567, *Ch. bonapartei*: PULR-V 07, *Pi. rodriguesi*: [16]). The antorbital fenestra is sub-triangular and has angular corners (figure 3*b*). The edges of this opening are formed by the maxilla, lacrimal and prefrontal, with the maxilla being the most predominant element (figures 5*b* and 6*b*, anfe).

The orbits of *T. romeri* are the largest openings in the entire skull, even surpassing the size of the supratemporal and infratemporal fenestrae (see tables 1 and 2). The subcircular shape of the orbit of *T. romeri* is consistent with that of some specimens of *Ch. bonapartei* and *Gu. reigi* (e.g. PVL 4586 and PULR-V 05, respectively; [59]), but contrasts with the subtriangular to rhomboidal orbit of *Pi. rodriguesi* [16] and also with the oval orbits of *Er. africanus*, *Proterosuchus fergusi* and *’Chasmatosaurus’ yuani*, among non-eucrocopodan archosauriforms [59]. These openings are primarily laterally oriented (figures 2*a*–*f* and 3*b*), although they also have a slight dorsal orientation (figures 1*a*–*c* and 3*a*), similar to *Ch. bonapartei* (PULR-V 07) and *Gu. reigi* (PULR-V 05), but contrasting with the dorsal orientation of the orbits in both species of the genus *Proterochampsa* [33,42]. The orbital margin of *T. romeri* is composed of the jugal, prefrontal, frontal, postorbital and a small contribution from the lacrimal, as in other proterochampsids (figures 5*b* and 6*b*, or).

**Table 2.** Measurements of the skull of *Tropidosuchus romeri* specimen PVL 4606, expressed in millimetres. (Values marked with an asterisk (*) represent incomplete measurements owing to post-mortem damage; the reported value corresponds to the maximum measurable length. The calliper’s maximum deviation is 0.02 mm, but measurements have been rounded to the nearest 0.1 mm.)

	length	height	width
skull	58.6*	17.2	35.6
snout	25.3*	13.4	20.4
antorbital fenestra	11.8	4.1	—
orbit	16.6	11.8	—
infratemporal fenestra	11.1	8.0	—
supratemporal fenestra	10.0	—	7.6
mandibular fenestra	17.8	4.0	—

The infratemporal fenestra stands out as the second largest opening in the skull, a feature that contrasts with most proterochampsids, which is usually the largest opening [12,42,60]. The infratemporal fenestra of *T. romeri* is trapezoidal in lateral view (figures 2*a*–*f*,*i* and 3*b*), being anteroposteriorly longer than dorsoventrally tall, similar to most rhadinosuchines (*Ps. ischigualastensis*: PVSJ 567, *Ch. bonapartei*: PULR-V 07, *Gu. reigi*: PULR-V 05) and some non-eucrocopodan archosauriforms, such as *Proterosuchus fergusi* and *‘Chasmatosaurus’ yuani* [59]. However, in other species, such as *Er. africanus* and *Garjainia prima*, the situation is different and the infratemporal fenestra is dorsoventrally taller than anteroposteriorly long [59]. The infratemporal fenestra of *T. romeri* represents approximately 22.3% of the total length of the skull (PVL 4601, 4604). Additionally, it is an opening that, although dorsolaterally oriented, has a limited dorsal exposure, similar to that observed in *Ce. binsfeldi* [12]. By contrast, in other proterochampsid species, such as *Ch. bonapartei* (PULR-V 07: [36]), *Gu. reigi* (PVL 4576) and *Ps. ischigualastensis* (PVSJ 567: [23]), the infratemporal fenestra possesses a more dorsal orientation, as the ventral temporal bar is more laterally displaced with respect to the upper bar. The infratemporal fenestra of *T. romeri* is delimited by the jugal, quadratojugal, squamosal and postorbital (figures 5b and 6b, itf). The quadratojugal contributes to less than half of the posterior edge of the opening and the rest of this margin is formed by the ventral process of the squamosal, as occurs in all Proterochampsidae [12,16,42] and some other non-archosaurian archosauriforms (e.g. *Er. africanus*, *Eu. capensis*; [59]).

The supratemporal fenestra is fully preserved in the three best-preserved skulls of *T. romeri* (PVL 4601, 4604, 4606; figure 1*a*–*c*). It is fully exposed in dorsal view (figure 3*a*). It is oval, but in *Ps. ischigualastensis* the corners of the supratemporal fenestra are more angular, resulting in a subtriangular shape (PVSJ 567: [23]), as also occurs in *Ce. binsfeldi* [12]. In most specimens of *T. romeri* (PVL 4601, 4604), the major axis of the supratemporal fenestra is parallel to the midline of the skull, but in PVL 4606 this same opening shows a slight inclination from anteromedial to posterolateral (figure 1*c*), similar to *Ch. bonapartei* (PULR-V 07: [36]), *Gu. reigi* (PULR-V 05) and *Pi. rodriguesi* [16]. This orientation of the major axis of the supratemporal fenestra is much more pronounced in both species of *Proterochampsa* [33,42]. The supratemporal fenestra of *T. romeri* is slightly shorter anteroposteriorly compared with the infratemporal fenestra. However, in rhadinosuchines, the supratemporal fenestra can represent approximately 50% of the total length of the infratemporal fenestra, while in both species of *Proterochampsa* [33,42] this ratio equals 25% or is even lower. Three bones surround the supratemporal fenestra: postorbital, parietal and squamosal, with the latter making the greatest contribution as it forms the posterior and lateral edges of the opening (figure 4*b*, stf). In other proterochampsids, the parietal is the predominant bone in the supratemporal fenestra, forming the medial and posterior edges in *Ps. ischigualastensis* (PVSJ 567: [23]), and the medial and half of the anterior edge in *Ch. bonapartei* (PULR-V 07: [36]), *Proterochampsa barrionuevoi* [33] and *Proterochampsa nodosa* [42].

**Figure 7.**
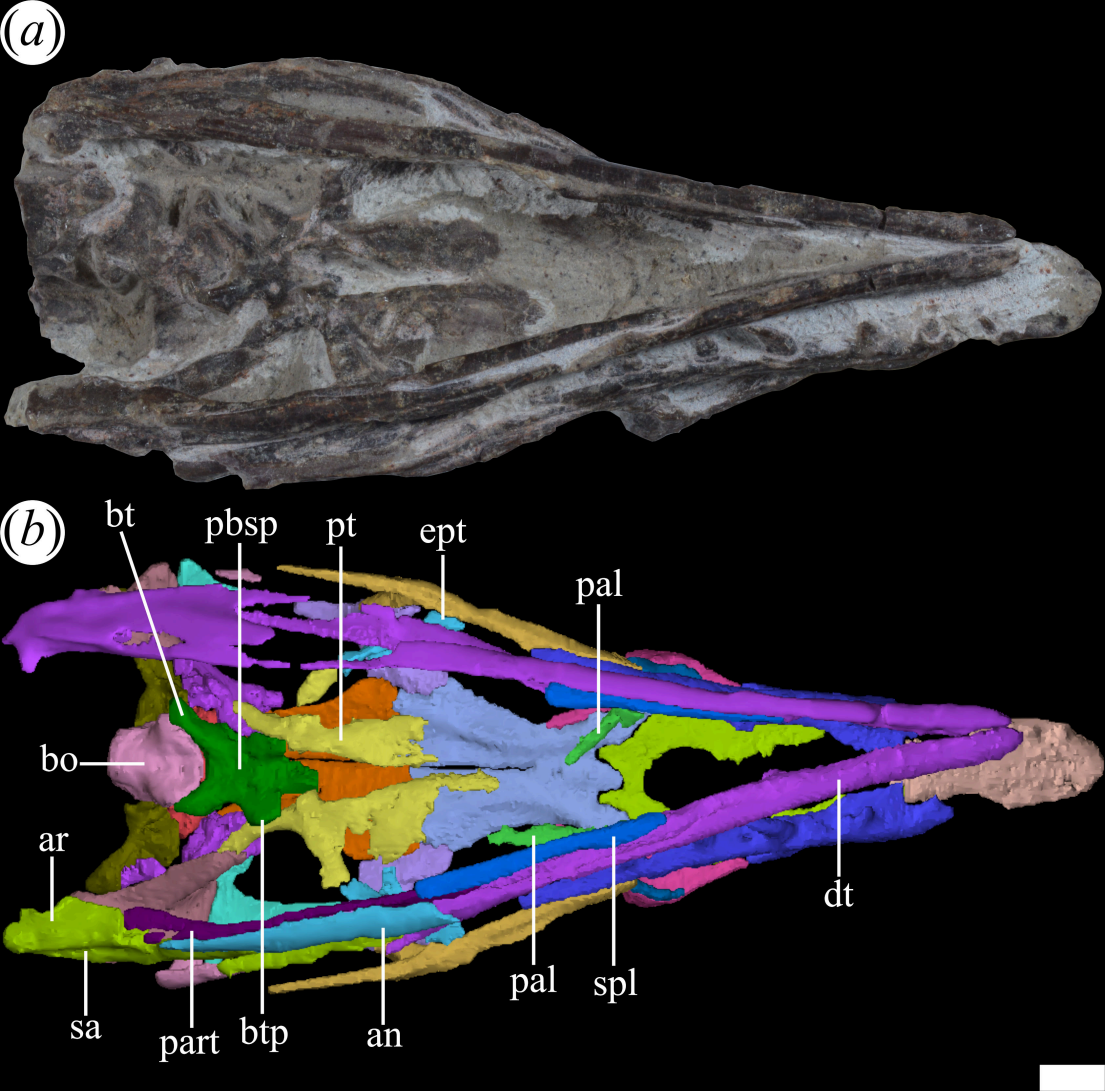
Skull of *Tropidosuchus romeri* in ventral view. (*a,b*) Referred specimen PVL 4604. an, angular; ar, articular; bo, basioccipital; bt, basal tuber; btp, basipterygoid processes; dt, dentary; ept, ectopterygoid; pal, palatine; part, prearticular; pbsp, parabasisphenoid; pt, pterygoid; sa, surangular; spl, splenial. Scale bars equal 10 mm.

The palatines and pterygoids are partially exposed in the skulls of PVL 4601 (holotype) and PVL 4604 in ventral view (figures 1*d*–*f* and 3*c*). The palate of PVL 4606 is not exposed, probably because it is completely covered by matrix (figure 1*f*). Although the palate of PVL 4604 could be reconstructed, almost in its entirety (figure 7*b*, pal), and each bone isolated digitally by segmentation, the interpretation of their morphology and contacts between bones have been limited by poor resolution/contrast of the micro-CT images.

### 4.2. Premaxilla

**Figure 8.**
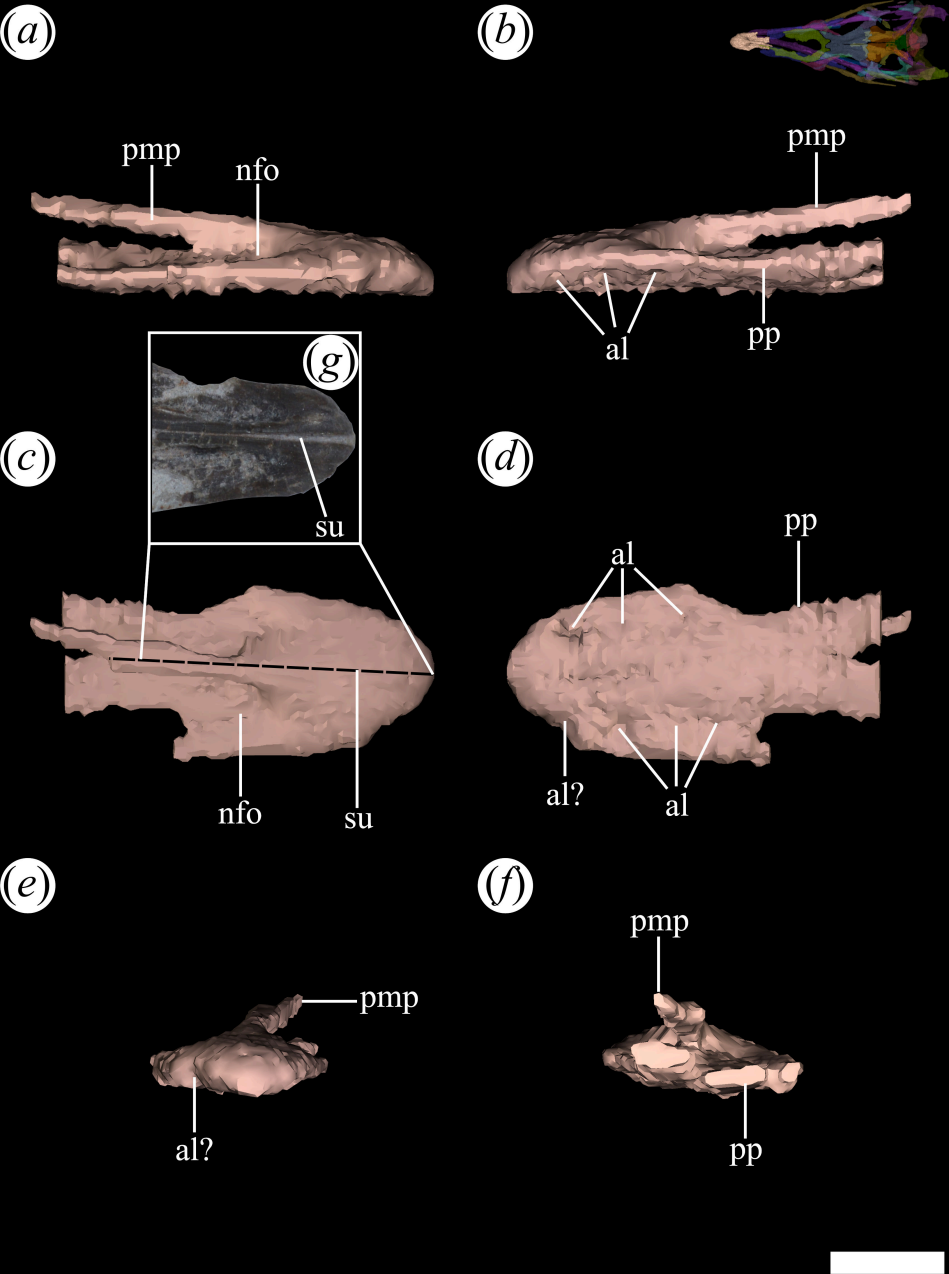
Digital reconstruction of the right and left premaxilla of *Tropidosuchus romeri* (PVL 4604) in (*a*) right lateral view; (*b*) left lateral view; (*c,g*) dorsal view; (*d*) ventral view; (*e*) anterior view; and (*f*) posterior view. al, alveolus; nfo, narial fossa; pmp, posteromedial process; pp, palatal process; su, suture. Scale bars equal 5 mm.

It is the only skull bone that forms part of the rostrum in front of the external nares. The premaxillae are partially preserved in PVL 4601, 4604 and 4606 [25]. Both premaxillae are preserved in the holotype (PVL 4601: [25]), but only the right one is almost complete. In PVL 4606, the premaxillae are very incomplete, represented only by part of their posteromedial process. In PVL 4604, the premaxillae are fairly complete, but with damaged lateral edges. The latter damage prevents the recognition of the morphology of the suture with the maxilla in PVL 4604 (figure 4*b*, pmx). By contrast, in the holotype (PVL 4601: [25]), the lateral edge of the right premaxilla extends dorsally to the anterior process of the maxilla, forming an anteroventrally-to-posterodorsally orientated suture, consistent with the morphology of other Proterochampsidae [12,15]. The lateral edge of the external nares is exclusively formed by the premaxilla, which posteriorly contacts the nasals, excluding any contribution of the maxilla to these openings, as observed in other Proterochampsidae [15]. The premaxilla also forms the anterior margin of the external nares. The medial margin of the external nares is partially formed by the posteromedial (= prenarial) process of the premaxilla (figure 8*a*,*b*,*e*,*f**,* pmp) and a longer contribution of the anteromedial process of the nasal. The posteromedial process of the premaxilla tapers distally and extends, with its counterpart, between the anteromedial processes of the nasal along the median line. The posteromedial process does not extend posteriorly beyond the level of the posterior edge of the external nares (PVL 4604), contrasting with the longer process of *Litorosuchus somnii* [61] that extends beyond this level.

The premaxillary body is strongly elongated anteroposteriorly and its lateral expansion is not as evident as in *Pi. rodriguesi* (UFRGS-PV-0464-T: [16]) and the doswelliid *Rugarhynchos sixmilensis* [62]. The premaxillary body of PVL 4604 is transversely broader than dorsoventrally tall, but the degree of dorsoventral compression of the bone seems to be exaggerated by some postmortem deformation. A slightly dorsoventrally taller, but still transversely broader, premaxillary body is present in PVL 4601 (holotype). The premaxilla of *T. romeri* lacks the slight ventral orientation in lateral view that is characteristic of other proterochampsids (e.g. *Proterochampsa barrionuevoi*: PVSJ 77; *Ch. bonapartei*: PULR-V 07; *Gu. reigi*: PULR-V 05; *Rh. gracilis*: BSPG AS XXV 50, 51; [15]). The external surface of the premaxilla lacks ornamentation, contrasting with the extensively ornamented premaxillary surface of both species of *Proterochampsa* [33,42]. At the anterior end of the external nares, a distinct narial fossa invades the external surface of the premaxilla (figure 8*c**,* nfo), similar to that observed in *Ps. ischigualastensis* (PVSJ 567: [23]), but contrasting with the deeper narial fossa present in some specimens of *Ch. bonapartei* (PULR-V 07: [36]). The median suture between the premaxillae could not be reconstructed in the digital model of the skull owing to the low contrast of the micro-CT in that region. However, this suture can be identified in the real specimen as being straight and anteroposteriorly oriented (figure 8*g**,* su). As a result, both posteromedial processes of the premaxilla articulate extensively along the midline, as in other Proterochampsidae (e.g. *Ps. ischigualastensis*: PVSJ 567; *Ch. bonapartei*: PULR-V 07; *Gu. reigi*: PULR-V 05).

The occlusion of the lower jaw hampers access to the ventral and medial surfaces of the premaxilla. Nevertheless, an extensive palatal process can be observed through the external nares and the three-dimensional model (figure 8*b*,*d*,*f**,* pp). This process is transversely wide and oriented slightly posterolaterally in ventral view, making posterior contact with the palatal process of the maxilla. However, it lacks a mound-shaped medial crest, unlike the condition seen in *Ch. bonapartei*, *Gu. reigi* and *Pi. rodriguesi* [16]. Posteriorly, the pair of palatal processes of the premaxilla appear to diverge from each other, and the unpreserved vomers probably extended anteriorly between them along the median line. The palatal processes of the premaxilla separate the external nares from the internal ones.

**Figure 9.**
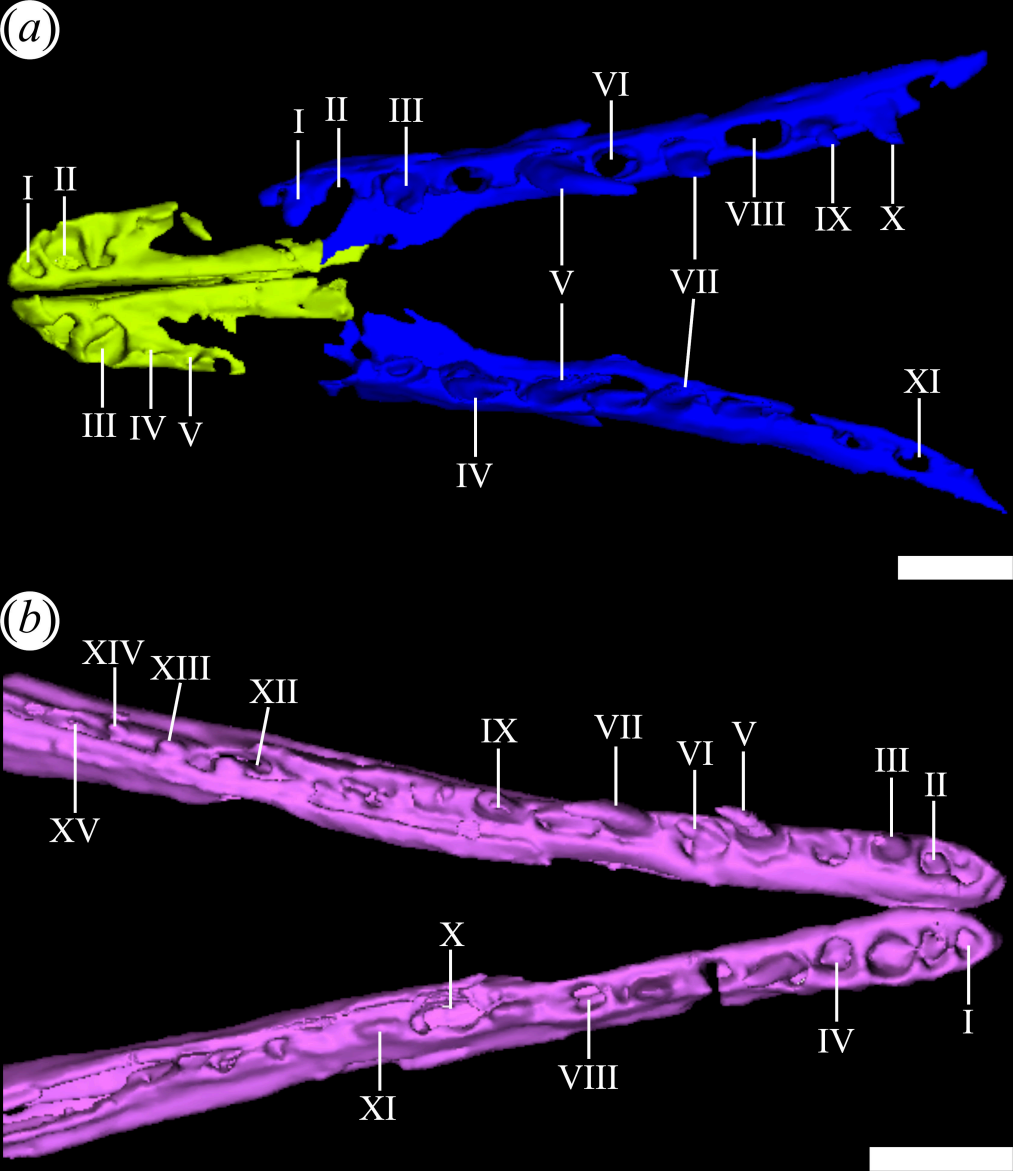
Digital reconstruction of the premaxilla (green), maxilla (blue) and mandible (pink) of *Tropidosuchus romeri* (PVL 4604) in (*a*) ventral view; and (*b*) dorsal view. Roman numerals indicate the different recognized tooth positions. Scale bars equal 5 mm.

The number of tooth positions in the premaxillae cannot be determined with certainty. At least four teeth can be recognized in PVL 4601 (holotype), but their shape is obscured by matrix. The first micro-CT scan of PVL 4604 shows the absence of preserved teeth in the premaxilla and, although the number of alveoli could not be determined, some of them were identified (figure 8*b*,*d**,* al). In a second micro-CT scan of the same specimen, but with better contrast, it was possible to recognize at least five tooth positions in the right premaxilla and four in the left element (figure 9*a*). The presence of five tooth positions in the premaxilla of *T. romeri* represents a minimum count, and there is room for a possible sixth position on the posterior end of the alveolar margin of the bone. The premaxillary tooth counts vary considerably among different proterochampsian species, with a minimum of two in *L. somnii* [61] and a maximum of six in *Ps. ischigualastensis* (PVSJ 567; [23]), *Ch. bonapartei* (PULR-V 07; [36]) and *Proterochampsa barrionuevoi* (MCZ 3408; [33]).

### 4.3. Maxilla

The maxilla is an anteroposteriorly elongated bone and the most broadly exposed element of the lateral surface of the rostrum. Its contribution to the dorsal surface of the rostrum is non-existent, which contrasts with the condition present in *Ch. bonapartei* (PULR-V 07: [36]), *Gu. reigi* (PULR-V 05; PVL 4576: [24]) and *Rh. gracilis* (BSPG AS XXV 50: [37]). It is important to note the box shape of the rostrum of *T. romeri*, a condition that has been considered a synapomorphy of Rhadinosuchinae [37,52]. The suture between the maxilla and the nasal is relatively straight along its entire length. The anterior third of the maxillae of *T. romeri* (PVL 4601, 4604, 4606: [25]) are straight in ventral view, and posteriorly, they curve laterally to diverge more conspicuously from each other at the level of the antorbital fenestra. Both maxillae reach their maximum separation at the level of the orbits. A similar condition is present in *Ch. bonapartei* (PULR-V 07: [36]) and *Gu. reigi* (PULR-V 05: [24]). However, in other proterochampsid species, this condition varies. The maxillae are approximately parallel to each other up to the level of the orbits in *Ps. ischigualastensis* (PVSJ 567: [23]), where they then diverge abruptly, whereas both maxillae diverge conspicuously from each other immediately posterior to the suture with premaxilla in both species of *Proterochampsa* [33,42].

**Figure 10.**
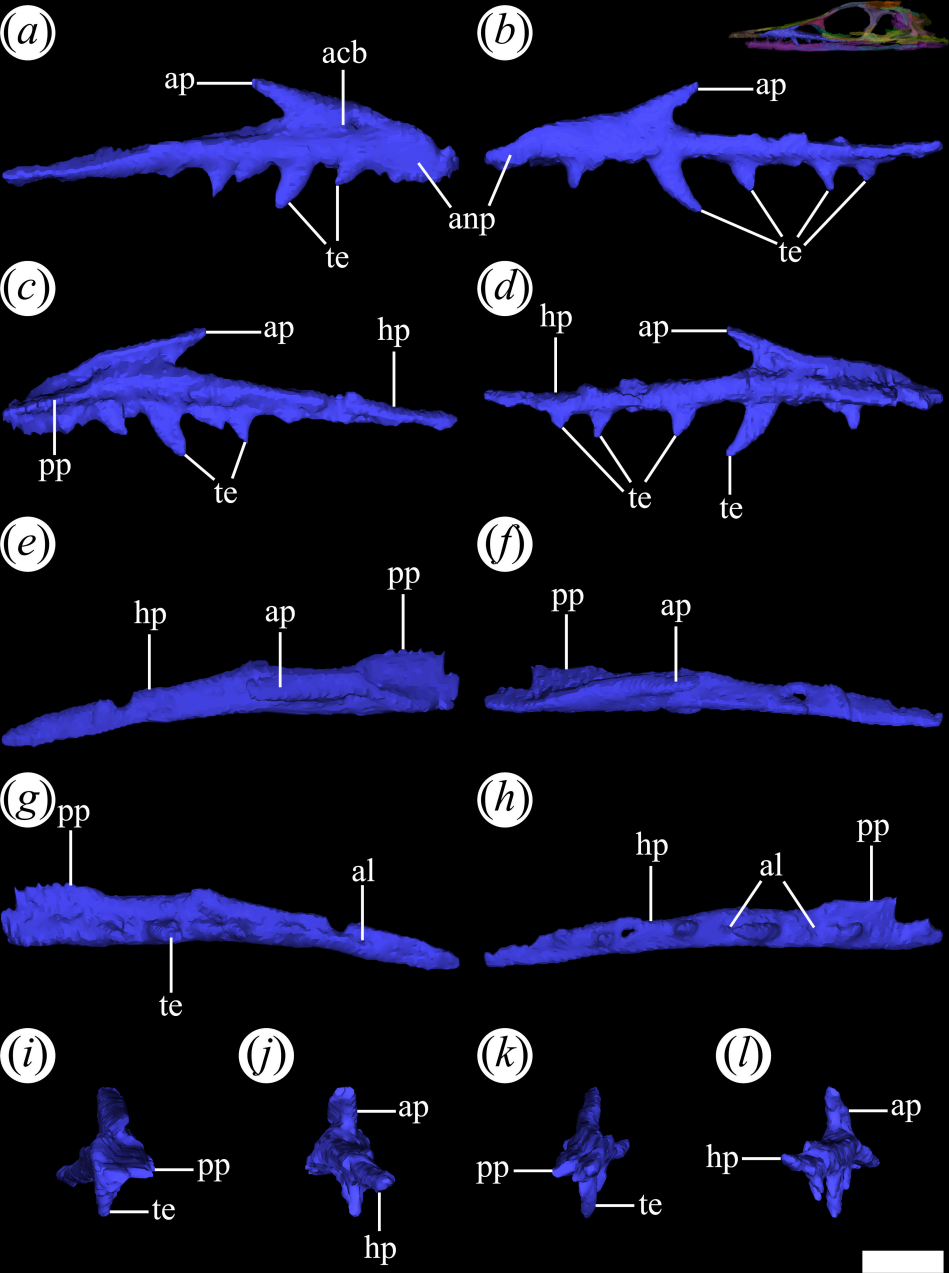
Digital reconstruction of the maxilla of *Tropidosuchus romeri* (PVL 4604) in (*a,b*) lateral view; (*c,d*) medial view; (*e,f*) dorsal view; (*g,h*) ventral view; (*i,k*) anterior view; and (*j,l*) posterior view. (*a,c,e,g,i,j*) Right maxilla. (*b,d,f,h,k,l*) Left maxilla. acb, artificial collapse; al, alveolus; anp, anterior process; ap, ascending process; hp, horizontal process; pp, palatal process; te, teeth. Scale bars equal 5 mm.

The horizontal process (figure 10*c–e*,*h, j,l*, hp) of the maxilla of *T. romeri* is long and reaches posteriorly the level of the mid-length of the orbit. By contrast, the horizontal process of the maxilla is considerably longer, extending to the level of the posterior edge of the orbit and eventually surpassing it, in *Ch. bonapartei* (PULR-V 07) and *Ps. ischigualastensis* (PVSJ 567). The horizontal process of *T. romeri* forms the entire ventral border of the antorbital fenestra, and it does not contribute to the external border of the orbit because of the presence of a jugal–lacrimal contact. However, the maxilla participates on the internal anteroventral edge of the orbit. The horizontal process extends posteriorly beneath the anterior process of the jugal along a suture with an anterodorsal-to-posteroventral orientation. The horizontal process lacks an antorbital fossa on its external surface, like in most proterochampsids to the exclusion of *Rh. gracilis* [37].

The anterior process (figure 10*a,b**,* anp) of the maxilla of *T. romeri* is subtriangular, with a tapering anterior end, and is considerably shorter than the horizontal process. There is no anterior maxillary foramen on its lateral surface. At the anterior apex of the antorbital fenestra and on the lateral surface of the maxilla there is a slightly depressed area in PVL 4604 and 4606. This depression is interpreted as an artificial collapse (figure 10*a*, acb) that affected that region of the bone and we interpret that the maxilla of *T. romeri* lacks an antorbital fossa. The palatal process projects medially as a ‘shelf-like’ structure on the anterior half of the internal surface of the anterior process (figure 9*c,e–i,k**,* pp). By contrast, the palatal process of the maxilla is considerably more posteriorly extended, occupying at least the anterior three-quarters of the anterior process, in *Proterochampsa barrionuevoi* and *Ch. bonapartei* [24,33]. The maxillary palatal process of *T. romeri* does not contact its counterpart, as in most non-archosaurian archosauriforms [31].

The ascending process of the maxilla is low and slopes posterodorsally at an angle slightly lower than 45° (figure 10*a–f*,*j,l**,* ap). This process forms the anterodorsal edge of the antorbital fenestra and contacts the prefrontal, as occurs in other proterochampsids, such as *Ps. ischigualastensis* (PVSJ 567: [23]) and *Gu. reigi* (PVL 4576: [24]).

The insufficient contrast in the first micro-CT of PVL 4604 made it difficult to determine the exact number of tooth positions on the maxilla. However, some alveoli could be recognized (figure 10*g,h**,* al). In the second scan of the same specimen, 11 alveoli are counted (figure 9*a*), with some teeth preserved *in situ*. This low number of alveoli is similar to that observed in the maxilla of *Proterochampsa barrionuevoi* (12 positions: MACN-Pv 18165) and *Proterochampsa nodosa* (11 positions: [42]). PVL 4606 is the specimen with better preserved maxillary teeth and considering the gaps between them, at least 13 maxillary tooth positions are estimated for *T. romeri*. A similar maxillary tooth count of 14−15 positions occurs in *Ch. bonapartei*, *Gu. reigi* and *Pi. rodriguesi* [16,38]. The maxillary tooth count of *Ce. binsfeldi* is more uncertain, with a probable range of 10−14 tooth positions (MAP-0657). All the preserved teeth of *T. romeri* have a similar shape, but vary in size (figure 10*a–d*,*i,k*, te). They are labiolingually compressed and recurved, without evidence of denticles. The maxillary dentition of *T. romeri* is similar to that of most Proterochampsidae, but differs from non-proterochampsid proterochampsians, including the straight teeth of *L. somnii* [61], the conical crowns with longitudinal ridges of *Ru. sixmilensis* [62] and the heterodont dentition of *Vancleavea campi* [63].

### 4.4. Nasal

**Figure 11.**
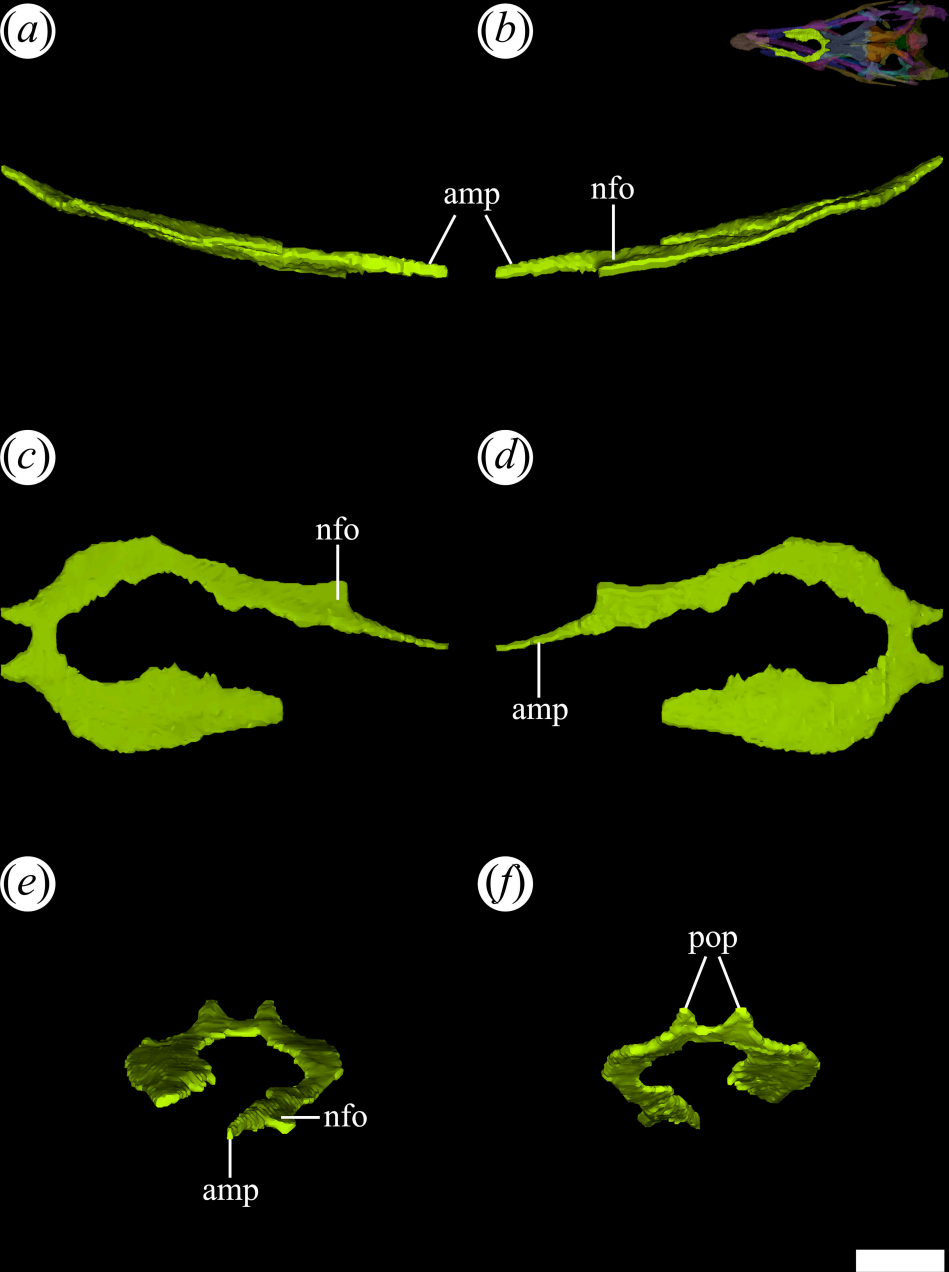
Digital reconstruction of the right and left nasal of *Tropidosuchus romeri* (PVL 4604) in (*a*) right lateral view; (*b*) left lateral view; (*c*) dorsal view; (*d*) ventral view; (*e*) anterior view; and (*f*) posterior view. amp, anteromedial process; nfo, narial fossa; pop, posterior process. Scale bars equal 5 mm.

PVL 4601 and 4606 preserve mostly intact nasals, while substantial damage is present in the medial region of both nasals of PVL 4604. The nasals are the most extensive bones of the dorsal surface of the rostrum. They are paired bones that articulate with each other through a median, straight suture, which is more clearly seen in PVL 4606. They are 1.3 times longer anteroposteriorly than the frontals. By contrast, this ratio is larger in other species of Proterochampsidae [36]. In particular, the nasals are twice or more than twice the length of the frontals in *Ce. binsfeldi* (2.0: MAP-657), *Ps. ischigualastensis* (2.1: PVSJ 567) and *Ch. bonapartei* (2.3: PULR-V 07). Both nasals extend from the posterior edge of the external naris to contact with the frontals posteriorly. The suture of the nasals with the frontals is generally obliterated by ornamentation on the cranial roof. However, the micro-CT scan of PVL 4604 reveals that this suture is W-shaped, in which two posterior processes project from the nasals (figure 11*f**,* pop) and two anterolateral and a median process extend anteriorly from the frontals. This suture is also positioned at the level of the anterior edge of the orbits and is similar to that in other Proterochampsidae, but particularly reminiscent of that of *Ch. bonapartei* (PULR-V 07: [36]).

The external ornamentation of the nasal consists of a sharp crest that extends diagonally, from anterolateral to posteromedial. This ornamentation pattern is more clearly visible in PVL 4606 and contrasts with that of rhadinosuchines (*Ps. ischigualastensis*: PVSJ 567, *Ch. bonapartei*: PULR-V 07, *Gu. reigi*: PULR-V 05), where the crests project from a single median point and radiate outwards. Laterally, the nasals contact the maxillae and the prefrontals. The suture with the prefrontal is mostly straight, slanting anterolaterally to posteromedially in dorsal view. In PVL 4604, the right nasal is more complete than the left one and its contribution to the posterior edge of the external naris is preserved. Just posterior to the external naris, there is a shallow fossa (figure 11*b*,*c*,*e**,* nfo) with a smooth surface delimited by two prominent ridges converging posteriorly. This same fossa is also present in *Gu. reigi* (PULR-V 05), *Ch. bonapartei* (PULR-V 07), *Ps. ischigualastensis* (PVSJ 567), *Ce. binsfeldi* (MAP-657: [37]) and *Proterochampsa nodosa* (MCP 1694 PV: [37]). The long and thin anteromedial process (figure 11*a*,*b*,*d*,*e**,* amp) preserved in the right nasal articulates with the posteromedial process of the premaxilla via an extensive, straight suture parallel to the midline of the skull (PVL 4604). This anteromedial process also forms the posteromedial edge of the external naris, but in other species with a longer process, such as *Rh. gracilis* (BSPG AS XXV 50), *Ch. bonapartei* (MCZ 4037) and *Gu. reigi* (PULR-V 05), it forms more than half of the medial edge of the opening [37]. The internal surface of the nasal is flat and smooth.

### 4.5. Lacrimal

**Figure 12.**
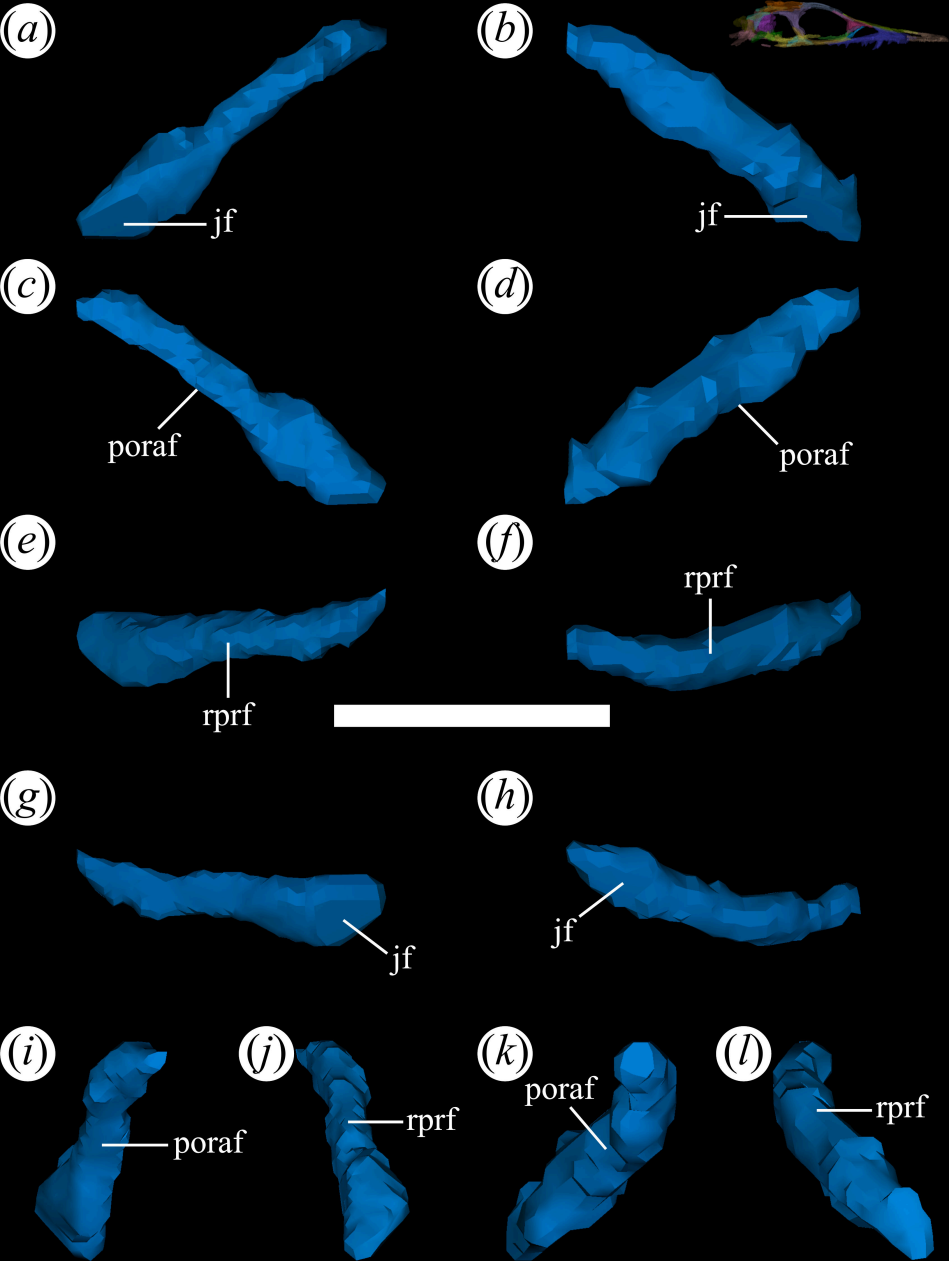
Digital reconstruction of the lacrimal of *Tropidosuchus romeri* (PVL 4604) in (*a,b*) lateral view; (*c,d*) medial view; (*e,f*) dorsal view; (*g,h*) ventral view; (*i,k*) anterior view; and (*j,l*) posterior view. (*a,c,e,g,i,j*) Right lacrimal. (*b,d,f,h,k,l*) Left lacrimal. jf, jugal facet; poraf, posterior rim of the antorbital fenestra; rprf, rim prefrontal. Scale bars equal 5 mm.

The lacrimal is relatively well-preserved in all specimens of *T. romeri* with fairly complete skulls (PVL 4601, 4604, 4606; [25]), forming the posterior and posterodorsal edges of the antorbital fenestra (figure 12*c*,*d*, *i*,*k**,* poraf). The ventral end of the bone also has a small contribution to the anteroventral edge of the orbit, but this contribution is greater in PVL 4606 than in other specimens of the species. The lacrimal departs from the typical inverted ‘L’-shaped bone, with distinct anterior and ventral processes, present in rhadinosuchines [12]. Originally, it was interpreted that the lacrimal of *T. romeri* was a simple vertical bar [25]. This interpretation matches with what is rendered from the micro-CT scan of PVL 4604, which reveals that it is indeed a bar, but with an anterodorsal inclination of approximately 45° (figure 12). The suture with the prefrontal extends in an anterodorsal to posteroventral direction and is only exposed in lateral view (figure 12*e*,*f*, *j*,*l**,* rprf). Additionally, at the posteroventral corner of the antorbital fenestra, the lacrimal contacts the tip of the anterior process of the jugal on a small, anteroposteriorly oriented, medial articular surface (figure 12*a*,*b*,*g*,*h**,* jf). Also, the posteroventral end of the lacrimal has minimal contact with the dorsal surface of the horizontal process of the maxilla. On the dorsal edge of the antorbital fenestra, the long and wide anterior process of the prefrontal prevents the contact of the lacrimal with the nasal. Similar to *Ps. ischigualastensis* (PVSJ 567) and the genus *Proterochampsa* [33,42], the lacrimal of *T. romeri* lacks an antorbital fossa, contrasting with the presence of such fossa in *Ce. binsfeldi* [37], *Ch. bonapartei* (PULR-V 07), *Gu. reigi* (PULR-V 05), *Rh. gracilis* [37], *Pi. rodriguesi* [16] and the indeterminate rhadinosuchine CRILAR-Pv 491 [38].

### 4.6. Jugal

**Figure 13.**
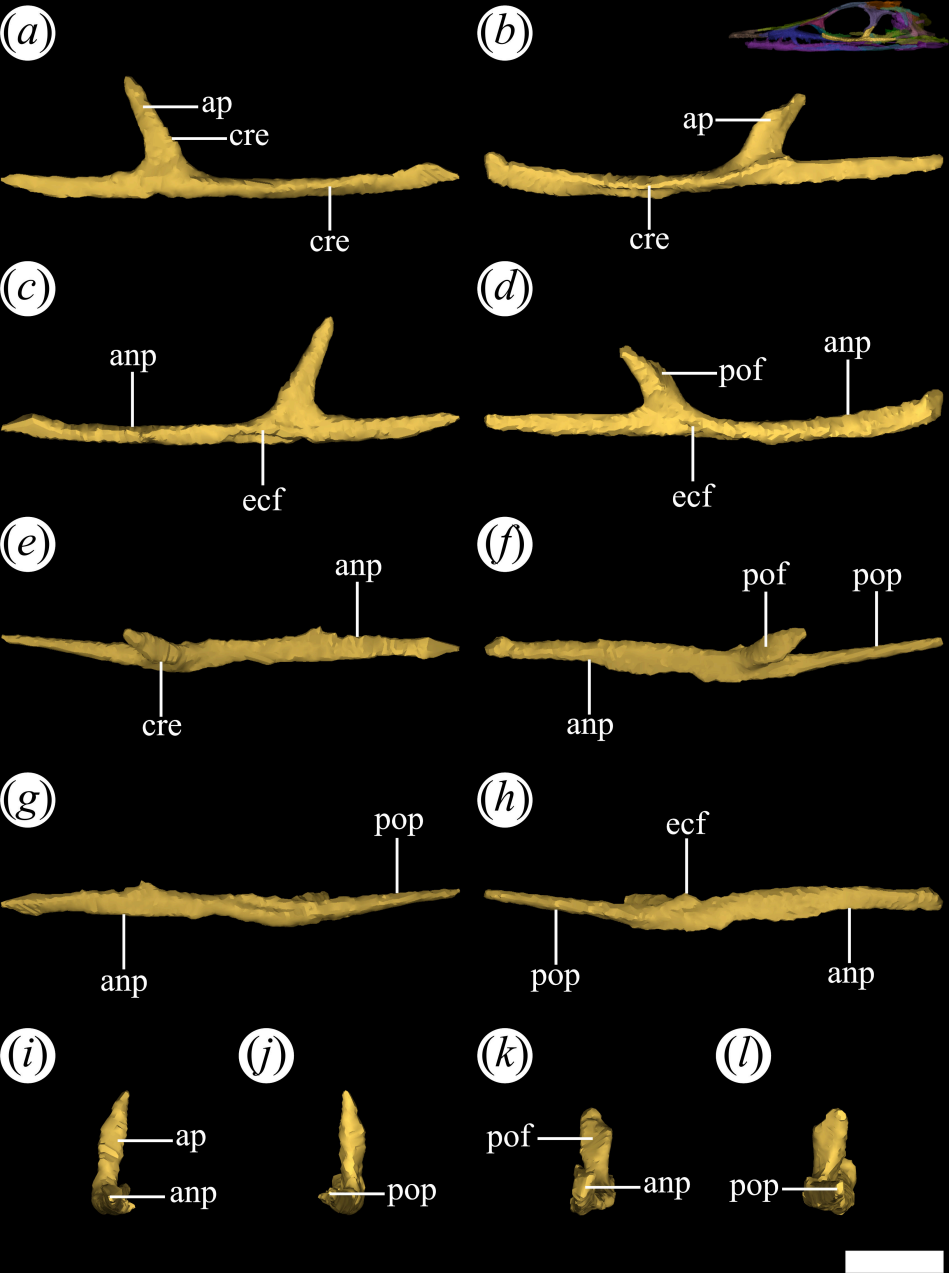
Digital reconstruction of the jugal of *Tropidosuchus romeri* (PVL 4604) in (*a,b*) lateral view; (*c,d*) medial view; (*e,f*) dorsal view; (*g,h*) ventral view; (*i,k*) anterior view; and (*j,l*) posterior view. (*a,c,e,g,i,j*) Right jugal. (*b,d,f,h,k,l*) Left jugal. anp, anterior process; ap, ascending process; cre, crest; ecf, ectopterygoid facet; pof, postorbital facet; pop, postorbital process. Scale bars equal 5 mm.

The jugal is a very thin bone, elongated anteroposteriorly and low dorsoventrally. In PVL 4601, 4604 and 4606, the jugal is relatively well-preserved. The characteristic triradiate shape of the archosauromorph jugal is a result of the development of three distinct processes [15]. The anterior process (figure 13*c*–*h,i,k**,* anp) is the longest in *T. romeri* and extends dorsally to the horizontal process of the maxilla. The diagonal suture of the jugal with the maxilla has an anterodorsal to posteroventral orientation. The extreme dorsoventral thinness of the anterior process is constant from its base to the anterior end, similar to *Ch. bonapartei* although its anterior process is shorter. In other Proterochampsidae, such as *Ce. binsfeldi*, *Gu. reigi* (PULR-V 05: [24]) and *Ps. ischigualastensis* (PVSJ 567: [23]), the anterior process of the jugal is shorter anteroposteriorly and becomes taller dorsoventrally. By contrast, in *Pi. rodriguesi* [16] and both species of *Proterochampsa* [33,42], the anterior process is much thicker because the jugal is a more robust bone. The ventral edge of the orbit is mostly formed by the anterior process of the jugal, a common feature in most rhadinosuchines (*Ps. ischigualastensis*: PVSJ 567, *Ch. bonapartei*: PULR-V 07, *Gu. reigi*: PULR-V 05). The most anterior end of this process contacts the lacrimal and has a limited contribution to the posteroventral corner of the antorbital fenestra, as seen in *Ps. ischigualastensis* (PVSJ 567: [23]), CRILAR-Pv 491 (an indeterminate rhadinosuchine from Chañares; [38]), and also in the holotypes of *Ch. bonapartei* (PULR-V 07; [36]) and *Gu. reigi* (PULR-V 05). By contrast, the anterior process of the jugal is so extensive that it forms most of the lateral edge of the antorbital fenestra in the two species of *Proterochampsa* [33,42]. Regarding *Pi. rodriguesi*, the contribution of the jugal to the antorbital fenestra is not clear in any of the three available skulls [16]. A well-developed anteroposterior crest extends along the lateral surface of the anterior process to the anteroventral corner of the infratemporal fenestra (figure 13*a*,*b**,* cre). In some rhadinosuchines, this crest is thicker and also extends onto the posterior process, as in *Ps. ischigualastensis* (PVSJ 567) and some specimens of *Ch. bonapartei* (MCZ 4039, PVL 4586: [16]). A similar crest has also been reported in *Proterochampsa barrionuevoi* [33], although it is unknown in *Proterochampsa nodosa* [42].

The ascending process of the jugal is very short dorsoventrally and slightly inclined posteriorly in lateral view (figure 13*a*,*b*,*i**,* ap). In this view, the anterior edge of the ascending process of the left jugal of PVL 4604 is sigmoid, while the posterior edge is straight. This contrasts with the edges of the ascending process of the right jugal that are both straight and converge at its dorsal end. As a result, the ascending process of the left jugal would have a different shape than its counterpart, as seen in the reconstructed model and the actual material. The ascending process has a medial facet that receives the ventral process of the postorbital (figure 13*d*,*f*,*k**,* pof). The contact between the left jugal and postorbital of PVL 4604 exposes a somewhat more inclined suture than the right side, but both possess the same anteroventral to posterodorsal orientation. In addition, the left suture is considerably sigmoid, while the right side is straight throughout its extension. The suture shape between the jugal and postorbital may vary between proterochampsian species and specimens. For example, Trotteyn & Ezcurra [36] identified an inverted V-shaped suture on the left side of the skull of the holotype of *Ch. bonapartei* (PULR-V 07). A similar situation occurs in *V. campi* [63], while the suture between the jugal and postorbital in *Proterochampsa nodosa* adopts a U-shaped morphology [42].

In PVL 4606, the ascending process of the jugal is excluded from the posterior border of the orbit, as in *Ps. ischigualastensis* (PVSJ 567), *Gu. reigi* (PULR-V 05, PVL 4576), *Ch. bonapartei* (PULR-V 07) and both species of *Proterochampsa* (MCZ 3408: [33], MCP 1694-PV: [42]). However, in PVL 4604, the jugal participates on the ventral region of the posterior border of the orbit, similar to *Ce. binsfeldi* (MAP-657), although in the latter species, its contribution is more reduced [16]. The ascending process has a poorly developed ridge (figure 13*a*,*e*, cre) extending along its anterior margin. This ridge does not contact ventrally with the crest on the lateral surface of the anterior process of the jugal, a condition that *T. romeri* shares with *Ps. ischigualastensis* (PVSJ 567: [23]), *Ch. bonapartei* (PULR-V 07, PVL 4586, MCZ 4039: [38]) and *Gu. reigi* (PULR-V 05, PVL 4576).

The posterior process of the jugal is straight, thin, and tapers at its posterior end, where it contacts the quadratojugal (figure 13*f*–*h,j,l**,* pop). Compared with the anterior process, it is equally thin but shorter anteroposteriorly, forming only the anterior half of the ventral edge of the infratemporal fenestra. The micro-CT of PVL 4604 reveals that the contact between the jugal and the quadratojugal is not preserved. Possibly, this is owing to the removal of both elements from their natural position as a result of preservation. However, in the ventral bar of the infratemporal fenestra of PVL 4601 (holotype) and PVL 4606, it is possible to see how the contact between the jugal and quadratojugal externally exposes a straight suture, displaced posteriorly and inclined with an anteroventral to posterodorsal orientation.

### 4.7. Prefrontal

**Figure 14.**
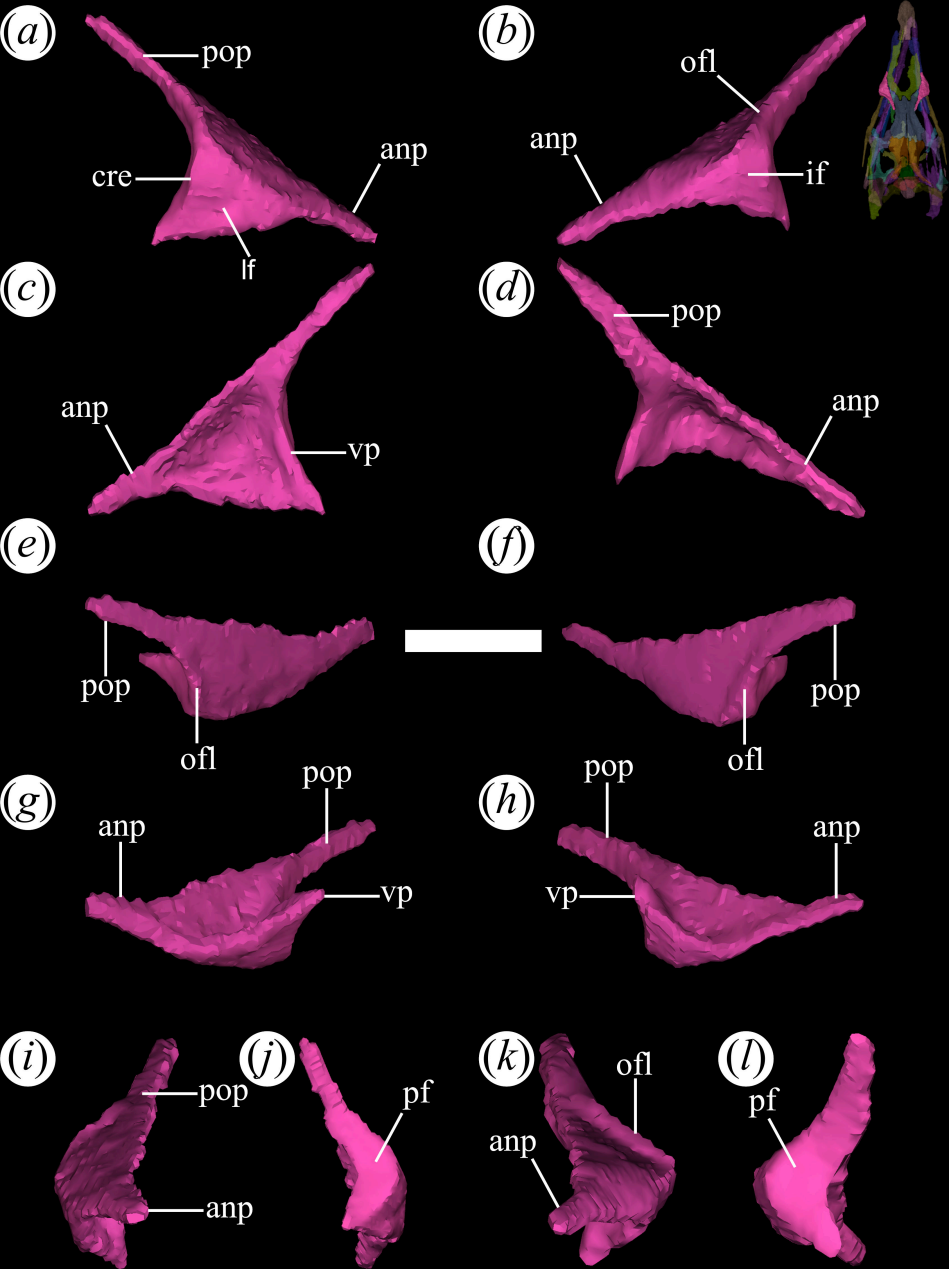
Digital reconstruction of the prefrontal of *Tropidosuchus romeri* (PVL 4604) in (*a,b*) lateral view; (*c,d*) medial view; (*e,f*) dorsal view; (*g,h*) ventral view; (*i,k*) anterior view; and (*j,l*) posterior view. (*a,c,e,g,i,j*) Right prefrontal. (*b,d,f,h,k,l*) Left prefrontal. anp, anterior process; cre, crest; lf, lateral face; ofl, orbital flange; pf, posterior face; pop, posterior process; vp, ventral process. Scale bars equal 5 mm.

The prefrontal is subtriangular in dorsal view, with a distinct lateral expansion, as seen in various rhadinosuchines (*Ps. ischigualastensis*: PVSJ 567; *Gu. reigi*: PULR-V 05, PVL 4576; *Ch. bonapartei*: PULR-V 07). The prefrontal possesses transversely narrow and long anterior and posterior processes. The dorsal surface is entirely flat and lacks ornamentation. In posterolateral view, a ventral process extends from the prefrontal and gradually tapers at its lower apex (figure 14*c*, *g*,*h**,* vp). The anterior process of the prefrontal is slightly shorter than the posterior one. The anterior process articulates with the ascending process of the maxilla, forming the dorsal margin of the antorbital fenestra and excluding the nasal from this opening (figure 14*a–d,g–i,k**,* anp). The posterior process extends posterodorsally, contacts medially the lateral edge of the frontal, and forms the anterodorsal margin of the orbit (figure 14*a,d–i**,* pop). A very low flange is restricted to the anterodorsal border of the orbit (figure 14*b e*,*f*,*k**,* ofl). The ventral process presents a discreetly developed crest that subdivides the external surface of this process into two sides, one oriented laterally and the other posteriorly (figure 14*a**,* cre). This morphology of the external surface of the ventral process resembles that in *Ch. bonapartei* (MCZ 4039: [38]) and some undetermined rhadinosuchines from the Chañares Formation (CRILAR-Pv 491: [38]). As a consequence, in front of the orbit, the ventral process of the prefrontal possesses a transversely broad posterior surface (figure 14*j*,*l**,* pf), as well as a lateral surface (figure 14*a*,*b**,* lf) that contacts the lacrimal through an anterodorsally-to-posteroventrally oriented suture.

### 4.8. Frontal

**Figure 15.**
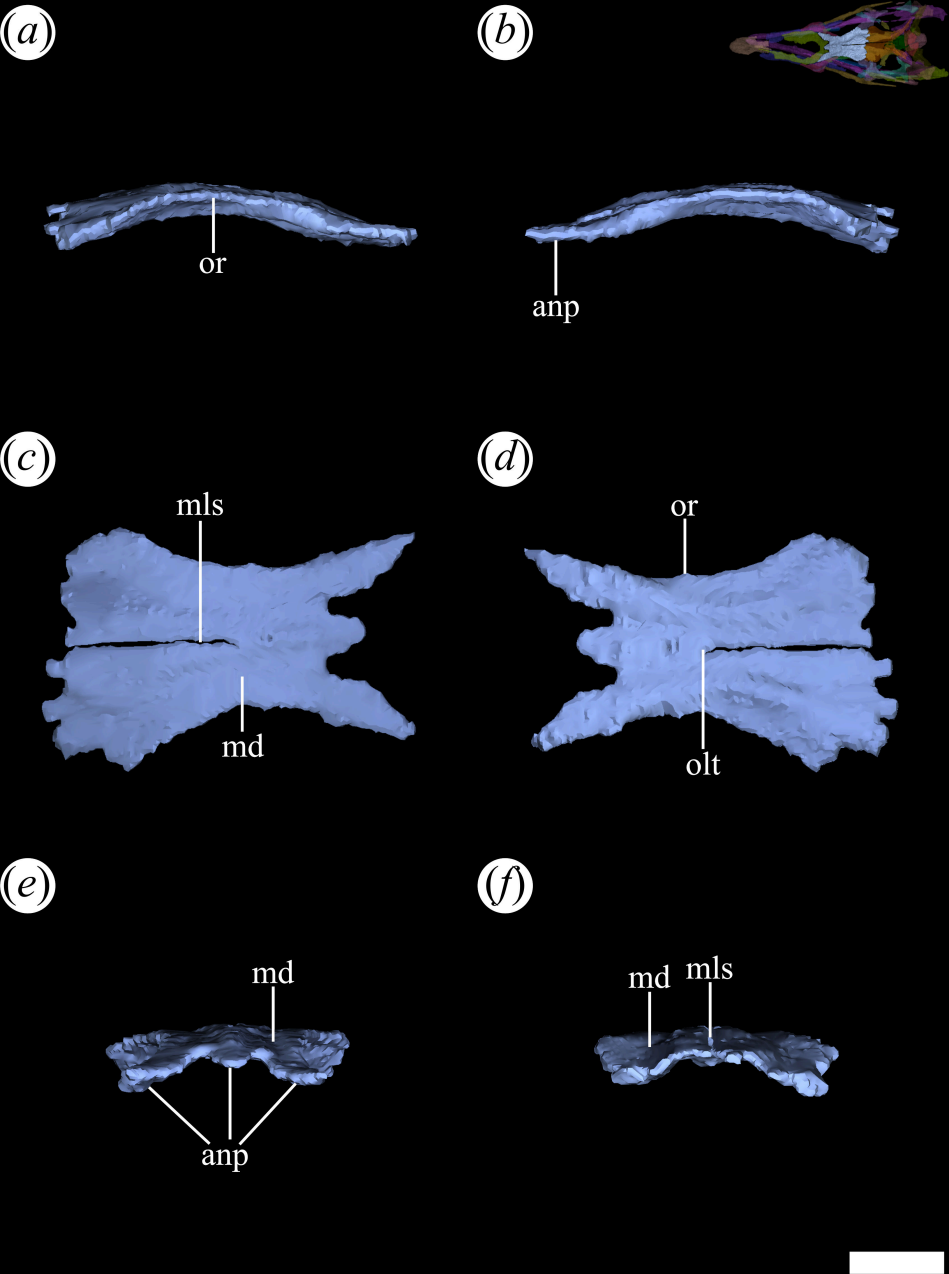
Digital reconstruction of the right and left frontal of *Tropidosuchus romeri* (PVL 4604) in (*a*) right lateral view; (*b*) left lateral view; (*c*) dorsal view; (*d*) ventral view; (*e*) anterior view; and (*f*) posterior view. anp, anterior process; md, medial depression; mls, midline suture; olt, olfactory tract; or, orbital rim. Scale bars equal 5 mm.

The frontal occupies the interorbital region of the skull roof and is a paired element with a suture extending along the midline. In many cases, this suture is not evident owing to the ornamentation present on the cranial roof, making it difficult to identify in PVL 4601, 4604 and 4606. However, the micro-CT data of PVL 4604 allow determination of the presence of such suture. It is straight and longitudinal, extending from the transversely narrowest region of the frontal bones to the contact with the parietals. Since this suture does not contact the nasals, the frontals of PVL 4604 seem to be partially fused to each other (figure 15*c*,*f**,* mls). The frontals are thin dorsoventrally, with an anteroposterior length nearly doubling their maximum transverse width, as seen in *Ch. bonapartei* (PULR-V 07) and *Proterochampsa nodosa* (MCP 1694-PV). By contrast, in *Ps. ischigualastensis* (PVSJ 567: [23]), the frontals are as long as transversely wide. The anterior end of the frontals articulates with the nasals anteriorly and the prefrontals laterally (figure 15*b*,*e**,* anp). Posteriorly, it makes contact with the parietals and posterolaterally with the postorbitals. The suture between frontals and parietals is straight and interdigitated, extending transversely to the main axis of the skull. This contrasts with the oblique orientation of the suture observed in *Proterochampsa nodosa* [42]. The articulation with the postorbitals is via a crenulated suture, following an anterolateral to posteromedial orientation in dorsal view, similar to *Ps. ischigualastensis* (PVSJ 567: [23]) and *Ch. bonapartei* (PULR-V 07: [36]).

Laterally, the frontals form part of the posterodorsal margin of the orbits (figure 15*a*,*d**,* or). This orbital margin is slightly raised, extending from the flange formed on the prefrontal, as seen in other proterochampsids (e.g. *Ps. ischigualastensis*: PVSJ 567; *Proterochampsa barrionuevoi*: PVSJ 77; *Ch. bonapartei*: PULR-V 07). The medial edge of the frontals is slightly elevated, forming a low ridge along the midline of the skull roof. In cross-section, the dorsal surface of each frontal possesses a shallowly concave depression (figure 15*c*, *e*,*f**,* md), also found in *Proterochampsa barrionuevoi* (PVSJ 77: [33]). On the internal surface of the frontals, the bony correlates of the tract and part of the olfactory bulbs are well developed (figure 15*d**,* olt). Externally, the pair of frontals of *T. romeri* [25] have an ornamentation that distinctly differs from that present in other proterochampsids. It consists of three ridges, one central and two lateral that depart from each other towards the posterior region of the bones. The arrangement of these ridges contrasts with the radial pattern observed in certain rhadinosuchines, such as *Ch. bonapartei* (PULR-V 07), *Ps. ischigualastensis* (PVSJ 567) and the holotype of *Gu. reigi* (PULR-V 05), as well as the distinctive nodular ornamentation typical of *Proterochampsa* [12,15].

### 4.9. Postfrontal

This bone is absent in the holotype and all referred specimens of *T. romeri* that preserve this region of the skull. This condition is shared with other proterochampsids [31].

### 4.10. Postorbital

**Figure 16.**
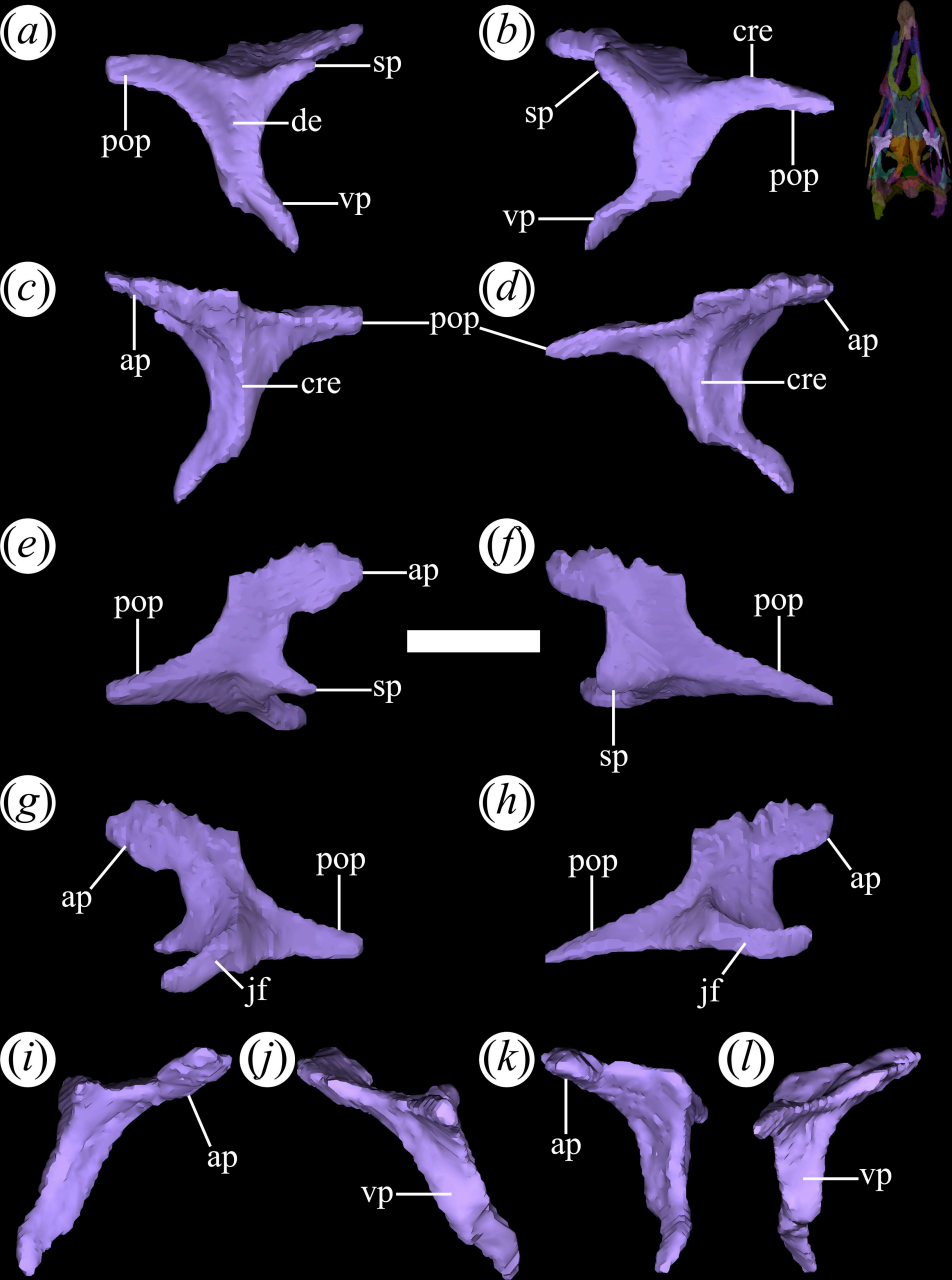
Digital reconstruction of the postorbital of *Tropidosuchus romeri* (PVL 4604) in (*a,b*) lateral view; (*c,d*) medial view; (*e,f*) dorsal view; (*g,h*) ventral view; (*i,k*) anterior view; and (*j,l*) posterior view. (*a,c,e,g,i,j*) Right postorbital. (*b,d,f,h,k,l*) Left postorbital. ap, ascending process; cre, crest; de, depression; jf, jugal facet; pop, postorbital process; sp, spur; vp, ventral process. Scale bars equal 5 mm.

The postorbital is a ‘Y’-shaped bone in dorsolateral view. It is located posterior to the orbits and contributes to the upper temporal bar. The ascending process (figure 16*c–e g–i,k**,* ap) of the postorbital projects anteromedially and its end has a double contact. The anterior half contacts the frontal, and the posterior half contacts the parietal. This condition is shared with other proterochampsids, such as *Ch. bonapartei* (PULR-V 07), *Ps. ischigualastensis* (PVSJ 567) and *Proterochampsa barrionuevoi* [33]. However, it differs from the condition in *Proterochampsa nodosa* (MCP 1694-PV: [42]), in which the postorbital only contacts the parietal. The articulation of the postorbital with the parietal forms a straight suture with an anteromedial to posterolateral orientation. The ascending process extends between the orbit and the supratemporal fenestra, forming the posterior and anterior edges of both openings, respectively. The ventral process contacts the jugal at its ventral end (figure 16*a*,*b*,*g*,*h*, *j*,*l**,* vp, jf). It lacks an anterior flexure that has been interpreted as an autapomorphy of *Pi. rodriguesi* [16]. This process primarily contributes to the posterior edge of the orbit but also forms the dorsal half of the anterior edge of the infratemporal fenestra in lateral view. In cross-section, the ventral process is ‘V’-shaped as a result of a gentle concavity on its lateral (‘external’) surface that extends dorsoventrally (figure 16*a**,* de). A strongly developed crest runs along the entire medial surface of this process and forms an internal separation between the orbit and the infratemporal fenestra (figure 16*c*,*d*, cre). There is no distinct process or scar for articulation with a laterosphenoid ossification (if this was present).

The posterior process of the postorbital is dorsoventrally thin, curves slightly ventrally, and extends between the supratemporal and infratemporal fenestrae (figure 16*a*–*h**,* pop). The upper temporal bar is formed by a subequal contribution of the postorbital and the squamosal. The medial surface of the posterior process of the postorbital contacts the lateral surface of the anterior process of the squamosal, forming a straight suture with a slight anteromedial to posterolateral orientation. This suture differs from the typical V-shaped suture present in other early archosauriforms [15]. A low crest rests on the dorsolateral edge of the posterior process, running along most of its length (figure 16*b**,* cre). On the anterior margin of the postorbital, at approximately the same height as the posterior process, a large, spur-like projection extends anteriorly into the orbit (figure 16*a*,*b*,*e*,*f**,* sp). This same structure, but proportionally lower, is also present in *Ps. ischigualastensis* (PVSJ 567), *Ch. bonapartei* (PULR-V 07), *Gu. reigi* (PULR-V 05, PVL 4576: [15]), and an indeterminate rhadinosuchine from the Chañares Formation (CRILAR-Pv 491: [38]).

### 4.11. Squamosal

**Figure 17.**
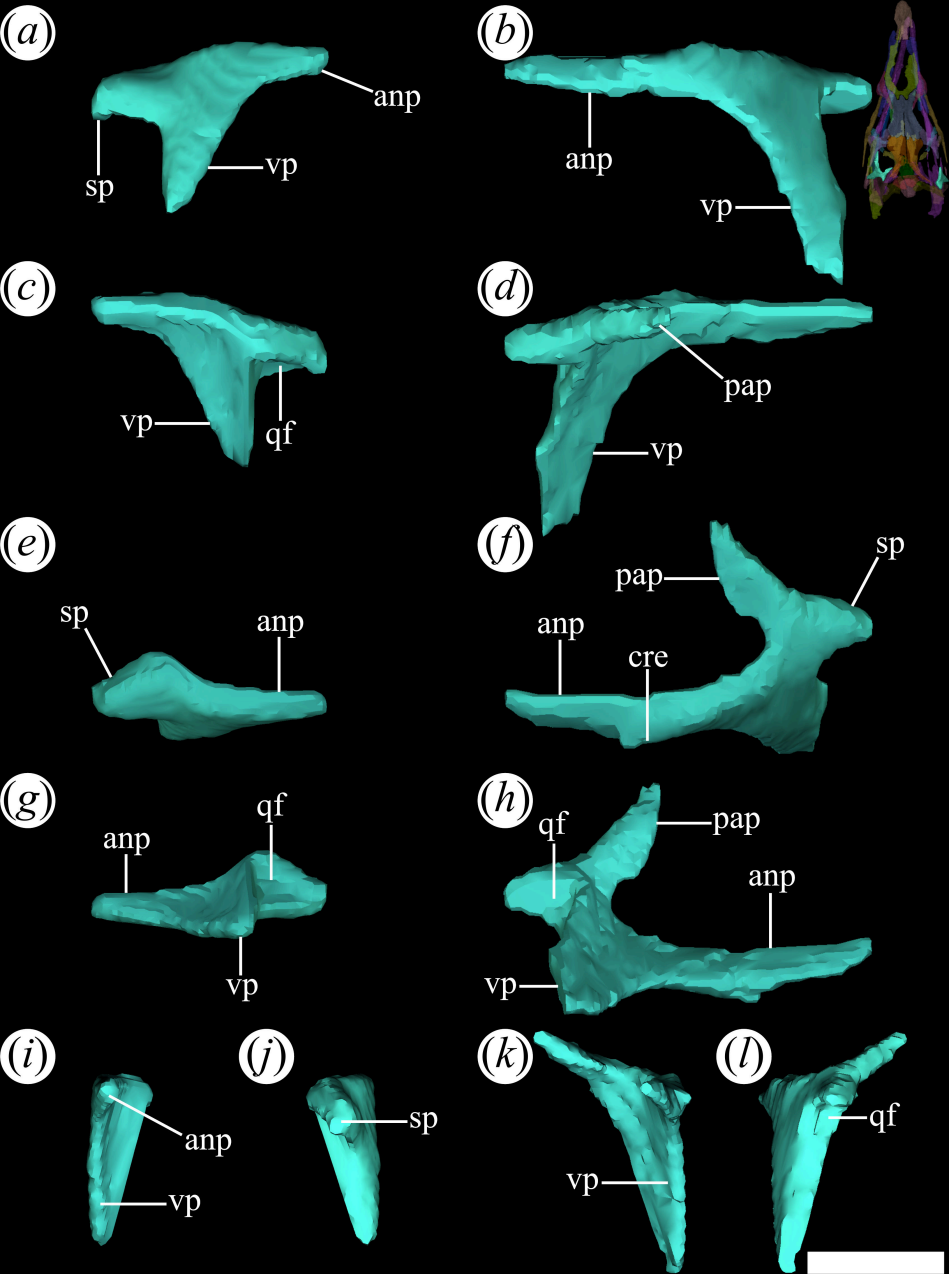
Digital reconstruction of the squamosal of *Tropidosuchus romeri* (PVL 4604) in (*a,b*) lateral view; (*c,d*) medial view; (*e,f*) dorsal view; (*g,h*) ventral view; (*i,k*) anterior view; and (*j,l*) posterior view. (*a,c,e,g,i,j*) Right squamosal. (*b,d,f,h,k,l*) Left squamosal. anp, anterior process; cre, crest; pap, parietal process; qf, quadrate facet; sp, spur; vp, ventral process. Scale bars equal 5 mm.

PVL 4604 has an incomplete right squamosal, lacking its medial process and with partially preserved anterior and ventral process. By contrast, the left squamosal of PVL 4604 is complete. The squamosal of *T. romeri* was originally described as a triradiate bone [22], but the micro-CT of PVL 4604 shows that it is a tetraradiate element. The squamosal forms the posterolateral corner of the supratemporal fenestra in dorsal view. The anterior process (figure 17*a,b,e–i*, anp) contacts the postorbital and is approximately as long as the posterior process of the postorbital. The anterior process of the squamosal is dorsoventrally narrow along its entire length, tapering at its anterior end, and possesses a well-marked crest running along its dorsal surface (figure 17*f*, cre). Additionally, it forms approximately the whole lateral edge of the supratemporal fenestra, similar to *Ce. binsfeldi* [51], *Ch. bonapartei* (PULR-V 07) and *Gu. reigi* (PVL 4576: [15]). However, in *Proterochampsa barrionuevoi* (MCZ 3408: [33]) and *Ps. ischigualastensis* (PVSJ 567), the anterior process of the squamosal is shorter and restricted to the posterior half of the lateral edge of the supratemporal fenestra.

The ventral process of the squamosal forms a slightly obtuse angle with the anterior process (figure 17*a–d,g–i,k*,vp). The angle formed by both processes is often slightly higher, as in *Ps. ischigualastensis* (PVSJ 567) and in some specimens of *Ch. bonapartei* (MCZ 4037, 4039: [38]), but the holotype of *Ch. bonapartei* (PULR-V 07) shares the same condition of *T. romeri*. The ventral process of the squamosal is relatively long, contributing to slightly more than half of the posterior edge of the infratemporal fenestra, resembling the condition in *Ps. ischigualastensis* (PVSJ 567), *Ce. binsfeldi* [51], *Gu. reigi* (PULR-V 05, PVL 4576), *Ch. bonapartei* (PULR-V 07) and *Proterochampsa barrionuevoi* (PVSJ 606, 77, PVL 2603). Most of the posterior edge of the ventral process of the squamosal contacts the quadrate, and its posteroventral corner contacts the dorsal process of the quadratojugal. The contact of the squamosal with the quadratojugal defines a straight, mainly dorsoventrally oriented suture. The posterior edge of the supratemporal fenestra is mostly formed by the medial (= parietal) process (figure 17*d*,*f*,*h**,* pap) of the squamosal, which medially contacts the posterolateral process of the parietal in a simple anteromedially-to-posterolaterally oriented suture. The posterior process of the squamosal (figure 17*a*,*b*,*e*,*j**,* sp) is considerably shorter than the other three processes of the bone and its ventral surface receives the head of the quadrate (figure 17*c*,*g*,*h*,*l**,* qf). This process does not extend beyond the posterior edge of the quadrate head, as seen in *Ps. ischigualastensis* (PVSJ 567), *Ce. binsfeldi* [51], *Proterochampsa barrionuevoi* (PVL 2063: [33]) and *V. campi* [63]. However, this condition differs from *Ch. bonapartei* (PULR-V 07) and *Gu. reigi* (PULR-V 05), in which the posterior process extends beyond the posterior edge of the quadrate head.

### 4.12. Quadratojugal

**Figure 18.**
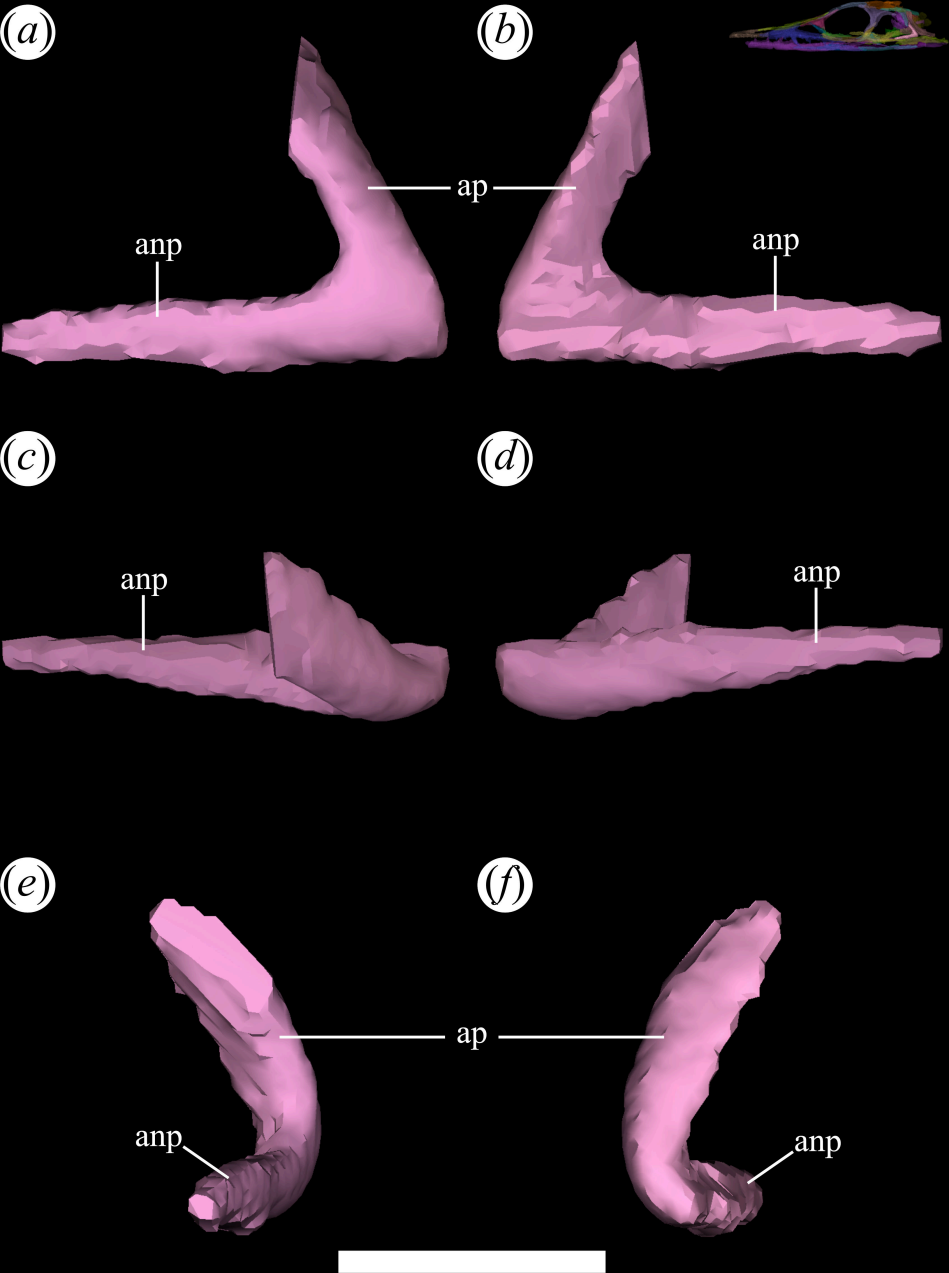
Digital reconstruction of the left quadratojugal of *Tropidosuchus romeri* (PVL 4604) in (*a*) right lateral view; (*b*) left lateral view; (*c*) dorsal view; (*d*) ventral view; (*e*) anterior view; and (*f*) posterior view. anp, anterior process; ap, ascending process. Scale bars equal 5 mm.

The quadratojugal forms the posteroventral corner of the infratemporal fenestra. It is an L-shaped bone with anterior and dorsal (= ascending) processes (figure 18*a*,*b*,*e*,*f**,* anp, ap). In the quadratojugal of PVL 4606, a third process becomes conspicuous, projecting in a posteroventral direction and, in lateral view, obliterating the lateral edge of the quadrate. However, this third process is diminished or absent in the quadratojugal of PVL 4604. In PVL 4606, the suture between the quadratojugal and the jugal is oriented anteroventrally to posterodorsally and positioned at mid-length in the lower temporal bar. As for the lower temporal bar, the suture covers approximately one-third of its length in the holotype of *Gu. reigi* (PULR-V 05). By contrast, in specimen PVL 4606 of *T. romeri*, its extension covers more than half, similar to that observed in *Ch. bonapartei* (PULR-V 07, PVL 4575, 4586), *Gu. reigi* (PVL 4576: [12]) and *Ps. ischigualastensis* (PVSJ 567: [23]). The anterior process of the quadratojugal is elongated anteroposteriorly and forms approximately the posterior half of the lower temporal bar (figure 18*a*–*f*, anp). In some early archosauriforms, the contribution of the quadratojugal to the lower border of the infratemporal fenestra is limited by the extreme development of the posterior process of the jugal, as seen in *Er. africanus* [64] and *Proterosuchus fergusi* [65]. The anterior process of the quadratojugal of *T. romeri* is slightly longer than the dorsal process. The lateral surface of the transition between the dorsal and anterior processes of the quadratojugal is smooth and slightly convex, lacking the infratemporal fossa present in various rhadinosuchines, such as *Rh. gracilis* (BSPG AS XXV 50: [37]), *Ch. bonapartei* (PULR-V 07: [36]) and *Gu. reigi* (PULR-V 05, PVL 4576: [15]). A similar situation is also observed in *Ce. binsfeldi* (MAP-657: [19]), *Proterochampsa nodosa* (MCP 1694-PV: [42]), and *Pi. rodriguesi* (UFRGS-PV-0464-T: [16]). Laterally, the dorsal process of the quadratojugal contacts the squamosal and forms most of the ventral half of the posterior edge of the infratemporal fenestra (figure 18*a*,*b*,*e*,*f**,* ap). Additionally, it has a posteromedial contact with the quadrate that is exposed as an anterodorsally-to-posteroventrally oriented suture in posterolateral view. Dorsally, the continuity of this suture is observed in the contact of the squamosal with the quadrate, while in its ventral extension, it is interrupted by the quadrate foramen, which opens posteriorly. This small subcircular opening, circumscribed laterally by the quadratojugal and medially by the quadrate, is clearly discernible in PVL 4601 (holotype) and PVL 4606. By contrast, this opening is not completely preserved in PVL 4604 because of the displacement of the quadratojugal with respect to the quadrate.

### 4.13. Parietal

**Figure 19.**
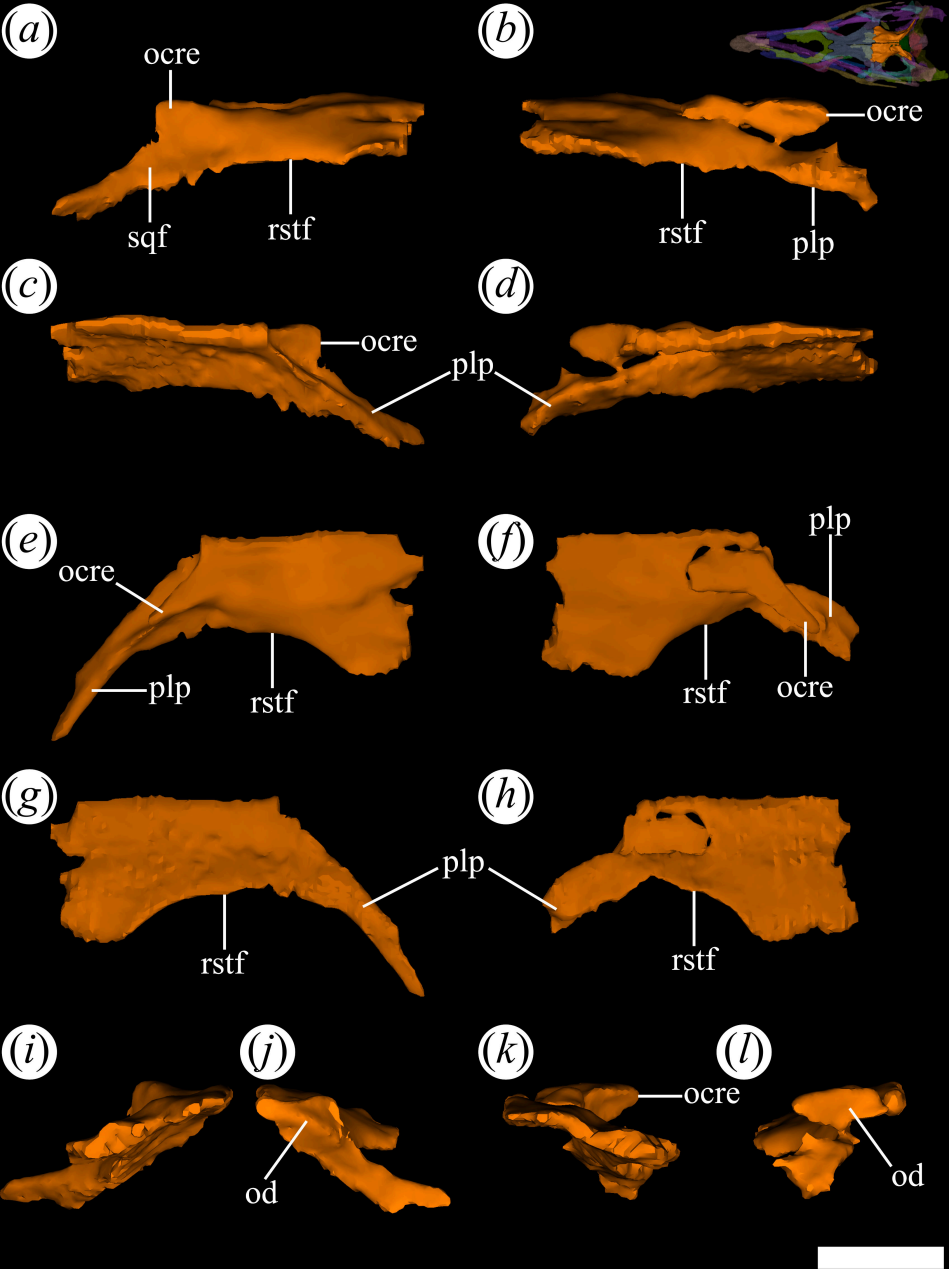
Digital reconstruction of the parietal of *Tropidosuchus romeri* (PVL 4604) in (*a,b*) lateral view; (*c,d*) medial view; (*e,f*) dorsal view; (*g,h*) ventral view; (*i,k*) anterior view; and (*j,l*) posterior view. (*a,c,e,g,i,j*) Right parietal. (*b,d,f,h,k,l*) Left parietal. ocre, occipital crest; od, occipital depression; plp, posterolateral process; rstf, rim supratemporal fenestra; sqf, squamosal facet. Scale bars equal 5 mm.

This element forms the posterior region of the skull roof and, with its counterpart, separates the supratemporal fenestrae from each other. Unlike the frontals, the suture between parietals extends completely along the midline from the posterior edge of the frontals to the dorsal edge of the occipital plate. This condition contrasts with what is observed in *Proterochampsa nodosa* [42] and some specimens of *Proterochampsa barrionuevoi* (PVL 2063, PVSJ 77, MCZ 3408 and MACN 18165: [33]) since the parietals lack this medial suture. In cross-section, the parietals are dorsally convex and have a thickness similar to the frontals. The parietals are elongated anteroposteriorly and narrow transversely, approximately 30% shorter than the frontal. The parietals form the entire concave medial margin of the supratemporal fenestrae (figure 19*a,b,e–h*, rstf). The dorsal surface of the parietals is ornamented by a median crest and two collateral crests aligned to the major axis of the bone. It is important to note that these three crests are an extension of those found on the dorsal surface of the frontals. There are no additional subsidiary crests, contrasting with the ornamentation organized in a radial pattern, present in *Ch. bonapartei* (PULR-V 07: [36]), *Ps. ischigualastensis* (PVSJ 567: [23]) and *Gu. reigi* (PVL 4576). The collateral crests connect posteriorly with well-developed occipital crests (figure 19*a*–*c,e, f,k**,* ocre) at the posterior edge of the parietals. In the holotype (PVL 4601), it can be observed that the occipital crests align with the anteroposterior axis of the parietals, while in PVL 4604 and 4606, they possess a slightly anteromedial-to-posterolateral orientation, similar to *Gu. reigi* (PVL 4576: [24]), and *Ch. bonapartei* (PULR-V 07: [36]). These occipital crests are located dorsally at the base of the posterolateral processes of the parietals (figure 19*b*–*h**,* plp).

The parietals contact the frontals anteriorly in a transverse suture and the postorbitals anterolaterally via a straight anteromedially-to-posterolaterally oriented suture. In addition, the absence of supratemporal fossae is a condition shared with *Ce. binsfeldi* (MAP-657: [12]) and both species of *Proterochampsa*. By contrast, the presence of such fossae occurs in *Ch. bonapartei* (PULR-V 07: [36]), *Gu. reigi* (PVL 4576: [15,24]) and *Pi. rodriguesi* [16]. In PVL 4604, the right posterolateral process is more complete than the left one, in which the latter only preserves its proximal half. The posterolateral processes diverge from each other along the posterior edge of the skull roof, forming an approximate right angle between them, as occurs in *Ch. bonapartei* (PULR-V 07: [36]), and *Ps. ischigualastensis* (PVSJ 567: [23]). By contrast, in both species of *Proterochampsa*, as well as in *Ce. binsfeldi* (MAP-657: [12]), this angle is more obtuse owing to a broader separation between the processes in dorsal view. Additionally, a shallow depression extends along the occipital surface (figure 19*j*,*l**,* od) of the posterolateral processes in posterior view. The posterolateral process articulates laterally with the squamosal and posteriorly with the otoccipital and supraoccipital.

### 4.14. Quadrate

**Figure 20.**
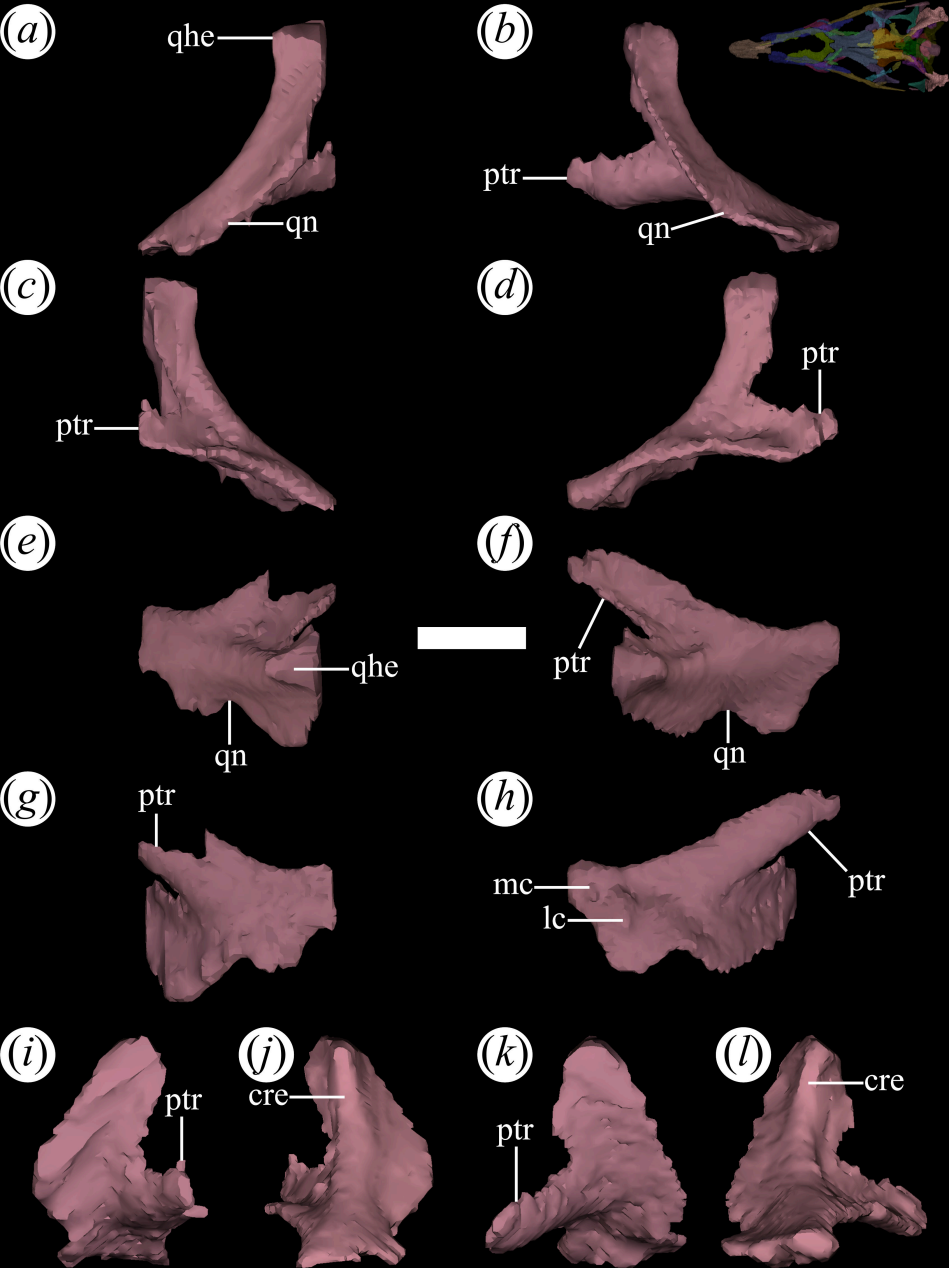
Digital reconstruction of the quadrate of *Tropidosuchus romeri* (PVL 4604) in (*a,b*) lateral view; (*c,d*) medial view; (*e,f*) dorsal view; (*g,h*) ventral view; (*i,k*) anterior view; and (*j,l*) posterior view. (*a,c,e,g,i,j*) Right quadrate. (*b,d,f,h,k,l*) Left quadrate. cre, crest; lc, lateral condyle; mc, medial condyle; ptr, pterygoid ramus; qhe, quadrate head; qn, quadrate notch. Scale bars equal 5 mm.

The quadrate occupies the posterolateral edge of the skull. The rendering of PVL 4604 reveals that the pterygoid ramus of the right quadrate is severely damaged, while it is more complete in the left quadrate (figure 20*b–d,g–i,k*, ptr). The quadrate is crescent-shaped in lateral view, with its anterior side convex and the posterior one concave. The quadrate possesses a strong anterodorsally-to-posteroventrally oriented slope in lateral view, which resembles the condition in most proterochampsid specimens [12] to the exclusion of the holotype of *T. romeri* (PVL 4601), in which this bone is more vertical [25]. However, this condition seems to be a taphonomic artefact. The anterior surface of the quadrate is transversely concave, while the posterior surface is convex. A crest extends along the posterior surface (figure 20*j*,*l**,* cre) and separates a posterolateral side from a posteromedial one, resembling the condition in *Proterochampsa barrionuevoi* (MCZ 3408: [33]) and *Ch. bonapartei* [36]. The head of the quadrate articulates (figure 20*a*,*e**,* qhe) on a shallow concavity on the ventral surface to the posterior process of the squamosal. The skull of PVL 4606 has a weak contact between the quadrate and the anterior surface of the paraoccipital process of the opisthotic, as also seen in *Ps. ischigualastensis* (PVSJ 567: [23]), *Ch. bonapartei* (PULR-V 07: [36]) and *Pi. rodriguesi* (UFRGS-PV-0464-T: [16]).

In PVL 4604, the quadrate has been digitally isolated, exposing two prominent condyles separated by an anteroposteriorly oriented groove on the ventral surface of the bone (figure 20*h**,* mc, lc). The condyles were identified only in the left quadrate, while their presence in the right quadrate could not be determined owing to the poor contrast/resolution of the micro-CT. The inclination of the quadrate is the cause that the ventral condyles are more posteriorly positioned than the posterior edge of the occipital condyle, as also occurs in *Ch. bonapartei* (PULR-V 07: [36]), *Gu. reigi* (PVL 457), *Proterochampsa barrionuevoi* (PVSJ 77: [33]) and *Proterochampsa nodosa* (MCP 1694-PV: [42]). The lateral and medial condyles of the quadrate are subequally developed and rounded. It should be noted that when the quadrate is articulated with the rest of the skull, the lateral condyle is more posteriorly positioned than the medial condyle. Evidence supporting the presence of a quadrate foramen in PVL 4604 is a notch on the lateral edge of the ventral half of the quadrate, representing the medial, dorsal and ventral edges of that opening (figure 20*a*,*b*, *e*,*f**,* qn). The pterygoid branch of the quadrate extends anteromedially, articulating with the pterygoid at its anterior end. The pterygoid ramus is a lamellar process with a broadly concave medial surface and a convex lateral side.

### 4.15. Palate

Some of the bones that form this region of the skull of *T. romeri* [25] are partially exposed in PVL 4601 (holotype) and PVL 4604 in ventral view. The palate of PVL 4606 is completely covered by matrix. Although the palate of specimen PVL 4604 could be reconstructed, almost in its entirety, and then virtually removed, the overall interpretation of its morphology and the contacts between the different bones was subject to the contrast of the micro-CT images.

### 4.16. Vomer

A partially exposed, anteroposteriorly elongated bone occurs slightly laterally to the median line of the palate and at the level of the antorbital fenestra in PVL 4606. Its morphology and position suggest that this bone probably represents a left vomer. However, no further information can be provided. The preservation of the palate in PVL 4601 and 4604 precludes discerning with certainty the presence or absence of a vomer. In addition, the set of micro-CT images, including the anterior region of the palate, shows unfavourable contrast for a virtual reconstruction of the vomer.

### 4.17. Palatine

**Figure 21.**
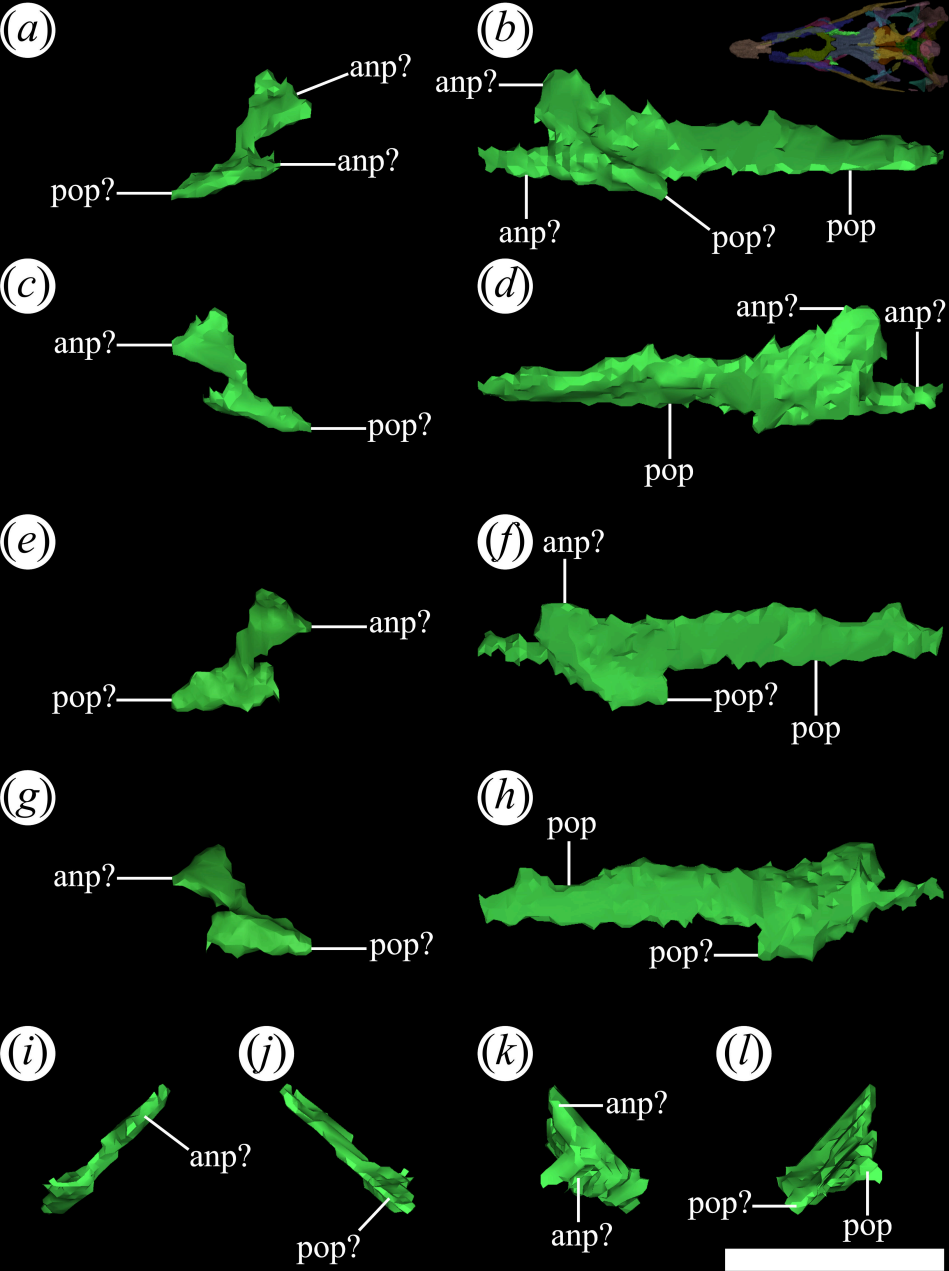
Digital reconstruction of the palatine of *Tropidosuchus romeri* (PVL 4604) in (*a,b*) lateral view; (*c,d*) medial view; (*e,f*) dorsal view; (*g,h*) ventral view; (*i,k*) anterior view; and (*j,l*) posterior view. (*a,c,e,g,i,j*) Right palatine. (*b,d,f,h,k,l*) Left palatine. anp?, anterior process; pop, posterior process. Scale bars equal 5 mm.

In the holotype specimen of *T. romeri* (PVL 4601: [25]), the palatines are partially covered by the lower jaw. Despite this, a larger portion of the ventral surface of the right palatine is exposed and in a relatively good state of preservation. Regarding the other specimens, only in the palate of PVL 4604 are some vestiges that can be identified which could correspond to parts of the right palatine, indicating less exposure of this bone in a ventral view. The limited exposure of the palatines is owing to their immersion in a sediment matrix that surrounds them, in addition to the obstruction of the mandible. The reconstruction of the palate in PVL 4604 confirms the preservation of both palatines, but the left retains its shape better than the right (figure 21). The right palatine is greatly reduced owing to its poor preservation. Although the left palatine also lacks some parts, it can still be distinguished as a plate-shaped bone, mainly elongated anteroposteriorly. On the left palatine of PVL 4604, the bases of two anterior processes, the base of a posterolateral process and a more complete posteromedial process can be recognized (figure 21*b*,*d*,*f*,*h*,*k*,*l**,* anp?, pop). As a result, *T. romeri* [25] appears to share the tetraradiated morphology found in the palatines of most Archosauriformes, except Phytosaurs [15]. The palatine would be located between two characteristic openings of the palate that are arranged anteroposteriorly, as seen in *Ch. bonapartei* (PULR-V 07: [36]), *Ps. ischigualastensis* (PVSJ 567: [23]), *Pi. rodriguesi* (UFRGS-PV-0464-T: [16]), and in some specimens of indeterminate Rhadinosuchins (CRILAR-Pv 491: [38]). The choanae precede the palatine, with their borders formed by the anterior processes (figure 21*a*–*g,i,k**,* anp?), while posterior to this bone is the suborbital fenestra, whose borders are derived from the posterior processes (figure 21*a*–*h,j,l**,* pop). The lateral edge of the palatine contacts the medial edge of the horizontal process of the maxilla, although there is no clarity about its contact with the jugal. Additionally, it was not possible to determine if the palatine contacts medially with the pterygoid. The micro-CT images of specimen PVL 4604 do not have sufficient resolution to discern the presence of tiny teeth organized in rows and associated with crests that run along the external surface of the palatines, as seen in *Proterochampsa barrionuevoi* [33] and *Ch. bonapartei* (PULR-V 07: [36]).

### 4.18. Pterygoid

**Figure 22.**
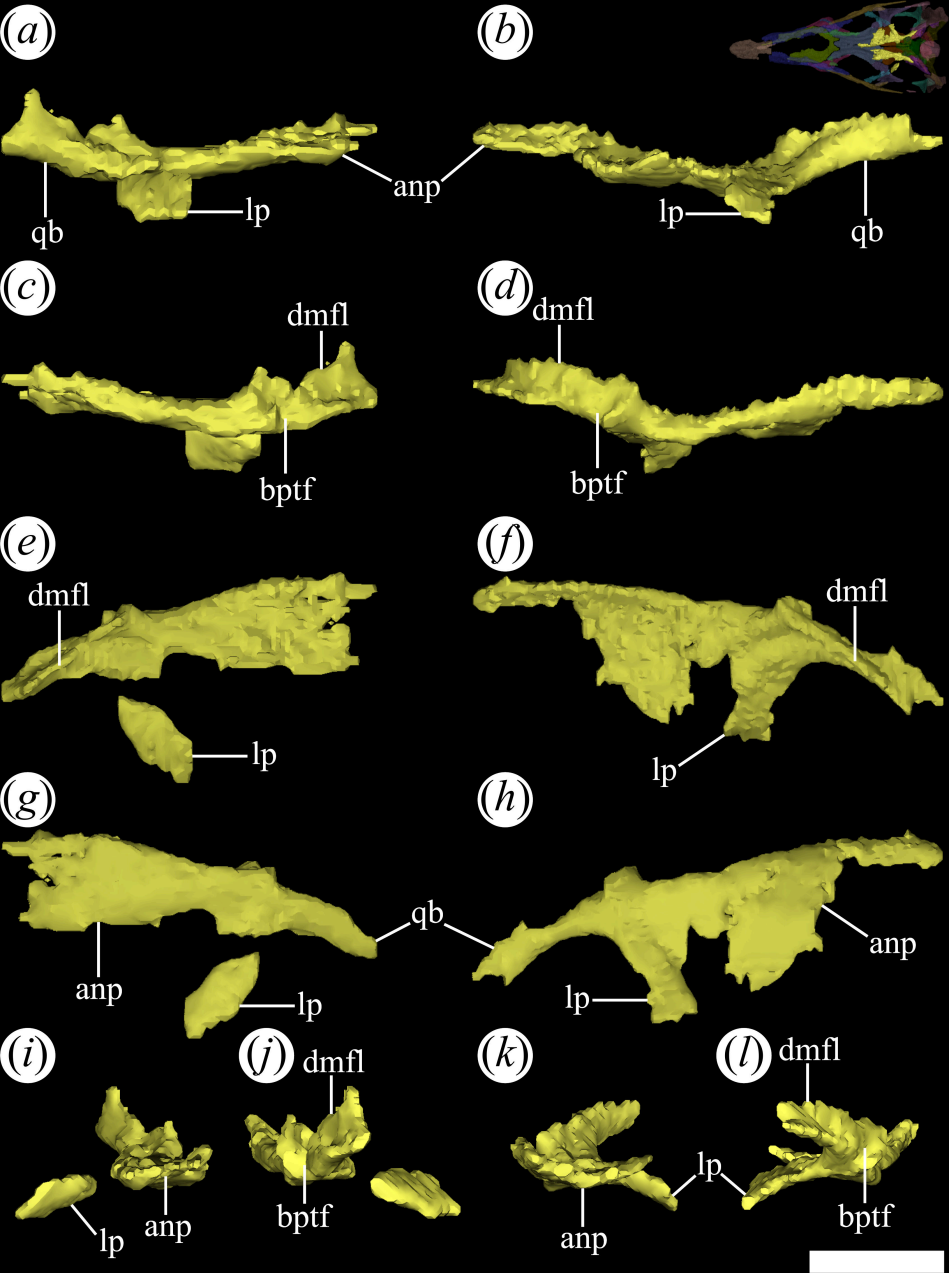
Digital reconstruction of the pterygoid of *Tropidosuchus romeri* (PVL 4604) in (*a,b*) lateral view; (*c,d*) medial view; (*e,f*) dorsal view; (*g,h*) ventral view; (*i,k*) anterior view; and (*j,l*) posterior view. (*a,c,e,g,i,j*) Right pterygoid. (*b,d,f,h,k,l*) Left pterygoid. anp, anterior process; bptf, basipterygoid facet; dmfl, dorsomedial flange; lp, lateral process; qb, quadrate branch. Scale bars equal 5 mm.

The pterygoids are paired bones visible between the hemimandibles in ventral view in PVL 4601 and 4604 [25]. In particular, the pterygoids are complete, and their palatal surface is completely exposed in PVL 4601. The pterygoid is an anteroposteriorly elongated, triradiated bone that is the most extensive component of the palate. The anterior (= ‘palatal’) process (figure 22*a*,*b*,*g*,*h*,*i*,*k**,* anp) of the pterygoid has an extensive, slightly anteromedially-to-posterolaterally oriented contact with the palatine, as occurs in other early archosauriforms [15]. By contrast, the contact between the pterygoid and the vomer is not preserved.

The lateral (= ‘transverse’) process (figure 22*a,b,e–i,k,l*, lp) is plate-like and anteroposteriorly deep. Its lateral margin is perpendicular to the midline (PVL 4601) or has a minor anterolateral slope in ventral view (PVL 4604), contrasting with the posterolateral orientation of this edge in *Ps. ischigualastensis*, *K. dornellesi* and some specimens of *Ch. bonapartei* and *Pi. rodriguesi* [16]. Micro-CT images of PVL 4604 suggest that part of the lateral process of the right pterygoid could be separated from the rest of the bone. However, this lateral process is completely preserved in the real material. The end of the lateral process of the pterygoid contacts the ectopterygoid in a suture that is difficult to identify in the available material. However, in the segmented model of PVL 4604, this same suture appears to be straight and with a minor anteromedial inclination. The suture between the pterygoids and ectopterygoids finishes anteriorly in the posterior edge of the suborbital fenestra. The posterolateral corner of the lateral process of the pterygoid is acute, as in other non-archosaurian archosauriforms, but contrasting with the rounded corner of *Doswellia kaltenbachi* [15].

From the same area where the anterior and lateral processes arise, the quadrate ramus of the pterygoid extends in a posterolateral direction (figure 22*a*,*b*,*g*,*h**,* qb). Its posterior end articulates with the pterygoid ramus of the quadrate in an anterolaterally-to-posteromedially oriented suture. The quadrate ramus of the pterygoid is longer than the lateral one and straight, in which its outer side is dorsoventrally convex and the inner one is concave. The dorsal surface of the base of the quadrate ramus possesses a broad and moderately deep groove that represents the probable osseous correlate of a cartilage attachment [66]. The dorsal edge of the quadrate ramus is very sharp (figure 22*c*–*f,j,l**,* dmfl), which matches the morphology of the dorsal flange described for some other Triassic archosauromorphs (e.g. as is the case in the tanysausrian *Tanystropheus hydroides*: [67]). There is no evidence of an arcuate flange (*sensu* [68]) in the rendering of PVL 4604. The medial surface of the base of the quadrate ramus houses a socket-like articular surface (figure 22*c*,*d*,*j*,*l**,* bptf) for the basipterygoid process of the parabasisphenoid (= basal articulation of the skull). Between the pterygoids there is a ‘V’-shaped interpterygoid vacuity that narrows anteriorly. This interpretation differs from that of Arcucci [25], in which this opening is partially obliterated halfway along the anteroposterior length of the pterygoids. However, this unusual condition could be a preservation artefact of PVL 4601 and the real shape of the interpterygoid vacuity seems better represented by PVL 4604.

Regarding the presence of pterygoid teeth, only the holotype preserves them. A row of teeth adjacent to the medial margin of the bone is oriented anteroposteriorly (= T3: [69]), as seen in *Ps. ischigualastensis* (PVSJ 567: [23]), *Ch. bonapartei* and *Gu. reigi* (PULR-V 07 and PVL 4576, respectively: [12]). A second row of teeth (= T2: [69]) is positioned on a crest with a posteromedial to anterolateral orientation and possibly extended onto the palatine (but it is not preserved), as seen in *D. kaltenbachi* (USNM 214823) and other proterochampsids (PULR-V 07: *Ch. bonapartei*, PVSJ 77: *Proterochampsa barrionuevoi*, UFRGS-PV-0065-T: *Pi. rodriguesi*). Both rows of palatal teeth converge posteriorly and finish before the level of the basal articulation. There are no palatal teeth associated with the ventral surface of the lateral process of the pterygoid, a condition shared with *Ps. ischigualastensis* (PVSJ 567: [23]), *Ch. bonapartei* (PULR-V 07: [36]), *Proterochampsa barrionuevoi* (PVSJ 77: [33]) and other eucrocopods [15].

### 4.19. Ectopterygoid

**Figure 23.**
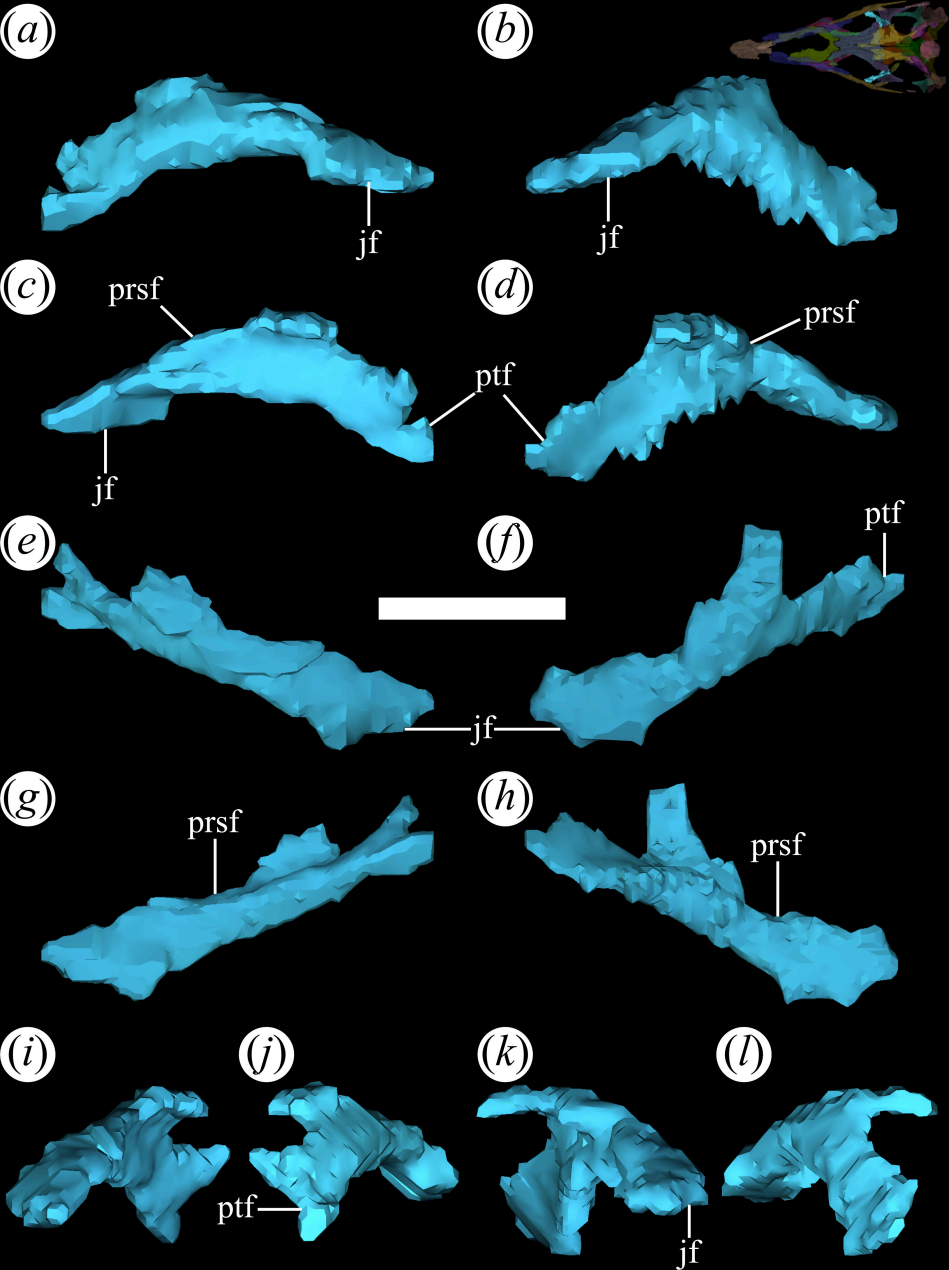
Digital reconstruction of the ectopterygoid of *Tropidosuchus romeri* (PVL 4604) in (*a,b*) lateral view; (*c,d*) medial view; (*e,f*) dorsal view; (*g,h*) ventral view; (*i,k*) anterior view; and (*j,l*) posterior view. (*a,c,e,g,i,j*) Right ectopterygoid. (*b,d,f,h,k,l*) Left ectopterygoid. jf, jugal facet; prsf, posterior rim suborbital fenestra; ptf, pterygoid facet. Scale bars equal 5 mm.

These bones form the most lateral region of the palate. The ectopterygoid is partially visible in ventral view in PVL 4601 and PVL 4604 owing to the occlusion between the lower jaw and skull. In PVL 4606, if preserved, it remains entirely obscured by the matrix. The digital model of PVL 4604 reveals that the ectopterygoid is anterolaterally-to-posteromedially oriented in ventral view. The general morphology of the bone is very similar to that of other members of Proterochampsidae [12], and its position in the palate makes it a contributor to the posterior margin of the suborbital fenestra (figure 23*c*,*d*,*g*,*h**,* prsf). The ectopterygoid contacts posteromedially the pterygoid (figure 23*c*,*d*,*f*,*j**,* ptf) and anterolaterally the internal surface of the jugal at the level of the base of the anterior process of the jugal (figure 23*a*–*c,e,f,k**,* jf). There is no evidence of contact between the anterolateral end of the ectopterygoid and the horizontal process of the maxilla, contrasting with *Proterochampsa barrionuevoi* (PVSJ 77), *Gu. reigi* (PVL 4576), *Ps. ischigualastensis* (PVSJ 567: [23]), *Ch. bonapartei* (PVL 4586) and *Pi. rodriguesi* [16]. In addition, the exposed and rendered ventral surfaces of the ecteropterygoids in PVL 4601 and 4604 lack teeth.

### 4.20. Braincase

**Figure 24.**
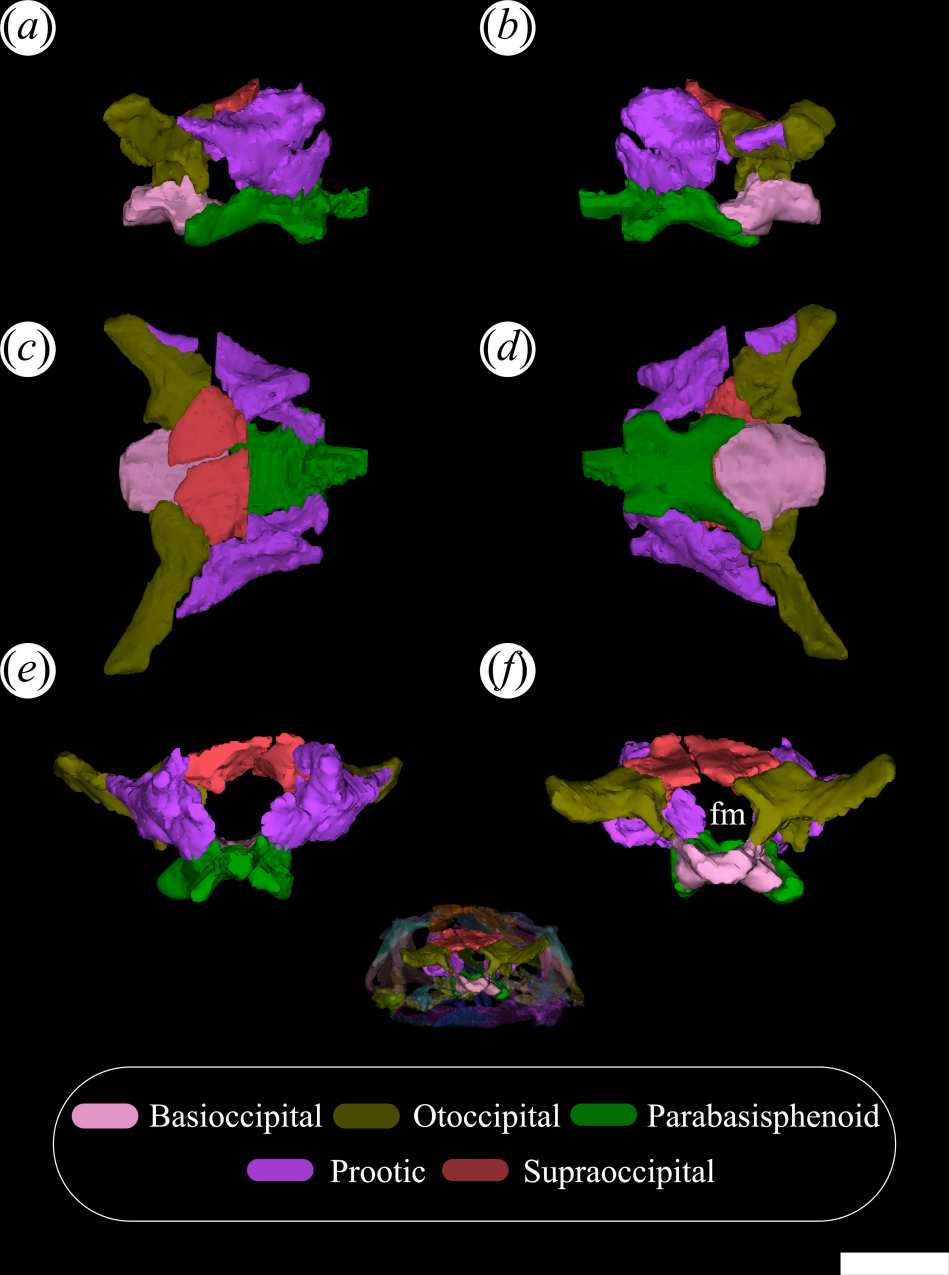
Digital reconstruction of the segmented braincase of *Tropidosuchus romeri* (PVL 4604) in (*a*) right lateral view; (*b*) left lateral view; (*c*) dorsal view; (*d*) ventral view; (*e*) anterior view; and (*f*) posterior view. fm, foramen magnum. Scale bars equal 5 mm.

This internal region of the skull has been partially examined in PVL 4601, 4604, 4606 and 4625b. Both the ventral surface and the occipital plate are the better-exposed areas, where it is possible to recognize associated structures such as the basipterygoid processes, basal tubera, occipital condyle and paraoccipital processes. Despite the existence of a previous palaeoneuroanatomical study of *T. romeri* [39], the following detailed description is primarily based on the first digital reconstruction of each of the braincase bones of PVL 4604 (figure 24), but also takes into account data obtained from direct observations of the original materials and a so far unpublished specimen (PVL 4625b; see figure 2*i*).

### 4.21. Basioccipital

**Figure 25.**
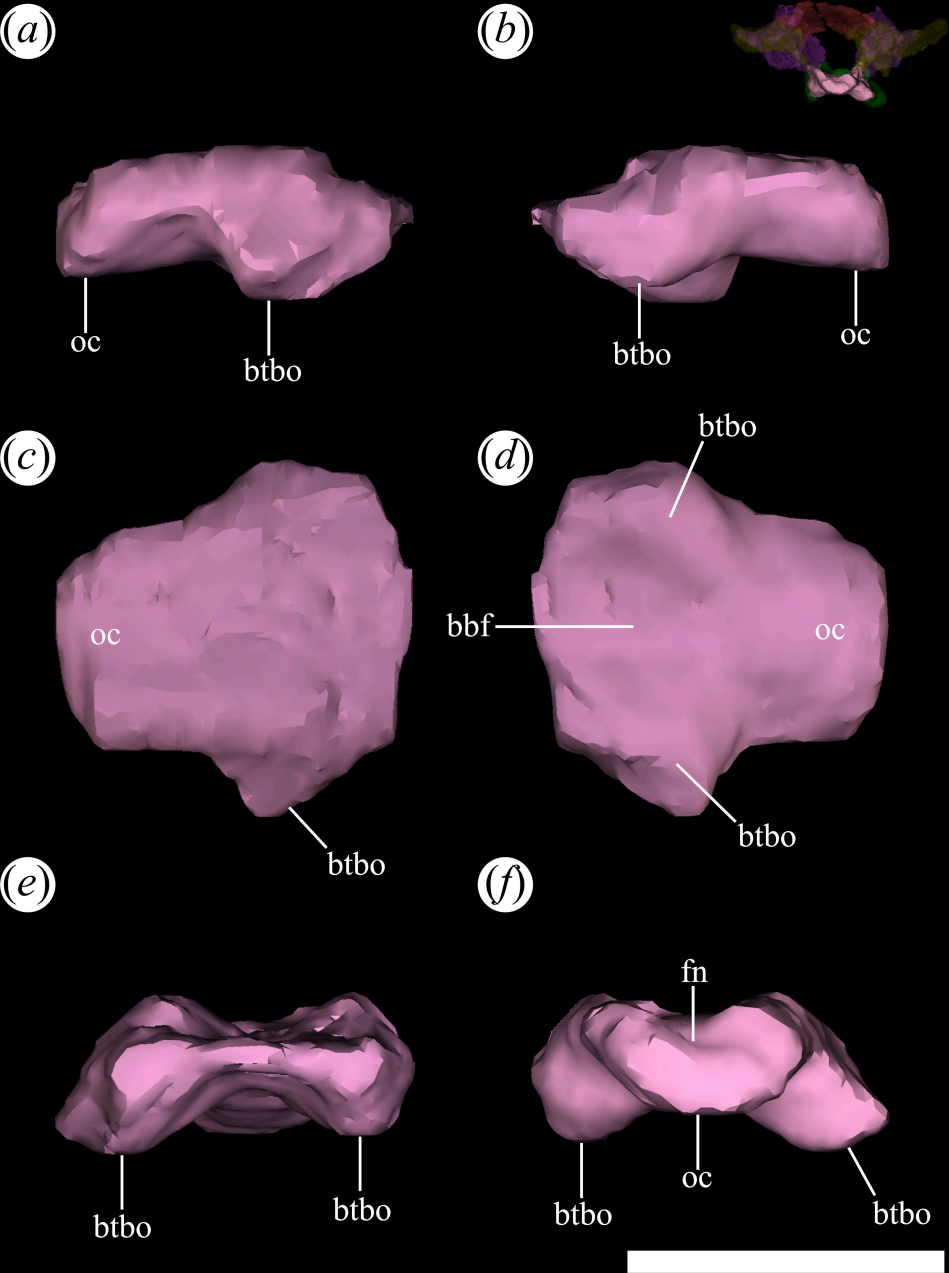
Digital reconstruction of the basioccipital of *Tropidosuchus romeri* (PVL 4604) in (*a*) right lateral view; (*b*) left lateral view; (*c*) dorsal view; (*d*) ventral view; (*e*) anterior view; and (*f*) posterior view. bbf, basioccipital-basisphenoid fossa; btbo, basioccipital part of the basal tuber; fm, foramen notochordal; oc, occipital condyle. Scale bars equal 5 mm.

The basioccipital is a single bone positioned at the posterior end of the basicranium. Its anteroposterior length, measured from the articulation with the parabasisphenoid to its posterior end, is relatively greater than its dorsoventral height, as demonstrated by the reconstructed basioccipital of PVL 4604 (figure 25). In the examined specimens of *T. romeri*, where the ventral side of the basicranium is exposed (PVL 4601, 4604 and 4606), it was not possible to accurately determine the boundary between the basioccipital and the parabasisphenoid. However, the micro-CT images of PVL 4604 suggest a linear suture with a ‘U’-shaped posteriorly oriented concavity. This suture extends through each basal tubera until reaching their distal end. The dorsal surface of the basioccipital is mostly flat and contributes to the floor of the foramen magnum and the posterior region of the endocranial cavity. Thus, the basioccipital lies ventrally between both exoccipitals and simultaneously contacts them in a suture difficult to discern owing to the low contrast of the micro-CT images. The occipital condyle (figure 25*a*–*d,f**,* oc) is formed, at least for the most part, by the basioccipital. It is a subspherical and dorsoventrally depressed condyle, similar to the condition present in the holotypes of *Ch. bonapartei* and *Ps. ischigualastensis* [70]. By contrast, in specimens PVSJ 606 and PVL 4576 of *Proterochampsa barrionuevoi* and *Gu. reigi*, respectively, the occipital condyle is slightly higher, as in *Pi. rodriguesi* [16]. A discrete and tiny pit, displaced dorsally from the centre of the condyle, is interpreted as a notochordal fossa (figure 25*f**,* fn), a trait shared with other proterochampsids [15]. The occipital condyle is considerably smaller than the foramen magnum. It is not possible to discern a neck between the occipital condyle and the basal tubera, resembling the condition in *Pi. rodriguesi*, *V. campi*, *D. kaltenbachi* and *Er. africanus* [16,63,71]. Therefore, immediately anterior to the occipital condyle, the basal tubera are developed as sub-cylindrical, ventrolaterally oriented and short processes (figure 25*a*–*f**,* btbo), as in *Ps. ischigualastensis* and *Ch. bonapartei* [70]. The basal tuber of *T. romeri* is elongated anteroposteriorly and narrow transversely. In occipital view, the ventral edge of the basal tubera is rounded. The basal tubera do not contact each other at their base, but they are completely separated, similar to *Ch. bonapartei*, *Gu. reigi*, *Ps. ischigualastensis* and *Pi. rodriguesi* [16]. The space separating both tubera opens anteriorly into a shallow, anteroposteriorly elongated fossa, which is interpreted as the basioccipital-basisphenoid fossa (figure 25*d**,* bbf) and extends to the posterior edge of the parabasisphenoid. This same fossa has also been reported in other early archosauriforms [32,70–72]. Part of the medial wall of the metotic foramen is formed by the lateral side of the basioccipital component of the basal tubera and it is particularly evident on the right side of the braincase of PVL 4604 because it is better preserved than its counterpart.

### 4.22. Otoccipital (= exoccipital–opisthotic)

**Figure 26.**
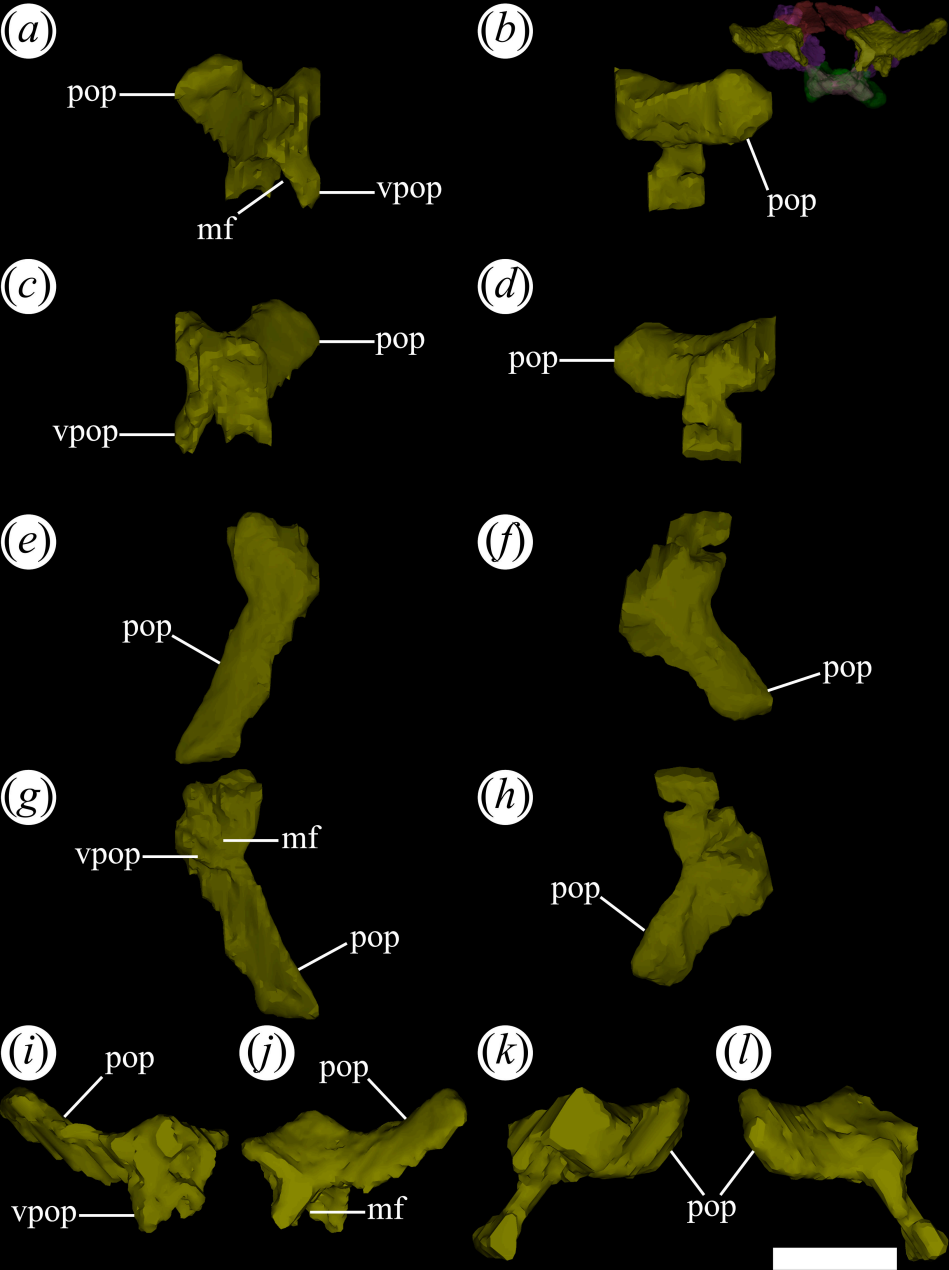
Digital reconstruction of the otoccipital of *Tropidosuchus romeri* (PVL 4604) in (*a,b*) lateral view; (*c,d*) medial view; (*e,f*) dorsal view; (*g,h*) ventral view; (*i,k*) anterior view; and (*j,l*) posterior view. (*a,c,e,g,i,j*) Right otoccipital. (*b,d,f,h,k,l*) Left otoccipital. mf, metotic foramen; pop, paroccipital process; vpop, ventral process of the opisthotic. Scale bars equal 5 mm.

The opisthotic and exoccipital bones are completely fused to each other, forming an otoccipital, in all available skulls (PVL 4601, 4604, 4606), as also occurs in many other early archosauriforms [15,71,73,74]. An anterolaterally-to-posteromedially oriented suture represents the contact between the otoccipital and the supraoccipital. The otoccipital is excluded from the dorsal border of the foramen magnum. The suture between the exoccipital and the basioccipital cannot be discerned in the real specimen nor in the micro-CT. However, when compared with other early archosauriforms (e.g. [72,74]), it is likely that the exoccipital formed the lateral wall of the foramen magnum. The inner wall of the foramen magnum is dorsoventrally concave. The exoccipital separates the foramen magnum from the metotic foramen, the latter being the opening through which cranial nerves IX–XI leave the endocranial cavity. The metotic foramen opens posterolaterally (figure 26*a*,*g*,*j**,* mf). The medial side of the otoccipital forms the most posterior inner lateral surface of the endocranial cavity, but in this region, it is not possible to identify the passage of cranial nerve XII in any of the specimens of *T. romeri*, even in the micro-CT images of PVL 4604. Trotteyn & Paulina-Carabajal [39] could not also find the passage of the cranial nerve XII in *T. romeri*. In that contribution, Trotteyn & Paulina-Carabajal [39] proposed two possible alternatives to explain its supposed absence. One of them was that cranial nerve XII has an individual exit but has not been preserved during fossilization. The other explanation claimed that this nerve also leaves the endocranial cavity through the metotic foramen. The reconstructed model of PVL 4604 would suggest that cranial nerves IX–XII share the same foramen. In addition to contacting the supraoccipital, the otoccipital also articulates with the prootic anteriorly. This suture is observable in dorsal view on both sides of the braincase, but it is better preserved on the right side. It is composed of an extensive overlapping between both bones in an anteromedially-to-posterolaterally oriented direction. The exoccipital is the main contributor to the medial surface of the metotic foramen. Since it is not possible to determine the suture between the exoccipital and the opisthotic, it is unknown but very likely that the interfenestral crest (= ‘ventral process’, = ‘ventral ramus of the opisthotic’) is formed by the opisthotic, as in other early archosauriforms (e.g. *Polymorphodon adorfi*: [75]; *Eu. capensis*: [72]; *Proterochampsa barrionuevoi*: PVSJ 606, [32]). The digital reconstruction of the braincase of PVL 4604 of *T. romeri* reveals that only the right otoccipital has partially preserved the interfenestral crest. In both posterior and lateral views, it is possible to observe how the interfenestral crest (figure 26*a*,*c*,*g*,*i**,* vpop) descends from the base of the paraoccipital process, forming the lateral edge of the metotic foramen. The poor preservation of this ventral process prevents verifying if it contacted the basal tubera and if it does with the parabasisphenoid, basioccipital or both. Anteriorly to the interfenestral crest occurs the oval fenestra, an opening through which the inner ear is reached but which, like the metotic foramen, lacks its ventral edge owing to poor preservation.

The paraoccipital processes of PVL 4604 are very well preserved and exposed, in posterior view, on both sides of the skull (figure 26*a*–*l**,* pop). The paraoccipital process is posterolaterally oriented and blade-shaped, being dorsoventrally taller than anteroposteriorly deep. Its dorsal and ventral edges are parallel to each other, in which the dorsal margin is concave dorsally and the ventral one is convex ventrally, resulting in a that possesses a slight dorsal curvature, a condition similar to that of *Eu. capensis* [72], but contrasting with the straight processes of *Ch. bonapartei* and *Proterochampsa barrionuevoi* [32]. Additionally, the posterior surface of the paraoccipital process lacks the groove present in *Proterochampsa barrionuevoi* and *Ch. bonapartei* [32]. The distal end has a slightly rounded contour and does not expand dorsoventrally, contrasting with *Er. africanus* and *D. kaltenbachi* [32]. A much more abrupt expansion of the distal end of this process is observed in the paraoccipital process of some Archosauria, such as *Arizonasaurus babbitti* [76] and *Batrachotomus kupferzellensis* [77]. Only in specimens PVL 4601 and 4606 of *T. romeri* can it be observed how the posterolateral process of the parietal rests on the paraoccipital process and adopts the same orientation. The distal end of the paroccipital process contacts the posterior surface of the squamosal.

### 4.23. Parabasisphenoid

**Figure 27.**
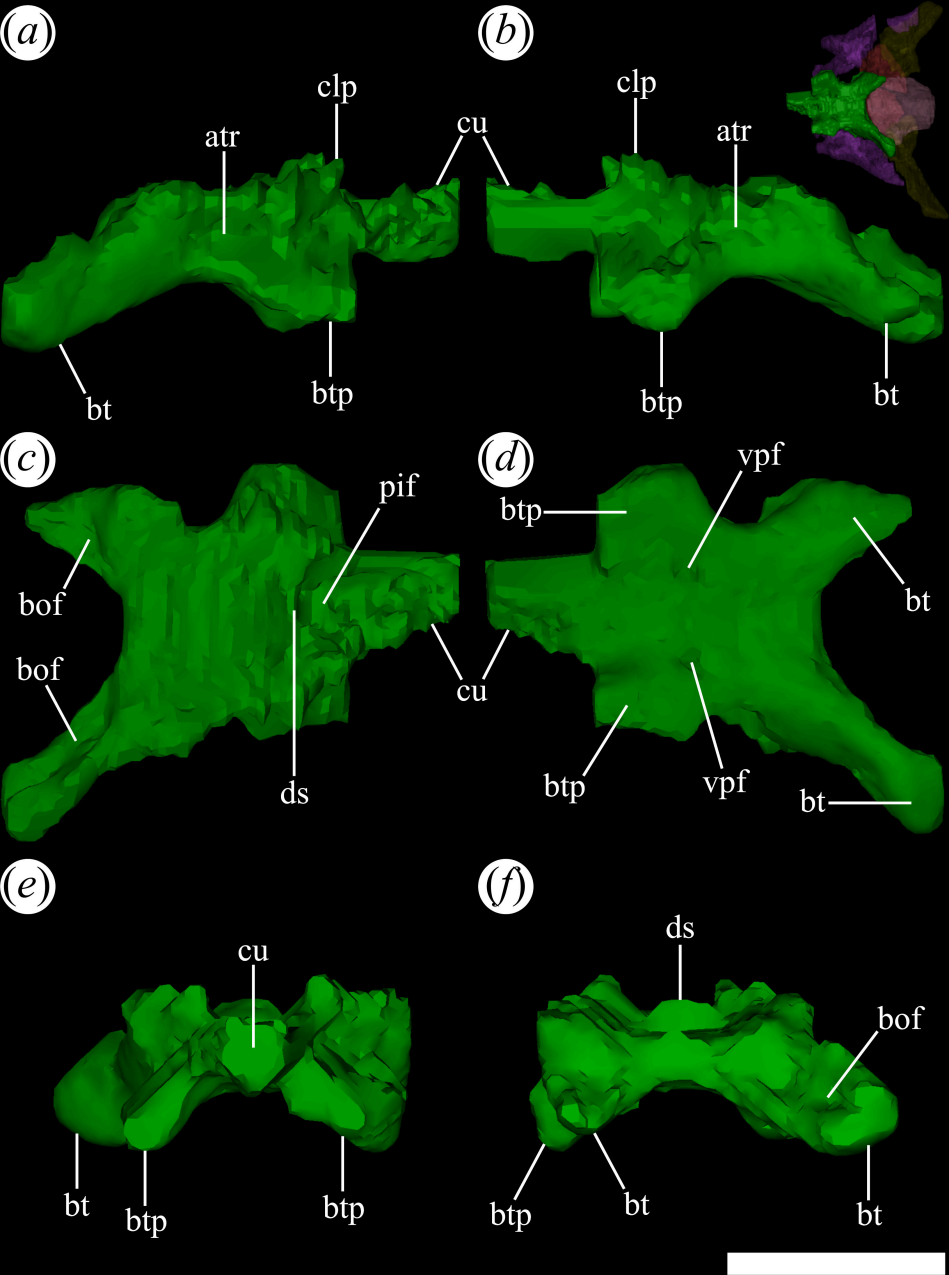
Digital reconstruction of the parabasisphenoid of *Tropidosuchus romeri* (PVL 4604) in (*a*) right lateral view; (*b*) left lateral view; (*c*) dorsal view; (*d*) ventral view; (*e*) anterior view; and (*f*) posterior view. atr, anterior tympanic recess; bof, basioccipital fossa; bt, basal tuber; btp, basipterygoid process; clp, clinoid process; cu, cultriform process; ds, dorsum sellae; pif, pituitary fossa; vpf, ventral paired foramina on parabasisphenoid, probably for the cerebral internal carotid artery. Scale bars equal 5 mm.

The parabasisphenoid represents the anterior continuation of the floor of the endocranial cavity, becoming its main component. Together with the basioccipital, it forms the basicranial region [78]. The length of the parabasisphenoid, measured from the most anterior point of the basipterygoid processes to the most posterior end of the basal tubera, is slightly lower than the maximum width between the lateral edges of the distal end of the basal tubera. A similar pattern is observed in the parabasisphenoid of *Ch. bonapartei* (PULR-V 07: [24]) and *Ps. ischigualastensis* (PVSJ 567; [70]), although in the latter species, the parabasisphenoid may appear shorter because of damage on its posterior edge [70]. The micro-CT images of PVL 4604 show that the dorsoventral thickness of the parabasisphenoid thins out at its central region, between the basipterygoid processes and the basal tubera, and then gradually increases towards the lateral edges. The bases of the basal tubera and basipterygoid processes are aligned in the same plane in all the specimens of *T. romeri*, resulting in a completely horizontal orientation of the parabasisphenoid in lateral view. This condition has also been reported in the holotype of *T. romeri* (PVL 4601; [39]) and *Ch. bonapartei* [15,36], contrasting with the condition found in *Er. africanus* and *Eu. capensis* [15,31,72]. The digitally reconstructed parabasisphenoid of PVL 4604 reveals the presence of two depressed regions. The first is positioned on the lateral surface and it is anteroposteriorly elongated and dorsoventrally narrow. Its location suggests a homology with the anterior tympanic recess (figure 27*a*,*b**,* atr), a feature shared with several archosaurs (e.g. *Silesaurus opolensis*), *Eu. capensis* [72] and *Ch.s bonapartei* [36], but absent in other non-archosaurian archosauriforms [15,31]. The second depression is smaller and occurs dorsal to the base of the basipterygoid processes, resembling the condition in *Proterochampsa barrionuevoi* [32]. Above this second depression are the anterodorsally projecting clinoid processes (figure 27*a*,*b**,* clp), with acute apices and broad bases connected by the dorsum sella. The articulation between the parabasisphenoid and the prootic can be identified in the digital rendering of PVL 4604 and its irregular shape is a result of low contrast in the micro-CT images. In lateral view, this suture is mainly anteroposteriorly oriented and its anterior and posterior halves are anteromedially and posteromedially curved, respectively.

The cultriform process (= ‘parasphenoid rostrum’) is positioned anteriorly to the basipterygoid processes and invades the interpterygoid vacuity (figure 27*a*–*d**,* cu). It is a horizontal and anteriorly tapering process, being anteroposteriorly elongated, dorsoventrally low and narrow transversely. The ventral exposure of the cultriform process in PVL 4606 allows determination of its complete length, being at least equivalent to that of the main body of the parabasisphenoid. In the three-dimensional model of PVL 4604, the partially preserved cultriform process has a V-shaped cross-section, with a deeply excavated dorsal surface, as occurs in *Eu. capensis* [72], but contrasting with the oval cross-section and the absence of a dorsal groove in the cultriform process of *Proterochampsa barrionuevoi* (PVSJ 606; [32]).

The basipterygoid processes (figure 27*a,b,d–f*, btp) are developed as lateromedially compressed, finger-like structures, with a broad base in the anteroposterior direction and a blunt distal end. These processes protrude from the anterolateral corner of the parabasisphenoid body in a ventrolateral direction, as seen in *Ch. bonapartei*, *Ps. ischigualastensis* and *Eu. capensis*, although in the latter species, they also possess a slight posterior orientation [70,72]. These processes are less ventrally and more laterally oriented in *Proterochampsa barrionuevoi* [70], thus their distal ends diverge more conspicuously from each other than in the other species. The basipterygoid processes are smaller than the basal tubera. A rounded lateral ridge connects the posterior region of the base of the basipterygoid process with the basal tubera of the parabasisphenoid. As a consequence, these ridges border laterally the ventral surface of the parabasisphenoid, which possesses a discrete concavity, but without a well-defined recess, resembling the condition in *Pi. rodriguesi* [16]. The lateral surface of the basipterygoid processes articulates with the medial side of the quadrate ramus of the pterygoid. By contrast, the articulation with the pterygoid covers the lateral and ventral surface of the basipterygoid processes in *Proterochampsa barrionuevoi* [70].

The foramina associated with the entry of the internal carotid arteries were not identified by Trotteyn & Paulina-Carabajal [39] in PVL 4601. Here, after examining the exposed ventral surface of the basicranium of *T. romeri*, we identified these foramina immediately posteromedial to the base of the basipterygoid processes, which was subsequently confirmed by the rendered parabasisphenoid of PVL 4604 (figure 27*d**,* vpf). This is the same position that these foramina occupy in several Archosauriformes, including *Ps. ischigualastensis* (PVSJ 567), *Ch. bonapartei* (PULR-V 07) and *Pi. rodriguesi* [16], but it contrasts with their more posterior location, near the basal tubera, in *D. kaltenbachi* [71], *Proterochampsa barrionuevoi* [33] and *Archaeopelta arborensis* [79]. The internal carotid arteries enter through these small openings and continue through bony channels that pierce the parabasisphenoid as they converge towards the midline and individually open into a deep pituitary fossa (figure 27*c**,* pif). This fossa is positioned dorsal to the base of the cultriform process and, during life, hosted the pituitary gland. The posterior edge of the pituitary fossa is separated from the rest of the floor of the endocranial cavity by a dorsally raised dorsum sella (figure 27*c*,*f**,* ds). The pituitary fossa is anteroposteriorly shorter than the rest of the floor of the endocranial cavity, resembling the condition in some archosaurs (e.g. *Ari. babbitti*: [76]; *Massospondylus carinatus*: [80]), but contrasting with *Proterochampsa barrionuevoi* [32].

Although the basal tubera are well exposed ventrally, it is difficult to determine the degree of contribution of the basioccipital and the parabasisphenoid to these structures. The parabasisphenoid contribution to the basal tubera is longer than transversely wide (figure 27*a,b,d–f*, bt), which differs from the condition in *Proterochampsa barrionuevoi* [32]. Good preservation in PVL 4604 exposes a smooth concavity on the inner side (figure 27*c*,*f*, bof), near its distal end, that articulates and envelops the anteroventral side of the basioccipital contribution to the basal tubera, as in *Proterochampsa barrionuevoi* [32]. The presence of a semilunar depression on the lateral surface of the posterior region of the parabasisphenoid cannot be completely ruled out because the region is only accessible through the three-dimensional renderings, and lack of definition could be hampering its recognition.

### 4.24. Prootic

**Figure 28.**
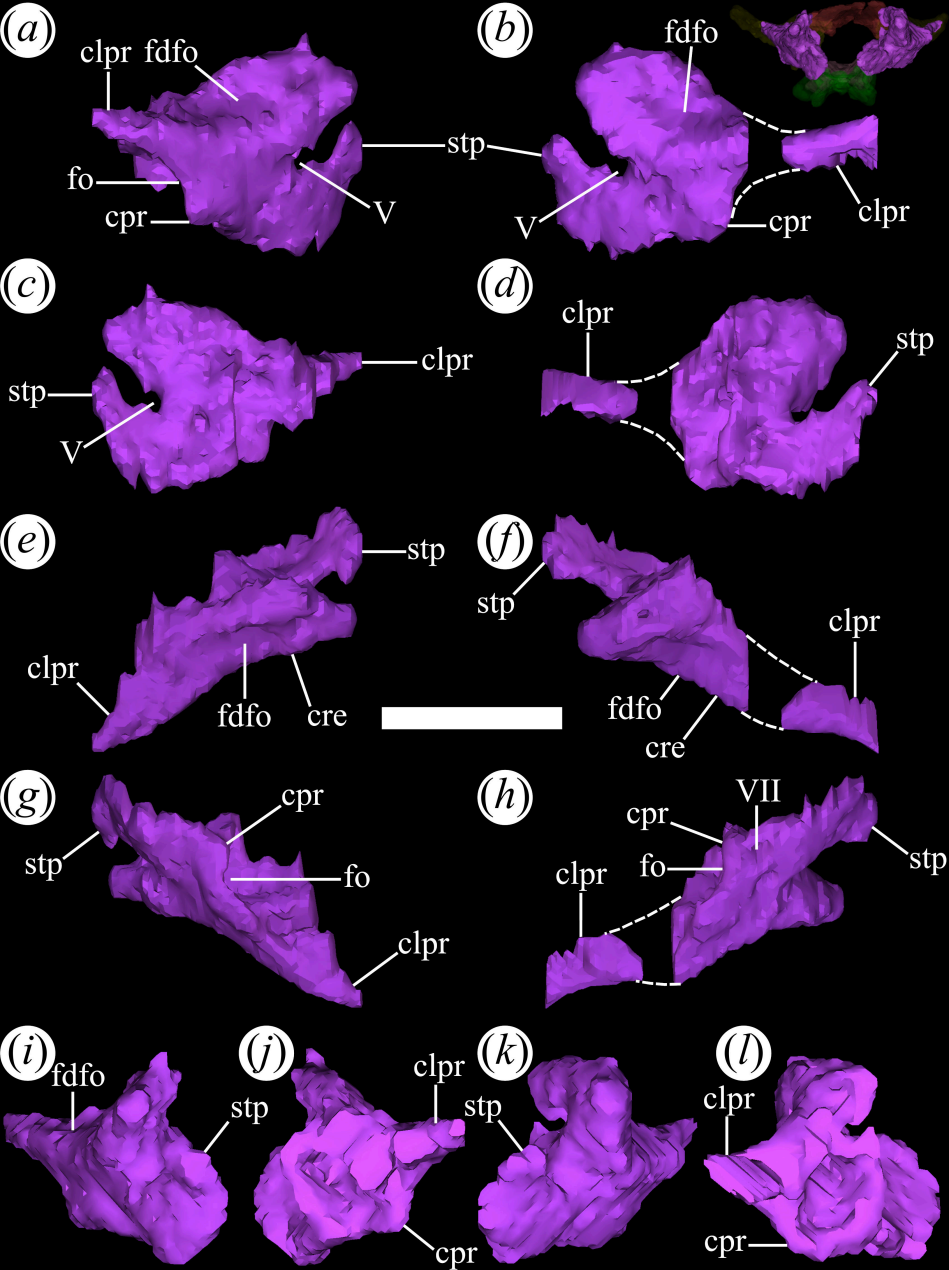
Digital reconstruction of the prootic of *Tropidosuchus romeri* (PVL 4604) in (*a,b*) lateral view; (*c,d*) medial view; (*e,f*) dorsal view; (*g,h*) ventral view; (*i,k*) anterior view; and (*j,l*) posterior view. (*a,c,e,g,i,j*) Right prootic. (*b,d,f,h,k,l*) Left prootic. clpr, caudolateral process of the prootic; cpr, crista proótica; cre, crest; fdfo, fossa dorsal to fenestra ovalis; fo, fenestra ovalis; stp, subtrigeminal process; V, trigeminal nerve notch; VII, foramen of nerve seven. Scale bars equal 5 mm.

The prootic is only exposed in the referred specimen of *T. romeri* PVL 4625, and it has been virtually isolated in PVL 4604 based on the micro-CT data. The good preservation of the prootic in PVL 4625 facilitates comparison with its digital rendering in PVL 4604 and, eventually, evaluation of the fidelity of its reconstruction. The prootic is a paired bone that, in the digital braincase of PVL 4604, has better preservation on the right side than on the left. In addition to its important contribution to the lateral wall of the braincase, the prootic is also partially involved in the formation of the bony labyrinth occupied by the semicircular canals of the inner ear, as has been documented in several archosaurs [81–83]. However, it was impossible to reconstruct the inner ear based on the micro-CT images of PVL 4604 because they reveal an apparent collapse of the internal ear cavities on both the right and left sides of the braincase. An incipient crest (figure 28*e*, cre) separates the lateral surface of the prootic into dorsal and ventral halves. The dorsal side of the prootic, with a dorsomedial to ventrolateral slope, possesses a shallowly depressed surface (figure 28*a*,*b*,*e*,*f*,*i**,* fdfo) that could be homologous to the fossa found in *Ps. ischigualastensis* [70] and *Arc. arborensis* [79], although in the latter species, it is proportionally smaller. A crest like that reported here has also been reported in a specimen of *Eu. capensis* [72], and in the dinosaur *Megapnosaurus rhodesiensis* [84,85]. In the virtually exposed occipital region of the skull of PVL 4604, a non-ossified region is observed dorsal to the foramen magnum and is absent in PVL 4601, 4606. This opening is interpreted as a preservation artefact related to the partial detachment of the braincase from the rest of the cranium, followed by a subsequent ventral displacement. This could explain the atypical dorsal contact of the prootic with the posterolateral process of the parietal, instead of its usual articulation with the concave ventral side of the parietal. It is important to note the posterior and partial contact that the prootic retains with the anterodorsal edge of the supraoccipital in the three-dimensional braincase of PVL 4604, also occurs in *Ps. ischigualastensis* and several other early archosauriforms [70]. As previously mentioned, the prootics contact ventrally on both sides of the parabasisphenoid, unlike the ventral articulation between prootics along the midline in *Er. africanus* [73], *Eu. capensis* [86] and in some archosaurs [76,77].

The absence of the laterosphenoid results in an incomplete trigeminal foramen (passage of cranial nerve V) because it lacks its ossified anterior edge, while the dorsal, posterior and ventral edges are exclusively formed by the prootic, as in *Eu. capensis* [72] and the vast majority of other archosauriforms [15]. The cranial nerve V foramen is large and with an anterolateroventral orientation (figure 28*a*–*c,v*). Additionally, it is oval, with an anterodorsally-to-posteroventraly oriented main axis, resembling the condition in *V. campi* [63] and *Eu. capensis* [86].

The subtrigeminal process is strongly developed in an anterodorsal direction (figure 28*a*–*i,k**,* stp). It is a transversely thin, laminar process developed in the same plane of inclination as the alar process of the prootic in dorsal view. The lateral surface of the subtrigeminal process lacks a ridge for the insertion of the *protractor pterygoideus muscle*, as in many archosaurs (e.g. *Silesaurus* and *Arizonasaurus*: [74,76,87]), but contrasting with its presence in some basal early archosauriforms (e.g. *Garjainia* [88], and *Fugusuchus*; [74]). Dorsal to the trigeminal foramen there is a process that is shorter than the subtrigeminal process, with a greater transverse thickness, which hollows towards the inner ear and has a blunt anterior end.

The foramen for the passage of the cranial nerve VII (figure 28*h*,vii) is much smaller in diameter than the cranial nerve V, and its margins are completely formed by the prootic. Like in *Er. africanus*, its location on the posteroventral edge of the prootic is also consistent with its posteroventral orientation. It was not possible to identify the foramen corresponding to the abducens nerve (VI), either the insertion fossa of the *bulbi retractor musculature* in the prootic.

A weakly developed semicircular prootic crest (figure 28*a*,*b*,*g*,*h*,*j*,*l**,* cpr) extends posterodorsally-to-anteroventrally along the ventrolateral edge of the prootic. The prootic crest forms part of the anterolateral edge of the oval fenestra (figure 28*a*,*g*,*h**,* fo), as in *Eu. capensis* [86]. A wing-like process protrudes posterolaterally from the lateral surface of the prootic, with a broad base that gradually tapers towards its distal end (figure 28*a*–*h,j,l**,* clpr). This process articulates posteriorly with the opisthotic when it overlaps the proximal half of the anterior surface of the paraoccipital process, resembling the condition in *Proterochampsa barrionuevoi* [32] and *Rh. gracilis* [37].

### 4.25. Supraoccipital

**Figure 29.**
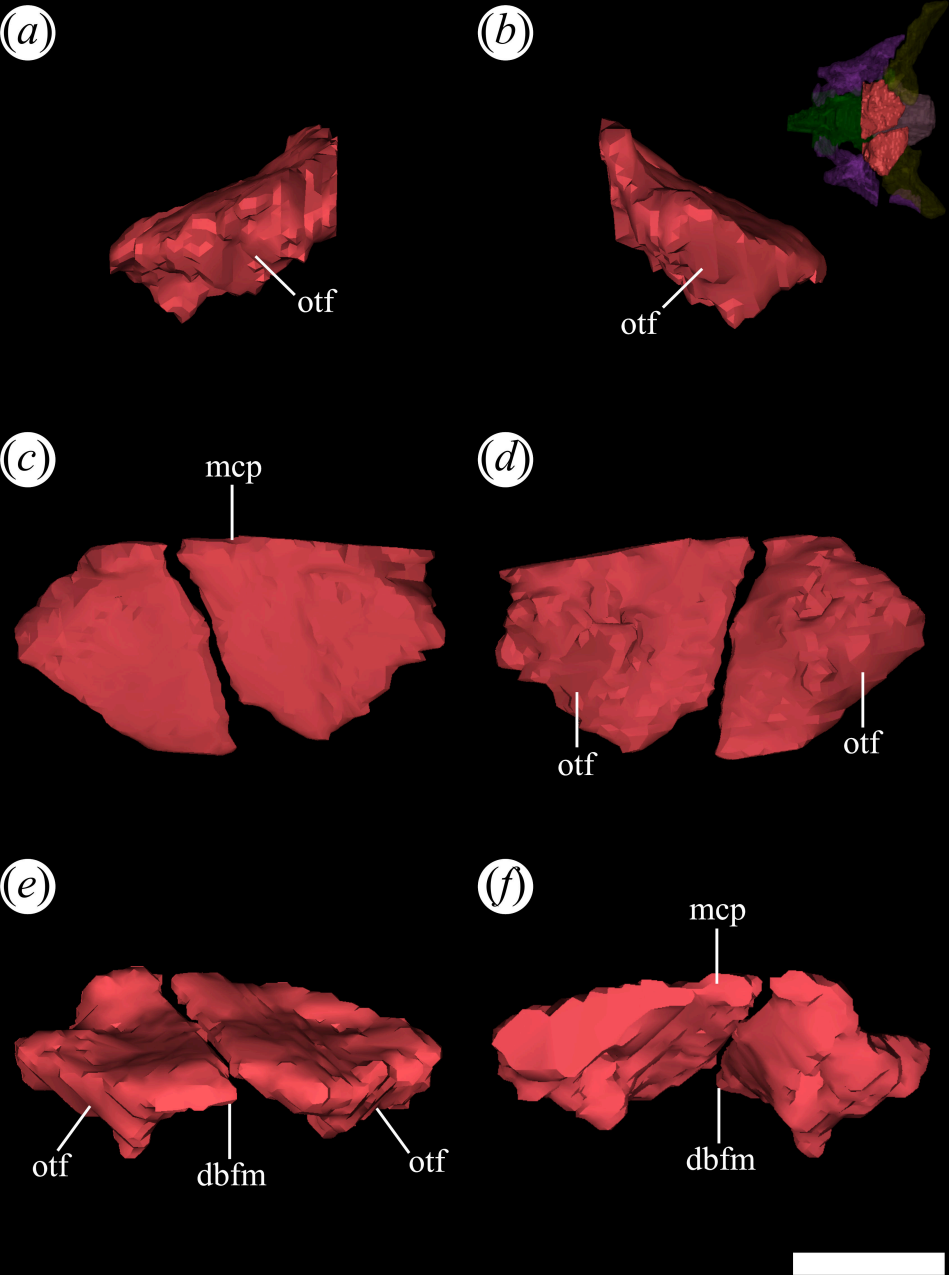
Digital reconstruction of the supraoccipital of *Tropidosuchus romeri* (PVL 4604) in (*a*) right lateral view; (*b*) left lateral view; (*c*) dorsal view; (*d*) ventral view; (*e*) anterior view; and (*f*) posterior view. dbfm, dorsal border of the foramen magnum; mcp, margin of contact with the parietal; otf, otoccipital facet. Scale bars equal 5 mm.

This bone is well preserved in PVL 4604, but with a slightly inclined fracture that crosses it from its dorsal to ventral edge. This bone is also exposed in PVL 4601, but it is not preserved/exposed in PVL 4606. In posterior view, the sutural contact of the supraoccipital with the right otoccipital can be clearly identified in PVL 4604, but it is not the case with the left otoccipital. Thus, the left articulation suture is inferred (figure 29*a*,*b*,*d*,*e**,* otf). In PVL 4601, although no signs of deterioration are evident on the external surface of the supraoccipital, it was not possible to discern its lateral limits. The supraoccipital is located dorsally and forms the dorsal edge of the foramen magnum (figure 29*e*,*f**,* dbfm), as in *Eu. capensis* [72] and *D. kaltenbachi* [71]. However, the supraoccipital remains excluded from the dorsal edge of the foramen magnum in *Er. africanus* by the contact of the fused exoccipital/opisthotic [73]. The bone possesses a strong anterodorsal to posteroventral orientation and forms part of the most posterior region of the endocranial roof. The digitally reconstructed braincase of PVL 4604 reveals a pentagon-shaped, transversely elongated supraoccipital. The digital supraoccipital of PVL 4604 shows that its thickness is somewhat variable, being thinner at its centre and gradually increasing towards the lateral edges. The external surface does not show ornamentation, being smooth throughout its extension, contrasting with the presence of a median keel in the supraoccipitals of *V. campi* [63], *D. kaltenbachi* [71], *Arc. arborensis* [79] and several archosaurs (e.g. *Batrachotomus kupferzellensis*; [77]). The partial articulation of the supraoccipital with the parietal (figure 29*c**,* mcp), as seen in PVL 4601, occurs through its convex and rounded dorsal edge, which also represents the longest side, so it also contacts part of the prootic. On the other hand, the ventral edge, besides being the narrowest in length, has a rounded notch that delimits the foramen magnum. The suture between the supraoccipital and the otoccipital could be identified.

### 4.26. Lower jaw

Four specimens of *T. romeri* (PVL 4601, 4604, 4606, 4625) preserve both hemimandibles in articulation with their skulls. As a consequence, several anatomical features remain hidden. Despite these limitations, the lower jaw of PVL 4604 was successfully digitally isolated, providing a more complete view of each hemimandible. Additionally, some of the bones of the left hemimandible were individualized. Unfortunately, the same could not be achieved with the right hemimandible, as it appears to be more fragmented, particularly in the posterior half and at the level of the external mandibular fenestra; the right splenial was the only bone that could be segmented individually. Smaller features, such as neurovascular foramina, could not be recognized owing to the low resolution/lack of contrast of the available micro-CT data.

### 4.27. Dentary

**Figure 30.**
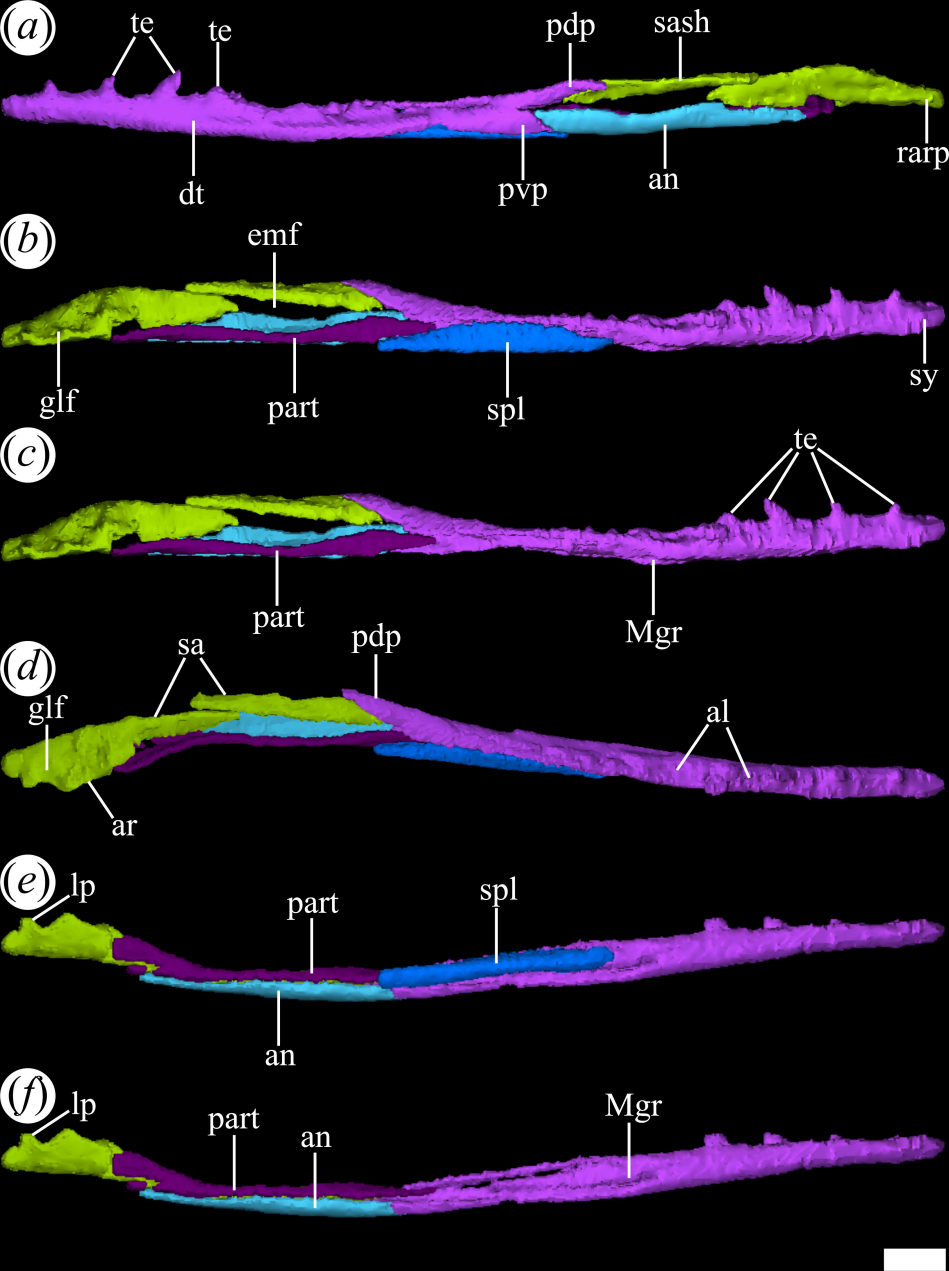
Digital reconstruction of the left hemimandible of *Tropidosuchus romeri* (PVL 4604) in (*a*) lateral view; (*b*) medial view; (*c*) medial view without splenial; (*d*) dorsal view; (*e*) ventral view; and (*f*) ventral view without splenial. al, alveolus; an, angular; ar, articular; dt, dentary; emf, external mandibular fenestra; glf, glenoid fossa; lp, lobe-shaped process; Mgr, Meckelian groove; part, prearticular; pdp, posterodorsal process; pvp, posteroventral process; rarp, retroarticular process; sa, surangular; sash, surangular shelf; spl, splenial; sy, symphysis; te, teeth. Scale bars equal 5 mm.

It is the major component of the mandible, occupying more than half of each mandibular ramus. The dentary is a strongly anteroposteriorly elongated and low bone throughout its extension (figure 30*a**,* dt), as in *Rh. gracilis* (SNSB-BSPG AS XXV 50, 51: [37]), *Gu. reigi* (PULR-V 05 and PVL 4576) and *Ch. bonapartei* (PULR-V 07: [36]). At the anterior end of the mandible, the medial sides of both dentaries contact each other in a relatively anteroposterior short mandibular symphysis (figure 30*b*, sy) that aligns with the sagittal plane of the skull. The contact surface between dentaries is flat. In dorsal and ventral view, the anterior end of the dentary of *T. romeri* (PVL 4601, 4604) tapers to a point (figure 30*d*–*f*) and contrasts with the transverse expansion of the dentary of *Pi. rodriguesi* and *Rh. gracilis* [16]. Posterior to the mandibular symphysis, the medial surface of the dentary is partially exposed and the rest is covered by the articulation with the splenial. The posterior end of the dentary bifurcates into a posterodorsal (figure 30*a*,*d**,* pdp) and a posteroventral process (figure 30*a**,* pvp) that articulates with the surangular and angular, respectively. Among the Proterochampsia, *L. somnii* [61] possesses a different condition, as a third process is identified at the posterior end of the dentary. A similar situation has also been indicated in *Er. africanus* and *Ga. prima* [15,64]. Like other Proterochampsidae, the posterior bifurcation of the dentary contributes to the anterior edge and part of the dorsal and ventral edge of the external mandibular fenestra [12,15]. The posterodorsal process of the dentary is more developed than the posteroventral one, and its contact with the surangular defines an anteroventrally-to-posterodorsally oriented suture. The posteroventral process articulates with the angular in an anterodorsally-to-posteroventrally oriented suture. This process contributes to the anteroventral corner of the external mandibular fenestra. The ratio between the length of the dentary, measured from its anterior end to the anterior end of the external mandibular fenestra, and its minimum height is 16.96 in PVL 4604. A similar proportion is also present in PVL 4601 and denotes a very gracile element. This condition is also shared by *Ch. bonapartei* and *Gu. reigi* [16]. By contrast, the ratio between length and minimum height is much lower in the dentary of *Pi. rodriguesi* and *Ce. binsfeldi* [16].

Digital removal of the splenial reveals that the Meckelian groove (figure 30*c*,*f**,* Mgr) is positioned on the ventral half of the medial surface of the dentary. This groove is dorsoventrally low and elongates anteroposteriorly. Its anterior end extends up to the level of the fifth tooth, without reaching the level of the mandibular symphysis, as in *Ch. bonapartei* (PULR-V 07: [36]). The Meckelian groove extends posteriorly, but does not reach the anterior edge of the external mandibular fenestra. The lateral and ventral surfaces of the dentary are connected by a sharp ventrolateral edge, similar to *Ch. bonapartei* (PULR-V 07), *Gu. reigi* (PVL 4576), *Ps. ischigualastensis* (PVSJ 567: [23]) and *Rh. gracilis* (SNSB-BSPG AS XXV 50: [37]).

The digital isolation of the mandible of PVL 4604 exposes the alveolar margin of the dentary. In both dentaries, the posterior region of the alveolar margin is damaged, while the anterior one shows some teeth *in situ*. The left dentary preserves four teeth of different sizes, with the third being the largest and best preserved (figure 30*a*,*c**,* te). At least three teeth are recognized in the right dentary, with apparent signs of postmortem damage. The teeth of the dentary are considerably smaller than those of the maxilla, but they are similar in shape as they are labiolingually compressed and recurved backwards, ending in a pointed apex. The low resolution of the micro-CT and doubtful preservation of small features make it impossible to determine the presence of serrated edges on these teeth. Owing to the deterioration of some alveoli, the exact number of teeth in the dentary of *T. romeri* (PVL 4604) cannot be precisely determined. However, in the second micro-CT of PVL 4604, the resolution was sufficient to discriminate a minimum of 15 tooth positions (figure 9*b*). This is congruent with dentary tooth counts in other proterochampsid species, such as *Ch. bonapartei* (16 teeth: [36]), although some others have a higher tooth count, such as *Rh. gracilis* (23 teeth: [37]) and *Pi. rodriguesi* (17 teeth: [16]).

### 4.28. Splenial

This bone is preserved in PVL 4601, 4604 and 4606, but only in PVL 4604 was it possible to recognize its overall shape in medial view in a real specimen. The micro-CT scan of PVL 4604 revealed that the splenial had been partially preserved in both hemimandibles. It is a transversely thin and anteroposteriorly elongated, plate-shaped bone that extensively contacts the medial surface of the dentary and covers the Meckelian groove in medial view (figure 30*b*,*e**,* spl), as in other early archosauriforms (e.g. [24]). The maximum height of the splenial is reached in the central region and decreases towards the posterior and anterior ends. At the level of its maximum dorsoventral development, the ventral edge of the splenial strongly curves laterally and contributes to the formation of both the medial and ventral surfaces of the hemimandibles. The posterior end of the splenial articulates with the prearticular, as occurs in *Ch. bonapartei* [36]. The suture between the splenial and the prearticular is straight, with a slight anterodorsal to posteroventral orientation. There is no evidence of a contract between the splenial and angular and surangular, respectively, contrasting with the condition present in other proterochampsids (e.g. *Pi. rodriguesi*: [16]), but this could be a result of poor preservation.

### 4.29. Surangular

It is one of the main components of the post-dentary region of the mandible. The occlusion between the skull and lower jaw in PVL 4604 and 4606 covers most of the surangular in lateral view because the posterior process of the jugal overlaps the surangular. However, the surangular can be observed in ventrolateral view. In PVL 4601, the surangular is more exposed but poorly preserved. Based on the rendering of the lower jaw of PVL 4604, the left surangular is preserved in two parts owing to a fracture around mid-length. Despite this, its preservation is better than in the right surangular (figure 30*d**,* sa). In none of the two hemimandibles was it possible to determine the suture between the surangular and articular; thus, it is impossible to establish the posterior extent of the surangular. The surangular almost entirely forms the dorsal edge of the external mandibular fenestra. The dorsolateral surface of the surangular has a distinct shelf-like structure (figure 30*a**,* sash) that runs anteroposteriorly, but it is more laterally developed in other proterochampsids [12,15,16,33,36,42]. This ridge ends in a sharp, anteroposteriorly convex lateral margin. The jugal and quadratojugal rest on this structure when the jaw is closed [12,15]. The anterior end of the surangular extends beneath the posterodorsal process of the dentary in lateral view, but it does not reach the anterior end of the external mandibular fenestra. Thus, the dentary also has a small contribution to the dorsal edge of the mandibular fenestra. The surangular expands dorsoventrally posteriorly to the level of maximum height of the external mandibular fenestra. The ventral edge of the surangular contacts the angular in a suture with a slight posteroventral orientation, and that extends from the posterior end of the mandibular fenestra to the ventral edge of the mandible. On the lateral surface of the surangular, no perforation is observed that could be interpreted as an anterior or posterior foramen, a feature shared with *Proterochampsa barrionuevoi* [33], *Gu. reigi* (PULR-V 05, PVL 4576), *D. kaltenbachi* [71] and *Proterochampsa nodosa* [42]. This condition is variable in *Ch. bonapartei* because MCZ 4037 has a posterior surangular foramen, but it is absent in the holotype (PULR-V 07; [15]). The posterior end of the surangular should have housed part of the glenoid fossa, as in other archosauromorphs. However, the surangular cannot be differentiated from the articular and, as a result, the glenoid region of the mandible is described below as part of the articular bone.

### 4.30. Angular

The angular is exposed in lateral view in PVL 4601, 4604 and 4606, but it is poorly preserved in the former specimen. The reconstruction of PVL 4604 shows that the angular is severely damaged in the right hemimandible, being limited to a small portion of its posterior end in dorsal connection with part of the right surangular. Unfortunately, it has not been possible to identify the suture at the contact between the right angular and surangular. By contrast, the preservation of the left angular is considerably better, allowing assess of the morphology of this element.

The angular is an anteroposteriorly elongated bone that extends beneath the external mandibular fenestra (figure 30*a*,*e*,*f**,* an). Its dorsal edge is gently concave, representing the ventral edge of the mandibular fenestra. The ventral edge of the bone is straight to slightly convex. Anteriorly, the angular contacts ventrally the posteroventral process of the dentary. A slight medial curvature is evident on its ventral edge, articulating with the prearticular in a linear suture that extends anteroposteriorly in ventromedial view (figure 30*e*,*f*). The ventral edge of the angular is smooth, lacking any ridge, similar to most rhadinosuchines (*Ch. bonapartei*: PULR-V 07; *Gu. reigi*: PULR-V 05, PVL 4576; *Ps. ischigualastensis*: PVSJ 567; *Pi. rodriguesi*: UFRGS-PV-0464-T). This contrasts with the presence of a longitudinal keel on the angular of both species of *Proterochampsa* [12,15,33,42]. The posterior end of the angular tapers along its contact with the surangular until it ends in a pointing end that does not reach the level of the glenoid fossa. The suture with the surangular is not elevated on a longitudinal crest, contrasting with the condition in *Ch. bonapartei* (PULR-V 07: [36]) and *Gu. reigi* (PVL 4576).

### 4.31. Prearticular

It was not possible to identify this bone in the holotype of *T. romeri* (PVL 4601), but in the referred specimens, the prearticular is clearly exposed in PVL 4604 and PVL 4606—but only part of its posterior end is exposed in the latter specimen. The prearticular is anteroposteriorly elongated and positioned on the medial side of the post-dentary region. It is a slender, rod-shaped element (figure 30*b*,*c*,*e*,*f**,* part). Its central region is extremely thin and increases in dorsoventral height towards both anterior and posterior ends. The anterior portion of the prearticular forms the posterior continuation of the medial wall of the Meckelian groove. The anterior end tapers dorsoventrally and establishes a double contact, dorsally with the posterodorsal process of the dentary and ventrally with the splenial. It is likely that the prearticular also articulates dorsally with part of the anterior ventral edge of the surangular, although the contact between these bones is not clear in the micro-CT images of PVL 4604. A substantial portion of its anteroposterior length contacts ventrolaterally the angular. The prearticular forms the internal wall of the mandibular adductor fossa, which is the only component of the post-dentary region of the hemimandible ventral to this opening. The prearticular contacts posteriorly the articular.

### 4.32. Articular

The left articular is more complete and better preserved than its right counterpart in the holotype of *T. romeri* (PVL 4601). The same applies to the right articular of PVL 4606, although, in this case, matrix partially covers its medial side. By contrast, in PVL 4604, both articular bones are mostly complete and well-preserved. The anterior region of the dorsal surface of the articular of this specimen is covered by its articulation with the quadrate. However, the virtual disarticulation of the jaw and skull in PVL 4604 allowed a more complete visualization of the dorsal surface of the articular. As mentioned earlier, it was not possible to recognize the articular–surangular suture. This is probably owing to the lack of resolution of the micro-CT rather than because of fusion between these two elements, but the possibility cannot be ruled out. The articular has a greater lateromedial development on the dorsal surface, tapering towards the ventral edge, displaying a V-shaped cross-section. The broadest lateromedial extension is located at the level of the craniomandibular joint (figure 30*b*,*d**,* glf), where two well-developed concave depressions (i.e. cotyles) are recognized, separated by a small crest. These cotyles received the pair of condyles of the quadrate. The medial cotyle is more posteriorly displaced than the lateral one. In dorsal view, a small lobe-shaped process projects medially (figure 30*e*,*f**,* lp) on the inner edge of the articular. In the same view, it can also be seen that the articular narrows posteriorly as a subrectangular retroarticular process (figure 30*a**,* rarp), much longer than in *Ch. bonapartei* (PULR-V 07; [36]), *Gu. reigi* (PVL 4576), *Pi. rodriguesi* (UFRGS-PV-0464-T; [16]) and *Ps. ischigualastensis* [16]. This contrasts with the absence of a retroarticular process in both species of *Proterochampsa* [33,42]. A distinct dorsomedial process on the retroarticular process is separated from the glenoid fossa by a short and distinctly concave surface.

## 6. Discussion

In this contribution, we thoroughly reviewed the four preserved skulls of *T. romeri* (PVL 4601, 4604, 4606, 4625), confirming that they are morphologically very similar to each other. However, anatomical differences indicate some variability in certain skull regions, which are discussed as follows.

The maximum dorsoventral height of the postorbital region of the skull is very similar in PVL 4601 and 4604—although PVL 4604 is slightly dorsoventrally compressed, with a small lateral displacement of the anterior end of the rostrum in the horizontal plane and the mandible towards opposite sides, and a strong lateral inclination of the ventral process of the right postorbital. In PVL 4606, the postorbital region of the skull is dorsoventrally shorter (10% less) than in PVL 4601 and 4604. The lack of fractures on the external surface of the bones and signs of deformation suggests that the condition present in PVL 4606 is more likely natural rather than a preservation artefact.

The dorsal surface of the parietals of the three more complete skulls of *T. romeri* (PVL 4601, 4604, 4606) possesses a pair of occipital crests with different orientations between them. In the holotype (PVL 4601), the occipital crests are anteroposteriorly oriented and parallel to the major axis of the skull. By contrast, in the referred specimens (PVL 4604 and 4606), the crests are slightly anteromedially-to-posterolaterally oriented, resembling the orientation of the posterolateral processes of the parietal.

The L-shaped quadratojugal of the holotype of *T. romeri* results from its vertical ascending process and horizontal anterior process. By contrast, PVL 4604 and 4606 have a slightly anterodorsally oriented ascending process of the quadratojugal. Nevertheless, it cannot be ruled out that this condition is caused by the same compression mentioned above. The quadratojugal of PVL 4606 possesses a third, well-developed process projected posteriorly, which obscures the ventral end of the quadrate in lateral view. This third process is very reduced or almost non-existent in the quadratojugal of the holotype and PVL 4604.

The unusual sinuous suture between the left postorbital and jugal of PVL 4604 contrasts with the straight suture on its right side. In PVL 4601, this suture is barely discernible, while in the well-preserved skull of PVL 4606 it is straight on both sides. This suggests that the sinuous left suture in PVL 4604 could be interpreted as the result of deformation and, originally, was probably straight. Another difference observed between the skulls involves the participation of the postorbital and jugal in the posterior and anterior borders of the orbit and infratemporal fenestra, respectively. In PVL 4606, the long extension of the descending process of the postorbital completely excludes the jugal from the posterior border of the orbit. The ascending process of the jugal occupies most of the anterior border of the infratemporal fenestra, with a small contribution of the postorbital. By contrast, there is a more sub-equal contribution of the postorbital and jugal to the posterior border of the orbit in PVL 4601 and 4604. The reverse condition occurs in the anterior border of the infratemporal fenestra, but in this case, the jugal is the predominant element in all cases.

The differences identified between the available skulls of *T. romeri* are minor and here interpreted as intraspecific variation. Thus, we agree with previous authors (e.g. [25,33]) that, so far, there is evidence of a single species of non-rhadinosuchine proterochampsid in the *Massetognathus-Chanaresuchus* AZ of the Chañares Formation.

Regarding the braincase of *T. romeri*, a previous precedent has provided new data regarding this region, which is poorly studied in Proterochampsidae. However, the resolution and contrast of the medical tomographic images were not enough to discern fine details sufficiently, and several structures remained unknown [39]. This contribution improved our understating of this region of the skull of *T. romeri* with a new digital reconstruction of the braincase based on micro-CT images. It is, to our knowledge, the first time that each bone of its neurocranium has been individually segmented. The recognition of an anterior tympanic recess on the lateral surface of the parabasisphenoid of *T. romeri* is particularly interesting. This feature has been previously interpreted as an apomorphy of dinosaurs, but now it is recognized among non-dinosaurian avemetatarsalians. Moreover, *T. romeri*, together with *Ch. bonapartei* [36] and *Eu. capensis* [72], broadens the presence of this feature among non-archosaurian eucrocopods.

Arcucci [25] erected the species *T. romeri* based on seven specimens from the Upper Triassic rocks of the Chañares Formation. The diagnosis for the species proposed by Trotteyn *et al.* [12] included several character-states, such as: the inclination of the quadrate and the well-developed occipital crests, which are suggested as distinctive features compared with the skull of the sympatric species *Ch. bonapartei* and *Gu. reigi* [25]. In the last decade, various contributions have improved and expanded the previous knowledge about different proterochampsid species [23,36,37] and a reassessment of the differences between *T. romeri* and the other species is needed. The skull of *T. romeri* is not the first to be studied using non-invasive methods, such as CT scans. However, it is the first Proterochampsidae from the Ischigualasto-Villa Unión Basin in which each cranial bone (or the vast majority) has been individually segmented. In this contribution, the meticulous osteological analysis of each preserved skull of *T. romeri* revealed several differences compared with other proterochampsids.

The skull of *T. romeri* is considerably different from that of *Proterochampsa*, but resembles those of rhadinosuchines. Nevertheless, *T. romeri* differs from rhadinosuchines and *Ce. binsfeldi* in the morphology of skull roof ornamentation, which consists of crests without a radial arrangement. The orbits are subcircular and represent the largest cranial openings. The alveolar margin of the premaxilla is not oriented anteroventrally. The maxilla is restricted to the lateral wall of the rostrum, and the moderate development of its horizontal process places its distal end between the anterior and posterior margins of the orbit. The rostrum is shorter anteroposteriorly, with the nasals broad laterally and slightly longer than the frontals. The jugal is very slender and gracile, and the frontals have highly developed anterolateral processes.

In addition, other features were recognized in the skull of *T. romeri* that, taken together, support its distinction from other proterochampsids. These include the absence of a fossa on the maxilla and lacrimal (a trait shared with *Ps. ischigualastensis*), the lack of depression or notch at the posteroventral corner of the infratemporal fenestra (a characteristic unknown in *Rh. gracilis*), the absence of a supratemporal fossa on the parietals (shared with both species of *Proterochampsa* and *Ce. binsfeldi*) and the presence of an anterior tympanic recess on the lateral surface of the parabasisphenoid (shared with *Ch. bonapartei*). It is important to note that an emended diagnosis will be included in a future contribution after the osteological revision of the postcranial skeleton of *T. romeri* is completed.

## 7. Conclusions

This contribution is the first in a series of future studies aimed to comprehensively revise the anatomy of *T. romeri*. Here, the redescription is focused exclusively on its cranial anatomy and is based mainly on the revision of the three most complete and best-preserved skulls of *T. romeri* (PVL 4601, 4604 and 4606). In addition, our redescription included a fourth, previously unpublished specimen (PVL 4625b).

The micro-CT scan of the skull of PVL 4604 allowed a detailed assessment of the internal and external morphology of each bone, as well as the identification of many previously unknown sutures (see Arcucci [25]). This study also includes the first digital reconstruction of a proterochampsid braincase with each bone segmented independently.

The available skulls of *T. romeri* have a very similar morphology to each other, which is congruent with what would be expected for a single species. Thus, the minimal differences that are not the result of deformation, are probably owing to intraspecific variability.

The skull of *T. romeri* presents a unique combination of character states that allow it to be distinguished from any other proterochampsid species. As a consequence, our observations support Arcucci’s [25] original proposal that *T. romeri* is a taxonomically valid species.

Finally, this detailed description of the skull provides a new source of information that will allow a comprehensive review of the characters scored in previous quantitative phylogenetic analyses (e.g. [16,36]) and a more robust assessment of the phylogenetic relationships of *T. romeri* among other proterochampsids.

## Data Availability

The three-dimensional models of the skull of PVL 4604, which were used as the basis for the figures in this manuscript, have been included as the electronic supplementary material [89]. Three-dimensional renderings of the different bones of specimen PVL 4604 that form the basis of the anatomical description of the skull of Tropidosuchus romeri and the photogrammetric models of the skull of PVL 4601, 4604 and 4606 that contributed to the descriptive work are available on Morphosource: https://www.morphosource.org/projects/000702164/temporary_link/sHCPtJVvdYeZ5GwAUh13WmMn?locale=en.
